# Independent external validation and head-to-head comparison of guideline-recommended CVD risk prediction models

**DOI:** 10.1016/j.ajpc.2026.101625

**Published:** 2026-04-11

**Authors:** Lum Kastrati, Eleftheria Maria Alexandri, Maurice Rupp, Lisa Koch, Jose Garcia-Tirado, Christos Nakas, David J. Maron, David Herzig, Lia Bally

**Affiliations:** a Department of Diabetes, Endocrinology, Nutritional Medicine and Metabolism, Inselspital, Bern University Hospital, University of Bern, Bern, Switzerland; b Laboratory of Biometry, School of Agriculture, University of Thessaly, Volos, Greece; c Diabetes Center Berne, Bern, Switzerland; d Department of Clinical Chemistry, Inselspital, Bern University Hospital, University of Bern, Bern, Switzerland; e Stanford Prevention Research Center, Stanford University School of Medicine, Stanford, CA, United States

**Keywords:** Cardiovascular disease, Clinical risk prediction models, Prevention, Personalized medicine

## Abstract

**Background:**

Cardiovascular disease (CVD) prediction models recommended by guidelines are developed using different populations, predictors, and outcome definitions. The implications of this heterogeneity for risk estimation are unclear, and direct comparisons remain limited.

**Objectives:**

Head-to-head comparison of the performance and transportability of three guideline-endorsed CVD risk prediction models, focusing on their sex-specific performance.

**Methods:**

We evaluated models recommended by the American Heart Association (PREVENT), European Society of Cardiology (SCORE2), and the National Institute for Health and Care Excellence (QRISK3). Risk of bias was assessed using the PROBAST tool. External validation was performed using the UK Biobank (UKBB) in a primary analysis including all participants with complete data for all models, enabling direct comparison, and in a secondary analysis applying each model to participants meeting its original eligibility criteria. Model performance was assessed using Brier scores, Area Under the Receiver Operating Characteristic Curve (AUC), and calibration across original and alternative outcome definitions, stratified by sex.

**Results:**

The PREVENT, SCORE2, and QRISK3 models varied substantially in terms of predictors, populations, and outcome definitions. We used data from 502,157 UKBB participants for the external validation in the primary analysis. Overall predictive performance (discrimination & calibration), as measured by Brier scores, was generally better in females. The AUC (95% CI) ranged from 0.7092 (0.7090–0.7094) to 0.7468 (0.7465–0.7471) for female and 0.6813 (0.6812–0.6814) to 0.6946 (0.6945–0.6946) for male populations. Calibration was suboptimal, particularly for older individuals, with systematic overestimation of risk. The models showed consistent performance when applied to different outcomes. All models were at high risk of bias.

**Conclusion:**

Despite heterogeneity in populations, predictors, and outcome definitions, PREVENT, SCORE2, and QRISK3 showed similar performance in the UKBB. Future studies should focus on prospective and standardized definitions and assessment of candidate predictors and outcomes.

## 1 Introduction

Cardiovascular diseases (CVD) remain the leading cause of death worldwide [1]. However, timely preventive interventions can reduce the incidence of CVD events, thereby decreasing CVD-related morbidity and mortality. CVD risk prediction models are essential in clinical practice for identifying individuals at risk, enhancing patient communication and awareness, and guiding evidence-based decision-making for preventive interventions. These models are also valuable for informing follow-up strategies, improving trial design (e.g., through risk based-stratification), and enabling cost-effectiveness analyses. A systematic review published in 2016 identified 212 studies describing the development of 363 CVD prediction models and 473 external validations [2]. With the growing availability of electronic health data and advances in analytical methods, it is likely that the number of such models has since grown substantially. Yet, only a few models are endorsed by current guidelines and routinely used in clinical practice to inform decision-making.

Despite sharing the overarching goal of predicting CVD events, these models vary in their eligibility criteria, included predictors, and outcome definitions, differences that affect their applicability and performance. To enable appropriate interpretation and effective use of these models in clinical, research, and public health contexts, it is essential to recognize their distinctions, limitations, and generalizability. Notably, the literature highlights the low prevalence of such *head-to-head* comparisons of established CVD risk prediction models [3].

Therefore, the present work aims to provide a comprehensive review of guideline-endorsed CVD risk prediction models. Leveraging the UK Biobank (UKBB) population-based cohort, we perform an independent external validation and head-to-head comparison, adhering to best practice standards for such evaluations [3]. Specifically, we evaluate model discrimination and calibration, assess the sensitivity of performance to alternative outcome definitions, and compare performance by sex. Uniquely, our work departs from traditional validation approaches by assessing model performance both within and beyond the original target populations, providing an in-depth appraisal of their applicability and transportability. For transparency, we report results from a primary analysis in the maximal common sample of participants with complete predictor data, and a secondary analysis applying each model to its original target population.

## 2 Methods

### 2.1 Selection criteria

The following original prediction models were included in the present work: CVD risk prediction models recommended for use by the most recent guidelines of the: (a) The National Institute for Health and Care Excellence from the United Kingdom [4] – *the QRISK3 model* [5], (b) European Heart Association [6] – *The SCORE2 models* [7], and (c) The American Heart Association [8] *- the PREVENT model* [9].

### 2.2 Data extraction

For each included CVD risk prediction model, we extracted the following data: study and population characteristics (year of publication, study design, number of participants, mean follow-up time, eligibility criteria), predictors, modelling approach, definition of CVD events, number of CVD events, and model performance metrics (calibration, Area Under the Receiver Operating Characteristic Curve (AUC) or C-statistic). Two authors (MR and LuK) independently extracted all the required data. Possible discrepancies were resolved by consulting a third author (LB).

### 2.3 Population for external validation

We validated the prediction models using data from the UKBB, a large-scale biomedical database. It comprises a long-term prospective cohort (baseline recruitment window from 2006-2010) from the United Kingdom with >500,000 participants from the general population. Its design is described elsewhere [10]. Data were collected through a combination of questionnaires, electronic health records (EHR), interviews, and biological samples. For our analysis, we used the baseline data of the participants and followed them until the end of the follow-up for up to 10 years. The end of follow-up was determined by the time of the first CVD event, death, or loss to follow-up, whichever happened first. Participants were censored at the last period of the follow-up or death from other causes, which define competing events. All UKBB participants provided informed consent, and the resource has received ethical approval from the relevant authorities. Therefore, no additional ethical approval was required for this study.

### 2.4 Eligibility criteria of the UKBB population used for external validation

For the primary analysis, we defined a common analytic sample restricted to participants eligible for all three models and with complete data on the required predictor variables, enabling a direct head-to-head comparison under consistent conditions. For the secondary analysis, we adapted the eligibility criteria of the population included in the development of the original model and applied them respectively for each of the included models. Participants with missing data on the independent variables were excluded from the analysis. The eligibility criteria of the populations of all included prediction models are presented in Table 1.

**Table 1:** Reported characteristics of the included CVD risk prediction models.

Study		Sample Size	CVD Incidence N (%)	Follow-up period Years Median (IQR)	Calibration	C-Statistics (95 % CI)	Inclusion criteria	Predictors included in the Prediction Model
QRISK 3	Derivation Cohort of the Female Population	4,019,956	160,549 (4 %)	4.4 years (interquartile range 1.6-10.8)	-	-	Free of CVD at baseline Were not using statins at baseline Have a Townsend deprivation score	Age SBP Townsend score Ethnic origin Smoking status Family history of CVD Type 1 Diabetes Type 2 Diabetes Treated hypertension Rheumatoid arthritis Atrial Fibrillation CKD Migraine Corticosteroid use SLE Erectile dysfunction Atypical antipsychotic use Severe mental illness Total cholesterol BMI
	Derivation Cohort of the Male Population	3,869,847	203,016 (5.2 %)		-	0.879 (0.878 to 0.880)		
	Validation Cohort of the Female Population	1,360,457	-	4.4 years (interquartile range 1.6-10.8)	4.7 % predicted risk 5.8 % Observed risk	-		
	Validation Cohort of the Male Population	1,310,841	-		6.4 % Predicted risk 7.5 % Observed risk	0.858 (0.856 to 0.859)		
SCORE2* **	SCORE2	677 684	30, 121 (4.4 %)	10.7 (5.0-18.6)	Only Visual Representation	0.67 (0.65–0.68) to 0.81 (0.76–0.86)	Individuals aged 40-69 Free of CVD at baseline Free of diabetes at baseline	Age Smoking status Total Cholesterol HDL-C SBP
	SCORE2- OP	28,503	10,089 (35 %)	13 (8.0-15.0)	Only Visual Representation	0.63 (0.61-0.65) to 0.67 (0.64-0.69)	Individuals aged ≥70 Free of CVD at baseline	Age Smoking status Total Cholesterol HDL-C SBP Diabetes
	SCORE2-Diabetes	229 460	43706 (19 %)	10.9 (6.8-11)	Only Visual Representation	0.64 (0.63-0.65) To 0.71 (0.7-0.72)	Had Type 2 Diabetes at baseline Free of CVD at baseline Individuals aged 40-69	Age Smoking status Total-C HDL-C SBP Diabetes Age at diabetes diagnosis HbA1c eGFR
PREVENT	Derivation Cohort of the Female Population	1,839,828	53,258 (2.9 %)	4.8±3.1	-	0.789 (0.778– 0.810)	Individuals aged 30-79 Free of CVD at baseline SBP 90-200 mm Hg Total Cholesterol 130-320 mg/dL HDL-C 20 -100 mg/dL BMI 18.5 - 40.0 kg/m^2^	Age Smoking Status SBP Diabetes Cholesterol eGFR HDL-C Cholesterol Lowering Medication BP lowering treatment
	Derivation Cohort of the Male Population	1,442,091	53,403 (3.7 %)	4.6±3.0	-	0.745 (0.734–0.760)		
	Validation Cohort of the Female Population	1,894,882	54,365 (2.9 %)	5.0±3.2	Calibration slope 1.03 (0.83-1.17)	0.794 (0.763–0.809)		
	Validation Cohort of the Male Population	1,435,203	50,489 (3.5 %)	4.8±3.2	Calibration slope 0.95 (0.85-1.13)	0.757 (0.727–0.778)		

**SBP** –Systolic Blood Pressure, **CKD**- Chronic Kidney Disease, **eGFR**- Estimated Glomerular Filtration Rate, **BMI-** Body Mass Index, **SLE-** Systemic Lupus Erythematosus. **CVD**- Cardiovascular Disease, **HDL-C** High Density Lipoprotein Cholesterol. **Total-C** – Total Cholesterol, **HbA1c-** Haemoglobin A1c. * SCORE2 provided 4 different models calibrated for specific geographical regions. **- The reported AUC-s of the SCORE-2 Models present the range of the AUCs across the various external validation datasets.

### 2.5 Outcome definition

Model performance was evaluated using two outcome definitions: (i) by matching the original study's outcome definition (e.g., SCORE2 model performance based on the SCORE2 outcome definition), and (ii) by applying the outcome definitions of other prediction models (e.g., SCORE2 model performance based on PREVENT and QRISK3 outcome definitions), to account for heterogeneity in outcome definitions. This approach allows us to assess model performance both within and beyond their originally intended outcome definitions. Detailed outcome definition and assessment for each study are provided in the Supplementary Appendix (Table SA2a-c).

#### 2.5.1 Assessment of the outcome in the UKBB

In the UKBB, the CVD outcome was assessed using self-reported questionnaires, or was extracted from EHR, namely the Hospital Episode Statistics from the UK (ICD10 & ICD9 codes), or using death registries. Detailed information is provided in the Supplementary Appendix (Table SA2a-c).

### 2.6 Assessment and definition of predictors

The list of predictors included in each prediction model is provided in Table 1. We matched the definition of the predictors from the original studies. The definition and assessment of predictors for each study and in the UKBB are described in Table SA1.

### 2.7 Risk of bias assessment

We used the PROBAST tool [11] to evaluate potential bias sources in individual studies that could impact the applicability of results concerning the intended use of the models. Two authors (MR and LuK) independently assessed the risk of bias of the presented models. Discrepancies were discussed to reach consensus. Following the PROBAST standards, if an evaluation was judged as high risk of bias (RoB) for at least one domain, overall risk was considered to be high. If the prediction model evaluation was unclear in one or more domains and was rated as low in the remaining domains, risk of bias was judged as unclear.

### 2.8 Model implementation procedure

Analyses were based on complete cases (with subjects having missing values in required inputs excluded) for implementing the selected models. Two complementary approaches were implemented. In the primary analysis, all three models were implemented in the maximal common sample of participants with all predictors available, ensuring direct comparability of model performance. In secondary analysis, each model was applied to participants meeting its original eligibility criteria, reflecting the populations for which the models were developed.

The selected CVD risk prediction models were implemented as follows. The QRISK3_2017 function from the QRISK3 R package was used to implement the QRISK3 algorithm, after applying the respective eligibility criteria [12]. The base equation of the PREVENT 10-year risk estimation model, developed for Total CVD (atherosclerotic CVD and heart failure) as described in [13] was selected for the present work, with predictors including total cholesterol, high-density lipoprotein cholesterol (HDL-C), systolic blood pressure (SBP), and body mass index (BMI) constrained to fall within specific value ranges. For the implementation of the SCORE2 model, the RiskScorescvd R package was used [14]. If a participant had type II diabetes and was between 40 and 69 years of age, their 10-year CVD risk was estimated using the SCORE2_Diabetes function. For those aged 40 to 69 without diabetes at baseline, the main SCORE2 model was applied. For participants aged 70 years and over, the SCORE2_Older_Persons function was applied. The functions were parameterized for the low-risk region, as the analyses were conducted using data from individuals living in the UK.

All analyses were conducted within a time-to-event framework, accounting for competing non-CVD death. Observed risks were estimated using cumulative incidence functions (CIF). Calibration was assessed at a 10-year horizon by comparing predicted risks with CIF-based observed risks. Continuous predictors were grouped into bins: age at recruitment (5-year bins) and SBP (20 mmHg bins), with the mean predicted risk within each bin plotted against observed risk. Calibration was quantified by fitting a linear regression of observed on predicted risk, with slope and intercept summarizing overall calibration. A slope of 1 and an intercept of 0 indicate perfect calibration. Decile-based calibration was also performed, with participants grouped into ten risk deciles, mean predicted and CIF-based observed risks calculated for each, and reference lines indicating perfect calibration. Numbers at risk per decile were recorded, and all calibration curves and decile-based plots are provided in the Supplementary Material (File B). Calibration curves were generated overall and stratified by age, SBP, and sex. Age and SBP were selected as readily available robust predictors of CVD risk, commonly used by practitioners in routine practice, with age being the strongest non-modifiable risk factor [15] and SBP as the most influential modifiable risk factor [16]. The calibration slopes and intercepts for each model are provided in Table 3.

Discrimination was assessed using a time-dependent area under the ROC curve (AUC) at 10 years, calculated with cumulative/dynamic definitions accounting for competing risks. This quantifies each model’s ability to distinguish between participants who did or did not experience the event. To evaluate transportability, all models were validated across different outcome definitions using a consistent statistical framework, isolating the impact of differences in outcome definitions from the underlying risk estimation methodology. Discrimination focuses on classification correctness, while calibration evaluates the reliability of probability estimates.

The models were validated and reported according to the TRIPOD guidelines [17]. All the models presented sex-specific coefficients. Therefore, model performance was separately assessed for females and males. Given that UKBB data was one of the original development cohorts of the SCORE2_main and SCORE2_diabetes models, using it for validation might lead to overoptimistic results. Thus, in a sensitivity analysis, we evaluated model performance (discrimination and calibration) using beta coefficients derived from training on all original development cohorts except UKBB.

Analyses were performed using R version 4.5.1 (The R Foundation for Statistical Computing, Vienna, Austria), along with the corresponding R packages associated with each CVD risk prediction algorithm. Additional technical details regarding the analyses are provided in the Supplementary Appendix (File C).

## 3 Results

Qualitative description of the included prediction models

### 3.1 Characteristics of the development cohorts of the included prediction models

The QRISK3 model was developed using data coming from primary care EHRs in the UK [5], while the SCORE2 model was developed using 44 cohorts included in the “Emerging Risk Factor Collaboration” and the UKBB [7,18]. The PREVENT model was developed using data from a global consortium consisting of 46 US-based observational research and EHRs [9], for which details can be found elsewhere [19]. Specific eligibility criteria of the cohorts used for the development of each of the models are presented in Table 1.

#### 3.1.1 Outcome definitions of the included prediction models

**Fig. 1:**
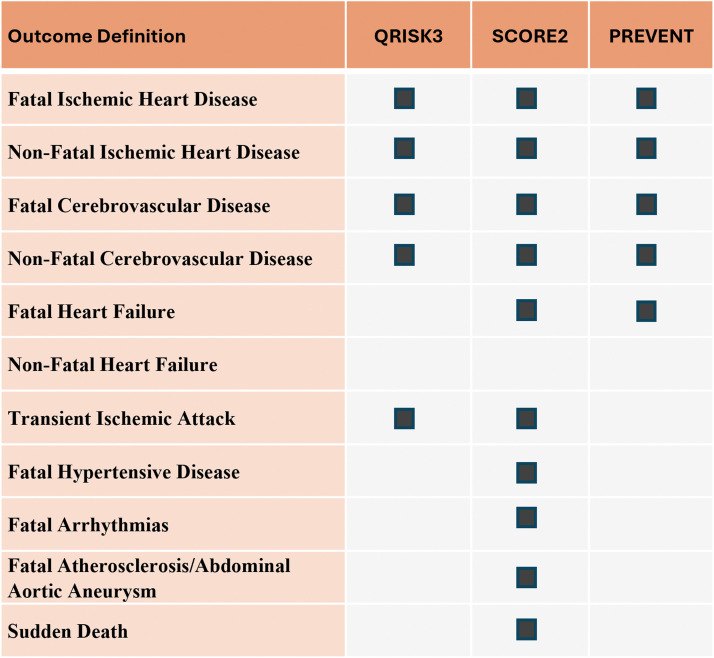
CVD outcome definition of the included prediction models.

All three models employed different outcome definitions for CVD events. Ischemic heart disease and cerebrovascular disease were included across all models (SCORE2 differentiated between fatal and non-fatal events). Other outcomes included (non) fatal heart failure, transient ischemic attack and fatal hypertensive disease, arrhythmias, atherosclerosis/abdominal aortic aneurysm, and sudden death, with various distributions across the three models. A general overview of outcome definitions for each of the models is provided in Fig. 1. Detailed information on outcome definition and their respective ICD10 codes is presented in the Supplementary Appendix (Table S2a-c)**.**

#### 3.1.2 Final prediction models

All three development studies of the selected CVD models presented multivariable prediction models. All models were derived using survival analysis. The QRISK3 and PREVENT models used Cox`s proportional hazards models to estimate the coefficients for the risk factors [20], while SCORE2 utilized Fine and Gray competing risk models [21]. PREVENT and SCORE2 models adjusted for competing risk of non-CVD deaths, while QRISK3 censored the participants at the event of a non-CVD death. Age, smoking status, markers of kidney function, diabetes, SBP, and total cholesterol were used across the three models. SCORE2 additionally reported specific models for Low, Medium, High and Very High risk regions in Europe. Characteristics of these studies as reported in the original articles are provided in Table 1. Detailed information on predictors’ assessment and definition are presented in the Supplementary Appendix (Table S1).

#### 3.1.3 Risk of bias assessment

**Fig. 2:**
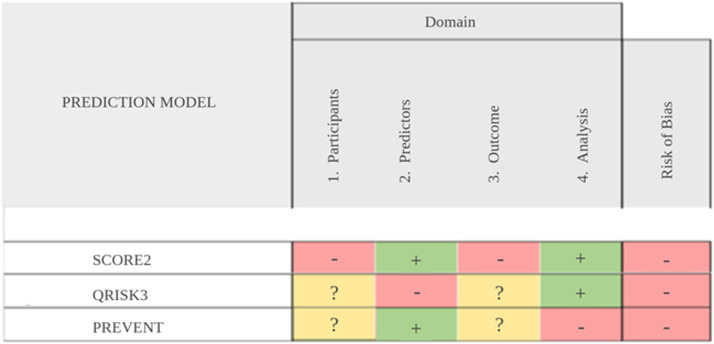
Risk of bias assessment.

All assessed models had at least one domain rated as high RoB (Fig. 2). None of the models was rated as low RoB in the *participants* domain. High or unclear RoB in this domain stemmed from eligibility criteria inconsistencies (SCORE2), or from using EHRs as data sources (QRISK3 and PREVENT). 12/45 cohorts used in the development of SCORE2 did not record non-fatal CVD events, despite being part of the outcome definition. This leads to misclassification bias: individuals who experienced non-fatal CVD events would have been misclassified as event-free and thus erroneously included in the analyses, contrary to the eligibility criteria. This misclassification could introduce bias in two ways. First, participants who actually had a non-fatal CVD event have a higher risk of fatal CVD events. Second, truly healthy individuals at baseline who later experienced non-fatal events may not have been captured, leading to false-negative classifications.

In the predictors’ domain, the QRISK3 was rated at high RoB. This was primarily due to certain predictors not being systematically assessed for all participants (e.g. learning disability, severe mental illness, atypical psychosis, erectile dysfunction). Moreover, when baseline data were not available, the most recent values recorded before the baseline date were used. This means that not all data were collected at the same time at baseline (exposure misclassification), and the estimates may not represent 10-year associations.

All models were rated a high or unclear RoB for the outcome domain. SCORE2 was rated at high RoB because the outcome was not uniformly assessed across all cohorts: 12/45 cohorts used for developing/validating the SCORE2 did not capture non-fatal CVD events. PREVENT and QRISK3 were listed as unclear bias because they use variable definitions of CVD events, which were based on EHRs or contained multiple non-standardized cohorts, which might have led to inconsistencies in outcome assessment/assignment. In the analysis domain, as per the PROBAST guidelines, the PREVENT study was listed as high RoB because the authors excluded participants with missing data. The authors of the QRISK3 study report that they censored for CVD events; however, we believe that this is a syntax error rather than methodological (the authors have been contacted for clarification).

External validation of QRISK3, SCORE2 and PREVENT models

We used data from 502,157 UKBB participants for the external validation. After applying each model’s specific eligibility criteria, 302,670 participants were eligible for the validation of the three models. Fig. SA1 (Supplementary Appendix) present baseline characteristics of the UKBB participants eligible for each model. The median follow-up time (with 5^th^ and 95^th^ percentiles) was 10.0 (10.0-10.0), and 10.0 (6.9-10.0) years for females and males, respectively. At the end of follow-up, 4.2 % of females and 8.5 % of males experienced the outcome using the QRISK3 prediction model and the respective outcome definition, 1.6 % and 3.7 % for the SCORE2 model, and 1.9 % and 4.2 % for the PREVENT model respectively (Table 2).

**Table 2:** Summary of the event rates and performance metrics for the implementation of the models across the three outcome definitions, separately for female and male population in the UKBB.

Algorithm	Outcome Definition	Females				Males			
		*AUC (95 % CI)*	*Brier score*	*N*	*Events%*	*AUC (95 % CI)*	*Brier score*	*N*	*Events %*
QRISK3	PREVENT	*0.7468 (0.7465-0.7471)*	*0.0150*	*170,613*	*1.85*	*0.6944 (0.6943-0.6945)*	*0.0301*	*132,057*	*4.15*
	SCORE2	*0.7405 (0.7402-0.7408)*	*0.0126*		*1.55*	*0.6909 (0.6908-0.6910)*	*0.0266*		*3.65*
	QRISK3	*0.7242 (0.7240-0.7244)*	*0.0320*		*4.24*	*0.6946 (0.6945-0.6946)*	*0.0555*		*8.45*
SCORE2 MAIN/OP/ DIABETES	PREVENT	*0.7367 (0.7365-0.7370)*	*0.0168*		*1.85*	*0.6857 (0.6856-0.6858)*	*0.0359*		*4.15*
	SCORE2	*0.7332 (0.7329-0.7335)*	*0.0141*		*1.55*	*0.6875 (0.6874-0.6876)*	*0.0316*		*3.65*
	QRISK3	*0.7092 (0.7090-0.7094)*	*0.0361*		*4.24*	*0.6813 (0.6812-0.6814)*	*0.0677*		*8.45*
PREVENT	PREVENT	*0.7433 (0.7431-0.7436)*	*0.0160*		*1.85*	*0.6865 (0.6864-0.6866)*	*0.0340*		*4.15*
	SCORE2	*0.7371 (0.7368-0.7374)*	*0.0134*		*1.55*	*0.6834 (0.6833-0.6835)*	*0.0300*		*3.65*
	QRISK3	*0.7140 (0.7138-0.7142)*	*0.0344*		*4.24*	*0.6824 (0.6823-0.6825)*	*0.0640*		*8.45*

### 3.2 Models’ performance within and across different outcome definitions

All three models demonstrated moderate performance both within and beyond their original outcome definitions, without major differences between the models. Brier scores suggested good overall statistical performance, ranging from 0.0126 to 0.0361 for females and from 0.0266 to 0.0677 for males. Models showed consistently better discrimination in female populations, with AUC values ranging from 0.7092 (0.7090–0.7094) to 0.7468 (0.7465–0.7471) for females, and 0.6813 (0.6812–0.6814) to 0.6946 (0.6945–0.6946) for male populations respectively. The QRISK3 model demonstrated the best discrimination in both sexes, with consistently higher AUC values across all three outcome definitions, and the lowest Brier scores, indicating better overall predictive accuracy. In contrast, the SCORE2 model showed the poorest discrimination, with the lowest AUC values across the PREVENT and QRISK3 outcome definitions, and the highest Brier scores across all outcome definitions, indicating inferior discrimination and overall predictive performance compared with the other models (Table 2). Results for SCORE2 remained consistent in the sensitivity analysis, where evaluation was performed by employing coefficients derived from datasets not containing the UKBB (Table SA5a).

### 3.3 Model calibration across female populations

**Fig. 3:**
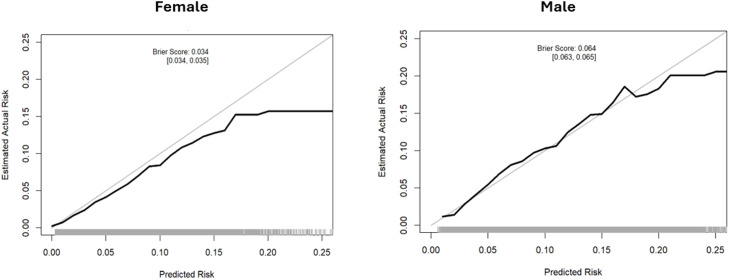
Calibration curves of the PREVENT model using the QRISK3 outcome definitions.

Visual inspection of the calibration curves indicated that all models tend to overestimate risk. The QRISK3 model consistently overestimated the risk under the SCORE2 and PREVENT outcome definitions, while demonstrating slightly better calibration using its own outcome definition. This pattern persisted when assessing the calibration curves for age and SBP. Similarly, the PREVENT model overestimated the risk, also when calibration was assessed for age and SBP, using the PREVENT and SCORE2 outcome definitions. In contrast, the PREVENT model demonstrated better calibration under the QRISK3 outcome definition, with the predicted values aligning closer to the observed outcomes overall, and for ages <55 years and SBP of <160 mmHg. This combination yielded the best calibration across all model-outcome combinations (Fig. 3). The SCORE2 model displayed inconsistent calibration. Slight risk overestimation for its own and PREVENT outcome definitions, contrasted by risk underestimation for the QRISK3 outcome definition, particularly for participants older than 60.

These findings are broadly consistent with the calibration metrics as reflected by the corresponding overall slopes and intercepts for each model and outcome definition presented in Table 3. In the QRISK3 model, the QRISK3 outcome showed a slope closest to 1, suggesting somewhat better agreement between predicted and observed risks, whereas the SCORE2 outcome had the lowest intercept, indicating less pronounced overestimation. In the PREVENT model, agreement was also better with the QRISK3 outcome, while the intercept was closest to zero for the SCORE2 outcome. In the SCORE2 model, the slope was closest to 1 under the QRISK3 outcome, whereas the intercept was closest to zero for the SCORE2 outcome. Notably, this was also the only model–outcome combination showing risk underestimation, while all others predominantly overestimated risk. Overall, no model achieved ideal calibration. Importantly, visual inspection of the calibration curves indicated that the PREVENT model evaluated with the QRISK3 outcome definition showed the best overall calibration for females, followed by the SCORE2 model with the QRISK3 outcome definition, whereas the slope and intercept metrics provide a complementary but less discriminating summary of calibration performance.

**Table 3:** Overall Calibration Slope and Intercept (with 95 % Confidence Intervals) for Each Combination of Algorithm and Outcome Definition.

Mean (95% CI)							
Algorithm	Outcome definition	PREVENT		SCORE2		QRISK3	
		Women	Men	Women	Men	Women	Men
QRISK3							
*Slope*	0.144 (0.119‒0.169)	0.142 (0.124‒0.160)	0.104 (0.086‒0.123)	0.127 (0.111‒0.144)	0.207 (0.171‒0.243)	0.249 (0.217‒0.281)	
*Intercept*	0.037 (0.026‒0.048)	0.040 (0.031‒0.048)	0.030 (0.022‒0.039)	0.035 (0.027‒0.044)	0.070 (0.054‒0.086)	0.076 (0.060‒0.092)	
PREVENT							
*Slope*	0.287 (0.243‒0.332)	0.278 (0.235‒0.320)	0.227 (0.194‒0.260)	0.233 (0.194‒0.272)	0.413 (0.342‒0.484)	0.448 (0.371‒0.525)	
*Intercept*	0.018 (0.008‒0.027)	0.030 (0.019‒0.040)	0.014 (0.007‒0.022)	0.028 (0.018‒0.038)	0.041 (0.025‒0.056)	0.061 (0.042‒0.080)	
SCORE2							
*Slope*	0.453 (0.351‒0.554)	0.329 (0.245‒0.414)	0.408 (0.314‒0.501)	0.342 (0.261‒0.422)	0.683 (0.511‒0.855)	0.611 (0.469‒0.753)	
*Intercept*	0.015 (0.002‒0.028)	0.036 (0.020‒0.051)	0.012 (0.000‒0.025)	0.030 (0.015‒0.045)	0.035 (0.013‒0.058)	0.064 (0.038‒0.091)	

All models achieved their lowest Brier scores when evaluated using the SCORE2 outcome definition, indicating better overall predictive performance under this outcome definition despite the calibration issues. The calibration curves are presented in the Supplementary Material (Calibration_Curves file, File B). Sensitivity analysis showed similar calibration for the SCORE2 models when validated using coefficients derived from cohorts excluding UKBB, indicating consistency with the main analysis (Table SA5b).

### 3.4 Model calibration across male populations

Similarly, visual inspection of the calibration curves indicated a tendency toward risk overestimation in most cases across all outcome definitions. We consistently observed sharp declines in calibration at older ages and higher values of SBP. The QRISK3 model consistently overestimated the predicted risk under the SCORE2 and PREVENT outcome definitions, while showing slightly better calibration using its own outcome definition, as observed in females. However, miscalibration remained clearly evident both overall and across most age and SBP strata. The PREVENT model again showed the best model calibration using the QRISK3 outcome definition, while it overestimated the risk for the other outcome definitions (Fig. 3). The SCORE2 model slightly overestimated the risk under the PREVENT and SCORE2 definitions but underestimated the risk under the QRISK3 outcome definition, especially for participants older than 60 years.

These graphical observations are broadly consistent with the overall slopes and intercepts presented in Table 3. For males, the slope was generally closest to 1 for the QRISK3 outcome, suggesting somewhat better proportional calibration, while the intercept was nearest to zero for the SCORE2 outcome, reflecting slightly reduced systematic overestimation. Overall, no model achieved perfect calibration. Visually, the PREVENT model evaluated with the QRISK3 outcome definition demonstrated the most acceptable calibration in males, whereas slope and intercept metrics provide a complementary but less detailed summary of calibration performance.

Again, all models achieved their lowest Brier scores when evaluated using the SCORE2 outcome definition, indicating better overall predictive performance under this outcome definition. The calibration curves are provided in the Supplementary_Material_Calibration_Curves file (File B). Sensitivity analyses showed consistent calibration results for the SCORE2 models when validated using coefficients derived from cohorts excluding UKBB (Table SA5a-b).

### 3.5 Secondary analysis: model performance in original target populations

Broadly, the results of the secondary analyses were consistent with those of the primary analysis in terms of both discrimination and calibration. Specifically, AUC values ranged from 0.7048 (0.7048–0.7049) to 0.7438 (0.7436–0.7440) in females and from 0.6750 (0.6750–0.6750) to 0.6998 (0.6996–0.6999) in males, generally slightly lower than in the primary analysis, with the exception of the QRISK3 model, where AUCs were higher than in the primary analysis. Brier scores ranged from 0.0132 to 0.0424 in females and from 0.0276 to 0.0774 in males, slightly higher than in the primary analysis. Calibration patterns, overall and stratified by age and systolic blood pressure, were consistent with the primary analysis. The PREVENT model using the QRISK3 outcome definition showed the best calibration, while the SCORE2 model with the PREVENT outcome definition also demonstrated acceptable calibration in both sexes. Sensitivity analyses for the SCORE2 model, using beta coefficients derived from all original development cohorts except UKBB, were consistent with the main results for SCORE2 in the secondary analysis, analogous to what was observed in the primary analysis. All results from the secondary analyses can be found in the Supplementary Material (Files D–E).

## 4 Discussion

### 4.1 Overall model performance and calibration

In this independent external validation and head-to-head comparison of three guideline-recommended CVD prediction models, we observe vast heterogeneity among the predictors used and CVD outcome definitions. Upon external validation using the UKBB, we observe similar discriminatory performance across models. Overall, the models performed better for females, as indicated by lower Brier scores and higher AUC values. The AUCs ranged from 0.7092 (0.7090–0.7094) to 0.7468 (0.7465–0.7471) for female and 0.6813 (0.6812–0.6814) to 0.6946 (0.6945–0.6946) for male populations, respectively. The highest AUC values were observed for the QRISK3 model under the PREVENT outcome definition in females, and under the QRISK3 outcome definition in males. Notably, all models performed similarly even when tasked to predict outcomes slightly divergent from those for which they were originally developed.

In contrast, visual inspection of calibration curves demonstrates that models tend to overestimate risk, which might lead to overtreatment of some patients. The PREVENT model, when evaluated using the QRISK3 outcome definition, showed relatively better calibration upon visual inspection. Regarding overall predictive performance, the lowest Brier score was observed for the QRISK3 model under the SCORE2 outcome definition, while across all models, predictions using the SCORE2 outcome definition consistently achieved the lowest Brier scores in both females and males, reflecting superior accuracy. Declines in calibration were particularly evident with increasing age and higher systolic blood pressure, suggesting reduced agreement between predicted and observed risks in these subgroups.

The observed suboptimal calibration might reflect differences between the UKBB and the original model development cohorts. The UKBB cohort includes considerably older participants and/or has longer follow up in comparison to the other cohorts (Table SA3a-c). Furthermore, differences in the underlying data sources could explain the systematic risk overestimation by PREVENT and QRISK3. The UKBB is a population-based cohort, thus, it is subject to *healthy-user-bias*, where participants might have different health seeking behavior - leading to a healthier population than the general population, as shown by previous studies [22]. Unlike prospective research-derived cohorts, PREVENT and QRISK3 models leveraged EHR data susceptible to *informed-presence-bias*, a selection bias that occurs when individuals with more health concerns are overrepresented. Participants are not included in EHR randomly, but due to their health status. As a result, these populations generally have a higher baseline risk for adverse health outcomes than the general population [23]. Therefore, prediction models developed from EHR populations will link the prediction estimates more aggressively to the outcome. This is also shown in our data (Tables SA3a-c). The cumulative incidence of CVD in UKBB is significantly lower than in the original development cohorts of the three models. When compared to the SCORE2 model, which has a somewhat comparable follow-up time, CVD incidence in UKBB was 2.7 %, while in the SCORE2 model development population was 4.4 %. In comparison to QRISK3 and PREVENT, a similar proportion of participants developed the outcome in those cohorts despite having approximately half the follow-up time of UKBB.

### 4.2 Other external validation attempts

The QRISK3 model was recently validated using the UKBB data with similar results to ours (C-statistic 0.722 in female and 0.697 in male), and the calibration curves showing risk overestimation [24]. The slight discrepancies might stem from the authors imputing missing data, while we proceeded with a complete case analysis approach.

Other studies validating the SCORE2 model used a Russian cohort of 7251 participants aged from 40 to 69 years. While the authors do not report any of the traditional performance measurements, the authors claim that the model demonstrated better performance in men [25].

SCORE2 validation in the EPIC-Norfolk cohort study showed slightly better performance for female than for men—C-statistics (95 % CI) of 0.78 (0.76–0.78) vs. 0.72 (0.69–0.74) [26]. In another validation using the REDISCOVER study from Malaysia, the authors validated all SCORE2 models (low, moderate, high and very high-risk models) by subgrouping participants into younger than 50, or 50 to 69 and then evaluated the model performance across sex. Interestingly, the model performed better for males in participants younger than 50, and better for females in older than 50 across all risk categories [27].

In another cohort study in Canada, consisting of 57,409 adults, the *C*-statistic for SCORE2 in females was 0.74 (0.72–0.76), better than in males 0.69 (0.67–0.71), with overall good calibration for males and females [28].

A study claiming to validate the PREVENT study, reported excellent performance of the models with C-statistics of 0.873 (0.860-0.886) for males and 0.904 (0.892-0.916) for females. However, this study only used fatal CVD events as an outcome and omitted the non-fatal CVD outcomes, for which PREVENT was developed as well [13].

### 4.3 Sex differences in model performance

Observed sex differences in model performance may stem from various biological and methodological factors, which merit exploration in the models’ development cohorts and in the UKBB. For instance, the regression coefficients appear to be more strongly associated with outcomes in females than in males. In the PREVENT study, type II diabetes was associated with a hazard ratio for CVD of 2.39 (2.31–2.48) in females and 2.18 (2.08–2.29) in males (i.e., ≈ 10 % higher risk). However, men had a higher prevalence of diabetes. Similarly, females diagnosed with systemic lupus erythematosus (SLE), severe mental illness or who were using atypical antipsychotics had a greater risk of developing CVD than their male counterparts. Several reasons could explain these differences. Females might also report data of higher quality. For example, in QRISK3, females had fewer missing data on independent variables, providing a more complete dataset for model training and more stable parameter estimates (larger signal-to-noise ratio). Additionally, conditions like SLE are more prevalent in females, again allowing for more robust estimations. Another relevant factor is the differential incidence of CVD across the lifespan. Because premenopausal females have a lower risk of CVD than age-matched males, more males may have been excluded at baseline due to prevalent disease. This could result in a more representative sample of incident cases among females in the cohort. The average age of females at baseline in these models (e.g., ∼42 years in QRISK3, 53 years in PREVENT, 57 years in SCORE2) often coincides with perimenopause, a period of rapid cardiometabolic changes. Algorithms may more swiftly capture these shifts, improving prediction performance [29]. Moreover, peri-menopausal women may engage more with healthcare services due to climacteric symptoms, leading to higher data completeness and lower recall bias. Other socio-demographic factors could also play a role in these differences [30].

### 4.4 Limitations of our work

First, this was not a systematic search of the current available CVD risk prediction models across the literature. There might be other models with better transportability that were not captured by our inclusion criteria. However, since these models are the most used ones in the recent guidelines, we believe that the assessment of these models is the most impactful in terms of real-life applicability. Other limitations are inherent to the UKBB dataset.

UKBB, as the name suggests, exclusively comprises UK participants – a limited scope sharply contrasted by broader racial and ethnic diversity within the other CVD model development cohorts assessed in our analysis. Moreover, UKBB’s restriction to ages 40-69 introduces significant uncertainty regarding model applicability to populations falling outside of this range.

Another limitation pertinent is that the SCORE2 model has used the UKBB among the other 44 datasets used for the original development and validation of the model. Therefore, we have to acknowledge that our results for the SCORE2 model may not fully represent external validation. Our sensitivity analysis employing coefficients derived from all cohorts but UKBB, demonstrated comparable discrimination and calibration, with only slightly lower AUC values observed in both females and males across all models and outcome definitions in both primary and secondary analyses. In addition, inherent to every validation study, the assessment of the predictors and the outcome differed between our dataset and the development datasets, potentially leading to different baseline survival hazards and estimates.

Finally, a further limitation relates to missing data. Analyses were restricted to participants with complete data for each model’s predictors, which may introduce selection bias if missingness is not at random. Although we report missing data proportions per predictor stratified by sex (Supplementary File A, Table SA4), we did not perform multiple imputation due to the substantial computational burden and prior evidence suggesting minimal impact on discrimination and calibration. However, we cannot exclude the possibility that excluding individuals with incomplete data influenced model performance estimates, and our findings should be interpreted in light of this consideration.

### 4.5 Implications for clinical practice and future research

Our study highlights the inconsistency of CVD event definitions used in risk prediction models. While this variation might partly reflect the lack of a consensus definition for CVD outcomes, we believe that it more likely stems from differences in data availability across studies. Interestingly, even though all models used different CVD outcome definitions, the performance of the models did not vary significantly when the models were used to predict slightly different CVD outcomes than their “original” outcome definitions, highlighting the transportability of these models. In real-world practice—where time, data availability, and patient complexity may limit the ability to apply tailored models—clinicians may reasonably rely on any of these models without substantial compromise in predictive performance, depending on the availability of predictors at the time of intended use. As shown in the present work, the models consistently overestimated the predicted risk in the general population; thus, recalibration of the models and a revision of the suggested treatment thresholds might be worth revisiting.

To advance CVD risk prediction, we propose the development of methodologically rigorous prospective cohorts, unified in design, with standardized definitions and assessments of candidate predictors and the outcomes of interest. Incorporating regular follow-ups, diverse populations, and broad age representation would yield more accurate and individualized risk estimation.

## Declaration of generative AI use

During the preparation of this work the authors used generative AI in order to enhance readability and language, aiding in formulating and structuring content. The authors reviewed and edited the content as needed and take full responsibility for the content of the published article.

**Figure:**
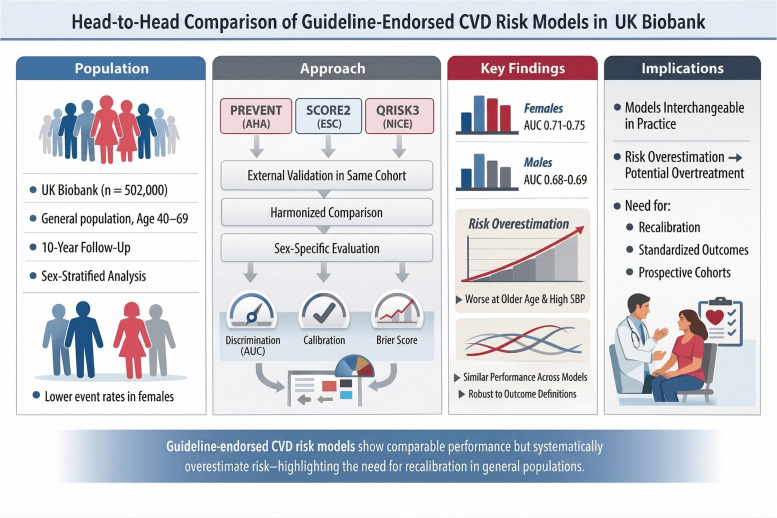
Central illustration.

## Funding

The present work was funded by Strategie-Förderungsgrant of the Medical Faculty of the University of Bern.

## CRediT authorship contribution statement

**Lum Kastrati:** Writing – review & editing, Writing – original draft, Visualization, Methodology, Formal analysis, Data curation, Conceptualization. **Eleftheria Maria Alexandri:** Writing – review & editing, Methodology, Formal analysis, Data curation. **Maurice Rupp:** Writing – review & editing, Data curation. **Lisa Koch:** Writing – review & editing, Funding acquisition. **Jose Garcia-Tirado:** Writing – review & editing, Funding acquisition. **Christos Nakas:** Writing – review & editing, Writing – original draft, Visualization, Supervision, Methodology, Formal analysis, Data curation, Conceptualization. **David J. Maron:** Writing – review & editing. **David Herzig:** Writing – review & editing, Writing – original draft, Visualization, Supervision, Methodology, Formal analysis, Conceptualization. **Lia Bally:** Writing – review & editing, Writing – original draft, Supervision, Methodology, Funding acquisition, Formal analysis, Conceptualization.

## Declaration of competing interest

The authors declare the following financial interests/personal relationships which may be considered as potential competing interests:

Lia Bally reports a relationship with Eli Lilly and Company that includes: consulting or advisory and speaking and lecture fees. Lia Bally reports a relationship with Dexcom Inc that includes: consulting or advisory, funding grants, and speaking and lecture fees. Lia Bally reports a relationship with Novo Nordisk Inc that includes: consulting or advisory. Lia Bally reports a relationship with Ypsomed AG that includes: consulting or advisory, funding grants, and speaking and lecture fees. Lia Bally reports a relationship with Roche that includes: consulting or advisory. Lia Bally reports a relationship with Oviva that includes: consulting or advisory. Lia Bally reports a relationship with Sanofi SA that includes: consulting or advisory. All honoraria were allocated exclusively to the institution according to local policies. If there are other authors, they declare that they have no known competing financial interests or personal relationships that could have appeared to influence the work reported in this paper.

## Appendix Supplementary materials

Image, application 1

Image, application 2

Image, application 3

Image, application 4

Image, application 5
