# Supplementary material for: Independent external validation and head-to-head comparison of guideline-recommended CVD risk prediction models
Source: Am J Prev Cardiol. 2026 Apr 11;28:101625. doi: 10.1016/j.ajpc.2026.101625 (PMC13326136; doi:10.1016/j.ajpc.2026.101625)
Supplement: Supplementary file 2 [file mmc2.docx]

**Table SΑ1.** Assessment and definition of predictors in the original studies and in the UKBB

| **Study** | **Predictors** | **Original publication** | **Self Reported Data Field Code (FID20002)** | **ICD9 and ICD10** | **Tretment and Medication**  **Data Field Code**  **( FID 20003)** | **Other data fields** |
| --- | --- | --- | --- | --- | --- | --- |
| QRISK 3 | Age | Year |  |  |  | FID 21022 - Age at recruitment |
|  | Ethnic origin | 1= White or not recorded  2= Indian  3= Pakistani  4= Bangladeshi  5= Other Asian  6= Black Caribbean  7= Black African  8 = Chinese  9 = Other |  |  |  | FID 21000  1 = White, British, Irish, Any other white background  2 = Indian  3 = Pakistani  4 = Bangladeshi  5 = Any other Asian background  6 = Caribbean  7 = African  8 = Chinese  9 = Other |
|  | Deprivation index | Townsend score |  |  |  | FID 22189- Townsend score |
|  | Systolic blood pressure | Mm/Hg  Standard Deviation of the most recent values recorded before the baseline date. |  |  |  | Mean of all measurements found in FID 4080 (automated reading) and FID 93 (manual reading) Two measures of blood pressure were taken a few moments apart |
|  | BMI | Kg/m2  The most recent values recorded before the baseline date. |  |  |  | FID 21001 - Body mass index |
|  | Total cholesterol | HDL cholesterol ratio |  |  |  | FID 23406 (HDL Cholesterol) / FID 30690 (Cholesterol) |
|  | Smoking Status | 1= Non-smoker  2= Former smoker  3= Light smoker (1-9/day)  4= Moderate smoker (10-19/day)  5= Heavy smoker (≥20/day) |  |  |  | 1 = non-smoker (FID 20116 (Smoking status) = 'Never' or FID 22506 (Tobacco smoking) = 'Never smoked') 2 = ex-smoker (FID 20116 (Smoking status) = 'Previous' or FID 22506 (Tobacco smoking) = 'Ex-smoker') 3 = light smoker (FID 20116 (Smoking status) = 'Current' or FID 22506 (Tobacco smoking) = 'Occasionally' or 5 <= FID 20161 (Pack years of smoking) < 10) 4 = moderate smoker (10 <= FID 20161 (Pack years of smoking) < 20) 5 = heavy smoker (FID 22506 (Tobacco smoking) = 'Smokes on most or all days' or FID 20161 (Pack years of smoking) >= 20) |
|  | Family History | Family history of coronary heart disease in a first degree relative aged less than 60 years |  |  |  | FID 20107 (Illnesses of father) or FID 20110 (Illnesses of mother) = 'Heart disease' |
|  | Diabetes | Type 1  Type 2  No information on how diabetes was diagnosed. | 1220  1221  1222  1223 | E10-14  O24 | 1140868902, 1140874646, 1140874744, 1140883066, 1140884600, 1140909780, 1141152590, 1141157284, 1141168660, 1141171646, 1141177600, 1141180722, 1141180734, 1141189090 | FID 2976 (Age diabetes diagnosed) is smaller than FID 21022 (Age at recruitment), FID 4041 (Gestational diabetes only) = 'Yes', FID 2443 (Diabetes diagnosed by doctor) = 'Yes', FID 30750 (HbA1c) >= 48, FID 30740 (Glucose) >= 11.1, FID 6153 (Medication for cholesterol, blood pressure, diabetes, or take exogenous hormones) = 'Insulin’, FID 6177 (Medication for cholesterol, blood pressure or diabetes) = 'Insulin’ |
|  | Treated Hypertension | Diagnosis of hypertension  Treatment with at least one antihypertensive drug | 1065 | I10, I12, I13, I15 | 1140851138, 1140860308, 1140860312, 1140860316, 1140860332, 1140860340, 1140860342, 1140860356, 1140860404, 1140860418, 1140860426, 1140860454, 1140860470, 1140860478, 1140860562, 1140860610, 1140860690, 1140860696, 1140860714, 1140860728, 1140860750, 1140860764, 1140860790, 1140860802, 1140860806, 1140860882, 1140860904, 1140860972, 1140861088, 1140861090, 1140861128, 1140861138, 1140861190, 1140861276, 1140864950, 1140864952, 1140866078, 1140866122, 1140866128, 1140866136, 1140866236, 1140866248, 1140866446, 1140866450, 1140866738, 1140866756, 1140866800, 1140871986, 1140875934, 1140879760, 1140879762, 1140879778, 1140879798, 1140879802, 1140879806, 1140879818, 1140879842, 1140879854, 1140881702, 1140881728, 1140883468, 1140888510, 1140888552, 1140888556, 1140888560, 1140888646, 1140909368, 1140909708, 1140910442, 1140910512, 1140910606, 1140916356, 1140916362, 1140917428, 1140923336, 1140923572, 1140923788, 1140926778, 1140926780, 1140928284, 1141145660, 1141145668, 1141146124, 1141146126, 1141146128, 1141151016, 1141151018, 1141152998, 1141153026, 1141153328, 1141156836, 1141164276, 1141165470, 1141166006, 1141171152, 1141171336, 1141172492, 1141172682, 1141172698, 1141173888, 1141179974, 1141180592, 1141180598, 1141180778, 1141187788, 1141187790, 1141188790, 1141190160, 1141193282, 1141194794, 1141194800, 1141194804, 1141194808, 1141194810, 1141195254, 1141195258, 1141201038, 1141201040,6135 | FID 6153 (Medication for cholesterol, blood pressure, diabetes, or take exogenous hormones) = 'Blood pressure medication' or  FID 6177 (Medication for cholesterol, blood pressure or diabetes) = 'Blood pressure medication' |
|  | Rheumatoid arthritis | Diagnosis of rheumatoid arthritis  Felty’s syndrome  Caplan’s syndrome,  Adult onset Still’s disease  Inflammatory polyarthropathy not otherwise specified | 1464 | M05,  M06,  M06.1,  M06.4 |  |  |
|  | Atrial Fibrilation | Including atrial fibrillation  Atrial flutter  Paroxysmal atrial fibrillation | 1471 | I48 |  |  |
|  | CKD | Chronic kidney disease stage 3 4 or 5)  Chronic kidney disease stage 4  Chronic kidney disease stage 5  Nephrotic syndrome  Chronic glomerulonephritis  Chronic pyelonephritis  Renal dialysis  Renal transplant |  | N18.3  N18.4  N18.5  N04  N03  N11  Z99.2  Z94.0 |  |  |
|  | Migraine | Classic migraine  Atypical migraine  Abdominal migraine  Cluster headaches  Basilar migraine  Hemiplegic migraine  Migraine with or without aura | 1265 | G43 |  | FID 120016 (Ever had migraine) = ‘Yes’ |
|  | Corticosteroid use | Oral or parenteral prednisolone  Betamethasone  Cortisone  Depo-medrone  Dexamethasone  Deflazacort  Efcortesol  Hydrocortisone  Methylprednisolone  Triamcinolone |  |  | 1140853854, 1140857656, 1140858338, 1140862572, 1140865136, 1140868426, 1140868532, 1140868618, 1140874790, 1140874816, 1140874896, 1140874930, 1140874976, 1140875668, 1140875684, 1140876036, 1140876044, 1140876046, 1140876456, 1140878562, 1140879922, 1140879934, 1140881938, 1140882152, 1140882622, 1140882764, 1140882766, 1140882774, 1140882780, 1140882794, 1140882822, 1140882824, 1140882830, 1140882836, 1140882840, 1140882842, 1140882844, 1140882846, 1140882848, 1140882850, 1140882864, 1140882888, 1140882894, 1140882896, 1140882902, 1140882906, 1140882908, 1140882914, 1140882918, 1140883026, 1140883058, 1140883060, 1140883062, 1140883064, 1140884654, 1140884672, 1140884704, 1140884726, 1140888074, 1140888098, 1140888134, 1140888172, 1140888176, 1140888184, 1140909786, 1140910424, 1140910634, 1140910674, 1141145782, 1141151424, 1141157294, 1141157402, 1141157418, 1141162532, 1141164086, 1141167174, 1141169844, 1141173346, 1141174512, 1141174548, 1141179072, 1141179982, 1141180342, 1141181062, 1141181610, 1141189464, 1141191748, 1141194840, 1141195232, 1141195280 |  |
|  | Systemic Lupus Erythematosus | Diagnosis of SLE, Disseminated SLE or Libman-Sacks disease | 1381 | M32 |  |  |
|  | Second generation “atypical” antipsychotic use | Including amisulpride  Aripiprazole  Clozapine  Lurasidone  Olanzapine  Paliperidone  Quetiapine  Risperidone  Sertindole  Zotepine |  |  | 1140867420, 1140867444, 1140868170, 1140879658, 1140910358, 1140927956, 1140928916, 1141152848, 1141153490, 1141169714, 1141195974 |  |
|  | Diagnosis of severe mental illness | Psychosis  Schizophrenia  Bipolar affective disease | 1289, 1291 | F29  F20  F31 |  |  |
|  | Diagnosis of erectile dysfunction or treatment for erectile dysfunction | Erectile dysfunction  BNF chapter 7.4.5 including alprostadil, phosphodiesterase type 5 inhibitors, papaverine, or phentolamine | 1518 | N52, N48.8 | 1140869100, 1140883010, 1141168936, 1141187810, 1141192248 |  |
| PREVENT | Total cholesterol | Total cholesterol (mmol/L) |  |  |  | FID 30690 (Cholesterol) |
|  | Ethnicity | 1= White  2= Black  3= Hispanic  4= Asian  5= Other or missing |  |  |  | FID 21000  1 = White, British, Irish, Any other white background  2 = Caribbean, African  3 = Others  4 = Pakistani, Bangladeshi, Chinese, Any other Asian background  5 = Missing |
|  | HDL-Cholesterol | HDL cholesterol (mmol/L) |  |  |  | FID 23406 (HDL cholesterol) |
|  | Systolic blood pressure | Systolic blood pressure (mmHg) |  |  |  | Mean of all measurements found in FID 4080 (automated reading) and FID 93 (manual reading). Two measures of blood pressure were taken a few moments apart. |
|  | Diabetes mellitus | Glycated hemoglobin A1c ≥6.5% or fasting glucose ≥7.0 mmol/L (≥126 mg/dL) or non-fasting glucose ≥11.1 mmol/L (≥200 mg/dL) or use of glucose lowering drugs (ADA 2010 criteria)  Self-report of physician diagnosed diabetes.  Identification at any time during study period (from baseline to follow-up). | 1220  1221  1222  1223 | E10-14  O24 | 1140868902, 1140874646, 1140874744, 1140883066, 1140884600, 1140909780, 1141152590, 1141157284, 1141168660, 1141171646, 1141177600, 1141180722, 1141180734, 1141189090 | FID 2976 (Age diabetes diagnosed) is smaller than FID 21022 (Age at recruitment), FID 4041 (Gestational diabetes only) = 'Yes', FID 2443 (Diabetes diagnosed by doctor) = 'Yes', FID 30750 (HbA1c) >= 48, FID 30740 (Glucose) >= 11.1, FID 6153 (Medication for cholesterol, blood pressure, diabetes, or take exogenous hormones) = 'Insulin’, FID 6177 (Medication for cholesterol, blood pressure or diabetes) = 'Insulin’ |
|  | Smoking Information | 1= Current  0= Previous/Never |  |  |  | FID 20116 (Smoking Status) 1 = Current  0 = Previous, Never, Prefer not to answer |
|  | Medications for hypertension | Taking any medications prescribed for lowering blood pressure. In administrative data they used ATC codes: C02, C03, C07, C08, C09 |  |  | 1140851138, 1140860308, 1140860312, 1140860316, 1140860332, 1140860340, 1140860342, 1140860356, 1140860404, 1140860418, 1140860426, 1140860454, 1140860470, 1140860478, 1140860562, 1140860610, 1140860690, 1140860696, 1140860714, 1140860728, 1140860750, 1140860764, 1140860790, 1140860802, 1140860806, 1140860882, 1140860904, 1140860972, 1140861088, 1140861090, 1140861128, 1140861138, 1140861190, 1140861276, 1140864950, 1140864952, 1140866078, 1140866122, 1140866128, 1140866136, 1140866236, 1140866248, 1140866446, 1140866450, 1140866738, 1140866756, 1140866800, 1140871986, 1140875934, 1140879760, 1140879762, 1140879778, 1140879798, 1140879802, 1140879806, 1140879818, 1140879842, 1140879854, 1140881702, 1140881728, 1140883468, 1140888510, 1140888552, 1140888556, 1140888560, 1140888646, 1140909368, 1140909708, 1140910442, 1140910512, 1140910606, 1140916356, 1140916362, 1140917428, 1140923336, 1140923572, 1140923788, 1140926778, 1140926780, 1140928284, 1141145660, 1141145668, 1141146124, 1141146126, 1141146128, 1141151016, 1141151018, 1141152998, 1141153026, 1141153328, 1141156836, 1141164276, 1141165470, 1141166006, 1141171152, 1141171336, 1141172492, 1141172682, 1141172698, 1141173888, 1141179974, 1141180592, 1141180598, 1141180778, 1141187788, 1141187790, 1141188790, 1141190160, 1141193282, 1141194794, 1141194800, 1141194804, 1141194808, 1141194810, 1141195254, 1141195258, 1141201038, 1141201040 | FID 6153 (Medication for cholesterol, blood pressure, diabetes, or take exogenous hormones) = 'Blood pressure medication' or  FID 6177 (Medication for cholesterol, blood pressure or diabetes) = 'Blood pressure medication' |
|  | Statin use | Statin use; In administrative data they used ATC code C10AA, C10B |  |  | 1140861958, 1140869130, 1140869132, 1140869196, 1140870208, 1140873350, 1140873570, 1140874030, 1140874266, 1140874360, 1140878594, 1140878598, 1140880388, 1140880390, 1140882794, 1140882806, 1140882844, 1140882938, 1140883060, 1140884216, 1140888594, 1140888648, 1140910632, 1140910654, 1141146234, 1141157400, 1141192410 | FID 6153 (Medication for cholesterol, blood pressure, diabetes, or take exogenous hormones) = 'Cholesterol lowering medication' or FID 6177 (Medication for cholesterol, blood pressure or diabetes) = 'Cholesterol lowering medication' |
|  | eGFR | mL/min per 1.73 m2 |  |  |  | Calculated with FID 21022 (Age at recruitment), FID 31 (Sex), FID 30720 (Cystatin C) & FID 23478 (Creatinine) using the formula from https://www.kidney.org/ckd-epi-creatinine-cystatin-equation-2021 |
| SCORE2 | Age | Years |  |  |  | 21022- Age at recruitment |
|  | Smoking | 1= Current  0= Previous/Never |  |  |  | FID 20116 (Smoking Status) 1 = Current  0 = Previous, Never, Prefer not to answer |
|  | Diabetes*^ | Yes/no | 1220  1221  1222  1223 | E10-14  O24 | 1140868902, 1140874646, 1140874744, 1140883066, 1140884600, 1140909780, 1141152590, 1141157284, 1141168660, 1141171646, 1141177600, 1141180722, 1141180734, 1141189090 | FID 2976 (Age diabetes diagnosed) is smaller than FID 21022 (Age at recruitment), FID 4041 (Gestational diabetes only) = 'Yes', FID 2443 (Diabetes diagnosed by doctor) = 'Yes', FID 30750 (HbA1c) >= 48, FID 30740 (Glucose) >= 11.1, FID 6153 (Medication for cholesterol, blood pressure, diabetes, or take exogenous hormones) = 'Insulin’, FID 6177 (Medication for cholesterol, blood pressure or diabetes) = 'Insulin’ |
|  | SBP | mmHg |  |  |  | Mean of all measurements found in FID 4080 (automated reading) and FID 93 (manual reading). Two measures of blood pressure were taken a few moments apart. |
|  | Total cholesterol | mmol/L |  |  |  | FID 30690 – Cholesterol mmol/L |
|  | HDL cholesterol | mmol/L |  |  |  | FID 23406 -HDL Cholesterol mmol/L |
|  | Age at diabetes diagnosis^ | Age at diabetes diagnosis |  |  |  | FID 2976- Age at diabetes diagnosis |
|  | HbA1c^ | HbA1c |  |  |  | FID 30750- HbA1c |
|  | eGFR^ | Creatinine-based eGFR |  |  |  | Calculated with FID 21022 (Age at recruitment), FID 31 (Sex), FID 30720 (Cystatin C) & FID 23478 (Creatinine) using the formula from https://www.kidney.org/ckd-epi-creatinine-cystatin-equation-2021 |

- Variables denoted with * were used in SCORE2-OP
- Variables denoted with ^ were used in SCORE2- Diabetes

**Table SΑ2a.** SCORE-2 Outcome Definition and ICD 10 codes

|  | **Outcome** | **ICD-10 Code** | **UKBB** |
| --- | --- | --- | --- |
| Fatal CVD event from one of the following | Hypertensive disease | I10-16 | I10-16 |
|  | Ischemic heart disease | I20-25 | I20-25 |
|  | Arrhythmias, heart failure | I46-52 | I46-52 |
|  | Cerebrovascular disease | I60-69 | I60-69 |
|  | Atherosclerosis and peripheral vascular disease/AAA | I70-73 | I70-73 |
|  | Sudden death | R96-96.1 | R96-96.1 |
| Non-Fatal CVD event | Non-fatal myocardial infarction | I21-23 | I21-23 |
|  | Non-fatal stroke | I60-69 | I60-69 |

**Table SΑ2b.** PREVENT Outcome Definition and ICD 10 codes

The primary outcome was incident total CVD, which was defined as a composite of fatal and nonfatal ASCVD and HF events.- ASCVD included coronary heart disease (CHD: myocardial infarction and fatal CHD) and stroke as a composite outcome.

### ICD codes used to define outcome if not specified

| **Outcome** | **ICD-10 codes** | **UKBB** |
| --- | --- | --- |
| Myocardial infarction (MI) | I21, I22 | I21, I22 |
| Hemorrhagic and Ischemic stroke | I61, I62, I63 | I61, I62, I63 |
| Heart failure | I50 | I50 |

**Table SΑ2c.** QRISK3 Outcome Definition and ICD 10 codes

Outcome was cardiovascular disease, which was defined as a composite outcome of coronary heart disease, ischaemic stroke, or transient ischaemic attack.

| **Outcome** | **ICD-10 codes** | **UKBB** |
| --- | --- | --- |
| Ischemic Heart Disease | I20-25, R07.2, Z86.74 | I20-I25 |
| Stroke | I63-I66, + G45.X | I63-I66, G45.X |

Table S2d Read codes used to identify patients with cardiovascular disease from GP records.

| **Outcome** | **ICD-10 Code** |
| --- | --- |
| Ischaemic heart disease | I20-25 |
| Arteriosclerotic heart disease | I25.1 |
| Atherosclerotic heart disease | I25.1 |
| IHD - Ischaemic heart disease | I25.1 |
| Acute myocardial infarction | I21.9 |
| Attack - heart | I21.9 |
| Coronary thrombosis | I21.0 |
| Cardiac rupture following myocardial infarction (MI) | I23.1 |
| Heart attack | I21.9 |
| MI - acute myocardial infarction | I21.9 |
| Thrombosis - coronary | I21.0 |
| Silent myocardial infarction | I24.8 |
| Coronary thrombosis | I21.0 |
| Myocardial Infarction | I21.9 |
| Acute anterolateral infarction | I21.0 |
| Other specified anterior myocardial infarction | I21.1 |
| Acute anteroapical infarction | I21.1 |
| Acute anteroseptal infarction | I21.1 |
| Anterior myocardial infarction NOS | I21.1 |
| Acute inferolateral infarction | I21.2 |
| Acute inferoposterior infarction | I21.2 |
| Posterior myocardial infarction NOS | I21.3 |
| Lateral myocardial infarction NOS | I21.4 |
| True posterior myocardial infarction | I21.3 |
| Acute subendocardial infarction | I21.4 |
| Acute non-Q wave infarction | I21.4 |
| Acute non-ST segment elevation myocardial infarction | I21.4 |
| Inferior myocardial infarction NOS | I21.2 |
| Acute Q-wave infarct | I21.0-I21.3 |
| Mural thrombosis | I23.6 |
| Acute posterolateral myocardial infarction | I21.2 |
| Acute transmural myocardial infarction of unspecif site | I21.9 |
| Acute ST segment elevation myocardial infarction | I21.0-I21.3 |
| Other acute myocardial infarction | I21.8 |
| Acute atrial infarction | I21.8 |
| Acute papillary muscle infarction | I21.8 |
| Acute septal infarction | I21.1 |
| Other acute myocardial infarction NOS | I21.8 |
| Acute myocardial infarction NOS | I21.9 |
| Other acute and subacute ischaemic heart disease | I24.8 |
| Acute/subacute IHD NOS | I24.8 |
| Postmyocardial infarction syndrome | I24.1 |
| Dressler's syndrome | I24.1 |
| Preinfarction syndrome | I20.0 |
| Crescendo angina | I20.0 |
| Impending infarction | I20.0 |
| Unstable angina | I20.0 |
| Angina at rest | I20.0 |
| Myocardial infarction aborted | I20.0 |
| MI - myocardial infarction aborted | I20.0 |
| Unstable angina | I20.0 |
| Angina at rest | I20.0 |
| Refractory angina | I20.0 |
| Worsening angina | I20.0 |
| Acute coronary syndrome | I24.8 |
| Preinfarction syndrome NOS | I20.0 |
| Coronary thrombosis not resulting in myocardial infarction | I24.0 |
| Other acute and subacute ischaemic heart disease | I24.8 |
| Acute coronary insufficiency | I24.8 |
| Acute coronary syndrome | I24.8 |
| Microinfarction of heart | I24.8 |
| Subendocardial ischaemia | I24.8 |
| Transient myocardial ischaemia | I24.8 |
| Other acute and subacute ischaemic heart disease NOS | I24.8 |
| Old myocardial infarction | I25.2 |
| Healed myocardial infarction | I25.2 |
| Personal history of myocardial infarction | Z07.74 |
| Angina pectoris | I20.9 |
| Angina decubitus | I20.8 |
| Nocturnal angina | I20.8 |
| Angina decubitus NOS | I20.8 |
| Prinzmetal's angina | I20.1 |
| Variant angina pectoris | I20.1 |
| Coronary artery spasm | I20.1 |
| Angina pectoris NOS | I20.9 |
| Status anginosus | I20.9 |
| Stenocardia | I20.9 |
| Syncope anginosa | I20.9 |
| Angina on effort | I20.9 |
| Ischaemic chest pain | R07.2 |
| Post infarct angina | I20 |
| New onset angina | I20.0 |
| Stable angina | I20.9 |
| Angina pectoris NOS | I20.9 |
| Other chronic ischaemic heart disease | I25.89 |
| Chr. ischaemic heart dis. NOS | I25.9 |
| Coronary Heart Disease | I25.10 |
| Coronary atherosclerosis | I25.10 |
| Triple vessel disease of the heart | I25.118 |
| Coronary artery disease | I25.10 |
| Single coronary vessel disease | I25.111 |
| Double coronary vessel disease | I25.112 |
| Atherosclerotic cardiovascular disease | I25.10 |
| Ischaemic cardiomyopathy | I25.5 |
| Silent myocardial ischaemia | I20.9 |
| Other specified chronic ischaemic heart disease | I25.89 |
| Chronic coronary insufficiency | I25.9 |
| Chronic myocardial ischaemia | I25.9 |
| Other specified chronic ischaemic heart disease NOS | I25.9 |
| Other chronic ischaemic heart disease NOS | I25.9 |
| Asymptomatic coronary heart disease | I25.9 |
| Subsequent myocardial infarction | I22.x |
| Subsequent myocardial infarction of anterior wall | I22.0 |
| Subsequent myocardial infarction of inferior wall | I22.1 |
| Subsequent myocardial infarction of other sites | I22.8 |
| Subsequent myocardial infarction of unspecified site | I22.9 1 |
| Certain current complication follow acute myocardial infarct | I23.x |
| Haemopericardium/current comp folow acut myocard infarct | I23.0 |
| Atrial septal defect/curr comp folow acut myocardal infarct | I23.1 |
| Ventric septal defect/curr comp fol acut myocardal infarctn | I23.2 |
| Ruptur cardiac wall w'out haemopericard/cur comp fol ac MI | I23.3 |
| Ruptur chordae tendinae/curr comp fol acute myocard infarct | I23.4 |
| Rupture papillary muscle/curr comp fol acute myocard infarct | I23.5 |
| Thrombosis atrium,auric append&vent/curr comp foll acute MI | I23.6 |
| Other specified ischaemic heart disease | I25.89 |
| Ischaemic heart disease NOS | I25.9 |
| Post infarction pericarditis | I24.1 |
| [X]Acute transmural myocardial infarction of unspecif site | I21.3 |
| Stroke or TIA | I63.x or G45.x |
| Amaurosis fugax | G45.3 |
| Stroke or TIA | I63.x or G45.x |
| [X]Other transnt cerebral ischaemic attacks+related syndroms | G45.9 |
| Cerebral infarct due to thrombosis of precerebral arteries | I63.2 |
| Cerebral infarction due to embolism of precerebral arteries | I63.1 |
| Cerebral arterial occlusion | I63-I66 |
| CVA - cerebral artery occlusion | I66.x |
| Infarction - cerebral | I63.x |
| Stroke due to cerebral arterial occlusion | I66.x |
| Cerebral thrombosis | I63.31 |
| Cerebral infarction due to thrombosis of cerebral arteries | I63.31 |
| Cerebral embolism | I63.32 |
| Cerebral embolus | I63.32 |
| Cerebral infarction due to embolism of cerebral arteries | I63.32 |
| Cerebral infarction NOS | I63.9 |
| Brainstem infarction NOS | I63.6 |
| Cerebellar infarction | I63.4 |
| Cerebral A. occlusion NOS | I66.9 |
| Brainstem infarction | I63.6 |
| Wallenberg syndrome | I63.6 |
| Lateral medullary syndrome | I63.6 |
| Left sided cerebral infarction | I63.3x (with laterality) |
| Right sided cerebral infarction | I63.3x (with laterality) |
| Infarction of basal ganglia | I63.3x (with laterality) |
| Transient cerebral ischaemia | G45.9 |
| Drop attack | G45.8 |
| Transient ischaemic attack | G45.9 |
| Vertebro-basilar insufficiency | G45.0 |
| Transient Ischaemic Attacks | G45.9 |
| Basilar artery syndrome | G45.0 |
| Insufficiency - basilar artery | G45.0 |
| Subclavian steal syndrome | G45.8 |
| Carotid artery syndrome hemispheric | G45.1 |
| Multiple and bilateral precerebral artery syndromes | G45.2 |
| Vertebrobasilar insufficiency | G45.0 |
| Other transient cerebral ischaemia | G45.8 |
| Transient cerebral ischaemia NOS | G45.9 |
| Transient Ischaemic Attacks | G45.9 |
| Impending cerebral ischaemia | G45.9 |
| Intermittent cerebral ischaemia | G45.9 |
| Transient cerebral ischaemia NOS | G45.9 |
| Stroke and cerebrovascular accident unspecified | I64 |
| CVA unspecified | I64 |
| Stroke unspecified | I64 |
| CVA - Cerebrovascular accident unspecified | I64 |
| Stroke/CVA - undefined | I64 |
| Stroke | I64 |
| Left sided CVA | I64 (with laterality) |
| Right sided CVA | I64 (with laterality) |
| Cereb infarct due cerebral venous thrombosis, nonpyogenic | I63.6 |
| Cereb infarct due unsp occlus/stenos precerebr arteries | I63.2 |

QRISK3 MODEL

| Characteristics | Derivation cohort | | Validation cohort | | UKBB | |
| --- | --- | --- | --- | --- | --- | --- |
|  | Women n=4,019,956 | Men n=3,869,847 | Women=  1,360,457 | Men=  1,310,841 | Women=  170,613 | Men=  132,057 |
| Mean (SD) age (years) | 43.3 (15.3) | 42.6 (14.0) | 43.3 (15.3) | 42.6 (13.8) | 55.5 (8.0) | 55.3 (8.2) |
| Mean (SD) Townsend score | 0.4 (3.2) | 0.5 (3.3) | 0.4 (3.3) | 0.5 (3.3) | -1.488 (3.0) | -1.423 (3.0) |
| Mean (SD) body mass index kg/m2 | 25.4 (5.1) | 25.9 (4.2) | 25.4 (5.1) | 25.9 (4.2) | 26.4 (4.3) | 27.2 (3.7) |
| Mean (SD) total cholesterol: HDL cholesterol ratio | 3.7 (1.2) | 4.4 (1.4) | 3.6 (1.2) | 4.4 (1.3) | 3.9 (1.0) | 4.6 (1.1) |
| Mean (SD) systolic blood pressure (mm Hg) | 123.2 (18.2) | 129.2 (16.3) | 123.1(18.1) | 128.8 (16.2) | 133.9 (18.6) | 140.0 (16.8) |
| Ethnic origin: n (%) |  |  |  |  |  |  |
| White or not recorded | 3,564,651 (88.7) | 3,435,408 (88.8) | 1,218,391 (89.6) | 1,171,281 (89.4) | 162,285 (95.12) | 125,282 (94.87) |
| Indian | 77,683  (1.9) | 81,805  (2.1) | 23,146  (1.7) | 26,479  (2.0) | 1,553 (0.91) | 1,377 (1.04) |
| Pakistani | 39,541  (1.0) | 46,948  (1.2) | 10,919  (0.8) | 14,787  (1.1) | 347 (0.20) | 501 (0.38) |
| Bangladeshi | 31,930  (0.8) | 42,111  (1.1) | 8738  (0.6) | 11,914  (0.9) | 25 (0.01) | 57 (0.04) |
| Other Asian | 53,559 (1.3) | 45,753 (1.2) | 17,078 (1.3) | 15,966 (1.2) | 460 (0.27) | 442 (0.33) |
| Black Caribbean | 37,781  (0.9) | 30,610  (0.8) | 13,142  (1.0) | 10,642  (0.8) | 1,416 (0.83) | 885 (0.67) |
| Black African | 77,813  (1.9) | 71,245  (1.8) | 27,678  (2.0) | 25,251  (1.9) | 703 (0.41) | 774 (0.59) |
| Chinese | 33,767  (0.8) | 23,730  (0.6) | 8992  (0.7) | 6098  (0.5) | 617 (0.36) | 368 (0.28) |
| Other | 103,231  (2.6) | 92,237  (2.4) | 32,373  (2.4) | 28,423  (2.2) | 3,207 (1.88) | 2,371 (1.80) |
| Smoking status: n (%) |  |  |  |  |  |  |
| Non-smoker | 2,051,803 (51.0) | 1,463,941 (37.8) | 706,671 (51.9) | 512,252 (39.1) | 104,218 (61.08) | 70,273 (53.21) |
| Former smoker | 589,521 (14.7) | 594,265 (15.4) | 194,545 (14.3) | 196,459 (15.0) | 52,271 (30.64) | 46,297 (35.06) |
| Light smoker | 434,954 (10.8) | 507,523 (13.1) | 154,565 (11.4) | 177,693 (13.6) | 4,926 (2.89) | 5,874 (4.45) |
| Moderate smoker | 226,128  (5.6) | 251,170  (6.5) | 74,933  (5.5) | 84,914  (6.5) | 3,006 (1.76) | 2,558 (1.94) |
| Heavy smoker | 115,890  (2.9) | 188,857  (4.9) | 38,218  (2.8) | 64,107  (4.9) | 6,192 (3.63) | 7,055 (5.34) |
| Medical characteristics: n (%) |  |  |  |  |  |  |
| Family history of coronary heart disease in first degree relative <60 years n (%) | 481,628 (12.0) | 357,987 (9.3) | 164,023 (12.1) | 123,039 (9.4) | 68,510 (40.2) | 44,800 (33.9) |
| Type 1 diabetes n (%) | 10,060  (0.3) | 11,617  (0.3) | 3351  (0.2) | 3932  (0.3) | 120 (0.07) | 117 (0.09) |
| Type 2 diabetes n (%) | 48,022  (1.2) | 58,395  (1.5) | 15,872  (1.2) | 19,318  (1.5) | 807 (0.5) | 1,278 (1.0) |
| Treated hypertension n (%) | 223,494  (5.6) | 164,255  (4.2) | 77,694  (5.7) | 56,920  (4.3) | 21,059 (12.3) | 16,329 (12.4) |
| Rheumatoid arthritis n (%) | 45,700  (1.1) | 20,997  (0.5) | 15,139  (1.1) | 7055  (0.5) | 6,069 (3.6) | 3.633 (2.8) |
| Atrial fibrillation n (%) | 15,177  (0.4) | 20,098  (0.5) | 5229  (0.4) | 6874  (0.5) | 903 (0.5) | 1,643 (1.2) |
| Chronic kidney disease (stage 4 or 5) n (%) | 7518  (0.2) | 6345  (0.2) | 2583  (0.2) | 2165  (0.2) | 2,310 (1.4) | 1,487 (1.1) |
| Chronic kidney disease (stage 3, 4, or 5) n (%) | 19,396 (0.5) | 12,254 (0.3) | 6949 (0.5) | 4232 (0.3) | NA | NA |
| Migraine n (%) | 257,825  (6.4) | 103,995  (2.7) | 89,504  (6.6) | 36,141  (2.8) | 23,647 (13.9) | 8,486 (6.4) |
| Corticosteroid use n (%) | 96,955  (2.4) | 56,533  (1.5) | 31,775  (2.3) | 18,634  (1.4) | 1,653 (1.0) | 1,172 (0.9) |
| HIV/AIDS n (%) | 4332  (0.1) | 7732  (0.2) | 1595  (0.1) | 2945  (0.2) | 34 (0.02) | 200 (0.2) |
| Systemic lupus erythematosus n (%) | 4010  (0.1) | 365  (0.0) | 1349  (0.1) | 134  (0.0) | 376 (0.2) | 40 (0.03) |
| Atypical antipsychotic use n (%) | 19,140  (0.5) | 20,123  (0.5) | 6268  (0.5) | 6597  (0.5) | 596 (0.3) | 407 (0.3) |
| Severe mental illness n (%) | 274,069  (6.8) | 167,115  (4.3) | 94,724  (7.0) | 57,830  (4.4) | 710 (0.4) | 641 (0.5) |
| Erectile dysfunction diagnosis or treatment n (%) | NA | 90,753  (2.3) | NA | 31,136  (2.4) | NA | 703 (0.5) |
| Erectile dysfunction diagnosis | NA | 80,753  (2.1) | NA | 27,727  (2.1) | NA | 329 (0.2) |
| Erectile dysfunction treatment | NA | 28,763  (0.7) | NA | 9877  (0.8) | NA | 417 (0.3) |
| Total CVD events n (%) | 160,549  (4) | 203,016  (5.2) | NA | NA | 7,230 (4.2) | 11,157 (8.4) |

PREVENT MODEL

| Characteristics | Derivation cohort | | Validation cohort | | UKBB | |
| --- | --- | --- | --- | --- | --- | --- |
|  | Women =  1,839,828 | Men =  1,44,091 | Women=  1,894,882 | Men=  1,435,203 | Women= 170,613 | Men=  132,057 |
| Mean (SD) age (years) | 53±13 | 52±12 | 52±13 | 52±12 | 55±8 | 55±8 |
| Mean (SD) body mass index (kg/m2) | 29±5 | 29±4 | 28±5 | 29±4 | 26±4 | 27±4 |
| Mean (SD) total cholesterol (mmol/L) | 5.0±0.8 | 4.9±0.8 | 5.0±0.8 | 4.9±0.8 | 5.9±1 | 5.8±1 |
| Mean (SD) systolic blood pressure (mm Hg) | 123±16 | 127±15 | 123±16 | 128±15 | 134±19 | 140±17 |
| Mean (SD) Non-high-density lipoprotein cholesterol (mmol/L) | 3.4±0.8 | 3.6±0.8 | 3.5±0.8 | 3.6±0.8 | 3.7±0.8 | 3.7±0.7 |
| Mean (SD ) High-density lipoprotein cholesterol (mmol/L) | 29±5 | 29±4 | 28±5 | 29±4 | 1.6±0.3 | 1.3±0.3 |
| Ethnic origin: % |  |  |  |  |  |  |
| White | 78 | 80 | 78 | 80 | 95.1 | 95 |
| Black | 10 | 8 | 10 | 8.2 | 1.3 | 1.3 |
| Hispanic | 6 | 5.3 | 4.2 | 3.7 | 0.8 | 0.8 |
| Asian | 2.6 | 2.5 | 2.7 | 2.2 | 1.8 | 2.1 |
| Other or missing | 4.1 | 4.6 | 4.9 | 5.5 | 1.0 | 1.0 |
| Smoking status: n (%) |  |  |  |  |  |  |
| Current smoker | 5.8 | 6.2 | 4.7 | 4.9 | 8.6 | 12.3 |
| Never smoker | NA | NA | NA | NA | NA | NA |
| Former smoker | NA | NA | NA | NA | NA | NA |
| Antihypertensive treatment n (%) | 23 | 27 | 24 | 29 | 12 | 12 |
| Statin treatment n (%) | 14 | 17 | 14 | 17 | 0 | 0 |
| Mean (SD) Estimated glomerular filtration rate, (mL/min per 1.73 m2) | 91±19 | 91±17 | 91±18 | 91±17 | 97±14 | 96±13 |
| Atherosclerotic cardiovascular disease events n (%) | 31,812 | 34,691 | 33,969 | 33,933 | 2,307 (1.4) | 4,378 (3.3) |
| Heart failure events n (%) | 30,957 | 28,393 | 30,287 | 25,679 | 1,078 (0.6) | 1,530 (1.2) |
| Total CVD events n (%) | 53,258 (2.8) | 53,403 (3.7) | 54,365 (2.8) | 50,489 (2.6) | 3,170 (1.9) | 5,476 (4.1) |
| Deaths n (%) | 84,289 | 80,897 | 82,555 | 76,783 | 175 (0.1) | 354 (0.3) |

SCORE2 MODEL

model and in the UKBB.

| Characteristics | Study | UKBiobank | |
| --- | --- | --- | --- |
|  |  | Prevent label | |
|  | 677,684 | Female=  170,613 | Male=  132,057 |
| Mean (SD) age (years) | 57 (9) | 55 (8) | 55 (8) |
| Male sex | 300,735 (44%) |  |  |
| Mean (SD) total cholesterol, (mmol/L) | 5.8 (1.1) | 5.9 (1.0) | 5.8 (1.0) |
| Mean (SD) HDL cholesterol (mmol/L) | 1.4 (0.4) | 1.6 (0.3) | 1.3 (0.3) |
| Mean (SD) systolic blood pressure (mm Hg) | 136 (19) | 134 (19) | 140 (17) |
| Smoking status: |  |  |  |
| Current smoker n (%) | 101,211 (15%) | 14,682 (91.4) | 16,301 (87.7) |
| Diabetes mellitus n (%) | 31,413 (4.6%) | 807 (0.5) | 1,278 (1.0) |
| Total CVD events n (%) | 30,121 (4.4%) | 2,647 (1.6) | 4,822 (3.7) |
| Follow-up (years, median (5^th^/95^th^ percentile) | 10.7 (5.0-18.6) | 10.0 (10.0-10.0) | 10.0 (6.9-10.0) |

In the descriptive statistics tables, the total number of CVD events, Atherosclerotic cardiovascular disease events (ASCVD) events, heart failure events, deaths, and follow-up times (where they exist) were reported according to the outcome definition specific to each model (e.g., QRISK3 model – QRISK3 outcome definition). In contrast, Table 2 focuses on the rate of events when each algorithm is applied to different outcome definitions in turn.

**Table SΑ4**. Proportions of missing observations for each predictor variable by sex, among the required input variables for each model.

| Missing values (%) | Females | Males |
| --- | --- | --- |
| *Systolic blood pressure* | 0.28 | 0.24 |
| *Total cholesterol* | 6.86 | 6.18 |
| *HDL cholesterol* | 15.36 | 13.37 |
| *Total/HDL cholesterol ratio* | 15.38 | 13.39 |
| *Body Mass Index* | 0.53 | 0.72 |
| *Townsend Deprivation Index* | 0.12 | 0.13 |
| *Standard deviation of systolic blood pressure* | 0.43 | 0.29 |
| *Weight* | 0.50 | 0.62 |
| *Height* | 0.42 | 0.60 |
| *eGFR* | 6.97 | 6.30 |
| *Glycated Haemoglobin HbA1c* | 7.49 | 6.78 |
| *Age at diabetes diagnosed* | 96.73 | 93.17 |

**Table SΑ5a**. Discrimination performance of SCORE2 models using coefficients derived by excluding the UKBB from the developing cohorts.

| ***Outcome definition*** | ***N*** | | **SCORE2 Equations** | | | |
| --- | --- | --- | --- | --- | --- | --- |
|  |  |  | ***AUC***  ***(95% CI)*** | | ***Brier Score*** | |
|  |  |  |  |  | ***Overall*** | |
|  | *Females* | *Males* | *Females* | *Males* | *Females* | *Males* |
| *PREVENT* | 170,613 | 132,057 | 0.7352 (0.7350-0.7355) | 0.6842 (0.6841-0.6843) | 0.0169 | 0.0360 |
| *SCORE2* |  |  | 0.7312 (0.7309-0.7315) | 0.6847 (0.6846-0.6848) | 0.0141 | 0.0318 |
| *QRISK3* |  |  | 0.7074 (0.7072-0.7076) | 0.6800 (0.6799-0.6801) | 0.0362 | 0.0681 |

**Table SΑ5b**. Calibration slope and intercept of SCORE2 models using coefficients derived by excluding the UKBB from the developing cohorts.

| **Mean (95% CI)** | | | | | | | |
| --- | --- | --- | --- | --- | --- | --- | --- |
| **Algorithm** | **Outcome definition** | **PREVENT** | | **SCORE2** | | **QRISK3** | |
|  |  | **Women** | **Men** | **Women** | **Men** | **Women** | **Men** |
| **SCORE2 Equations** | | | | | | | |
| *Calibration Slope* | | 0.487  (0.381‒0.593) | 0.328  (0.244‒0.412) | 0.441  (0.344‒0.538) | 0.306  (0.225‒0.386) | 0.749  (0.572‒0.927) | 0.541  (0.395‒0.687) |
| *Calibration Intercept* | | 0.014  (0.000‒0.027) | 0.036  (0.021‒0.052) | 0.011  (-0.001‒0.023) | 0.032  (0.018‒0.047) | 0.032  (0.010‒0.055) | 0.071  (0.044‒0.097) |

**Figure S1a**. Flowchart of participants included based on all model eligibility criteria

**
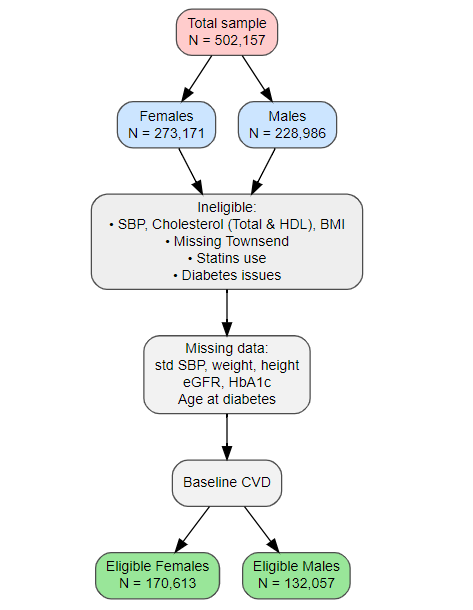
**
