# Supplementary material for: Independent external validation and head-to-head comparison of guideline-recommended CVD risk prediction models
Source: Am J Prev Cardiol. 2026 Apr 11;28:101625. doi: 10.1016/j.ajpc.2026.101625 (PMC13326136; doi:10.1016/j.ajpc.2026.101625)
Supplement: Supplementary file 3 [file mmc3.docx]

PREVENT outcome definition: Better accuracy for the Female population, poor visual calibration overall since the risk is overestimated in both groups.

Female Male


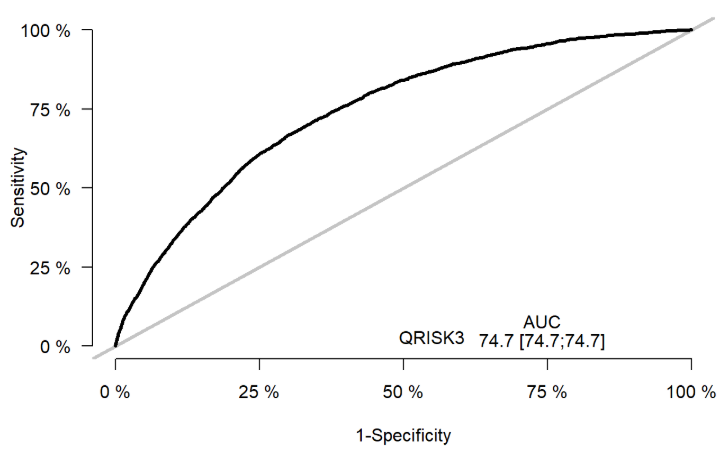

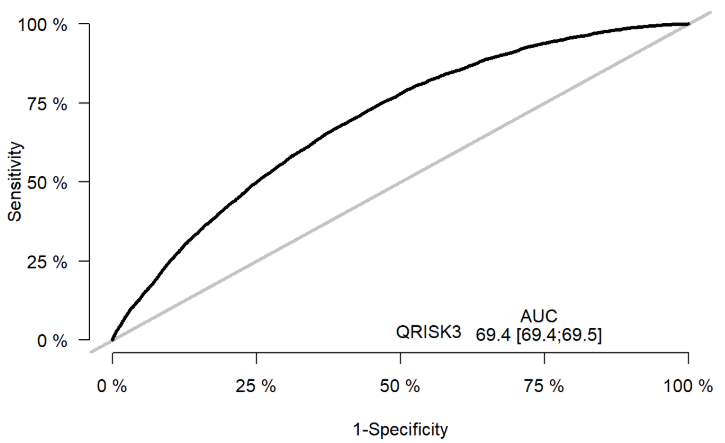


Female Male
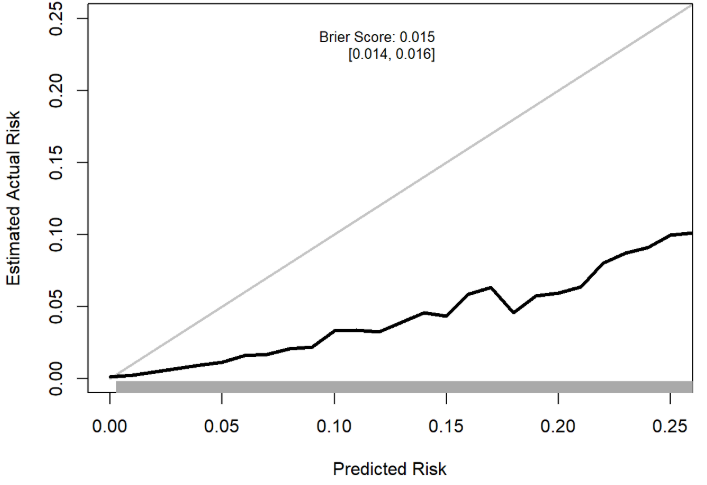

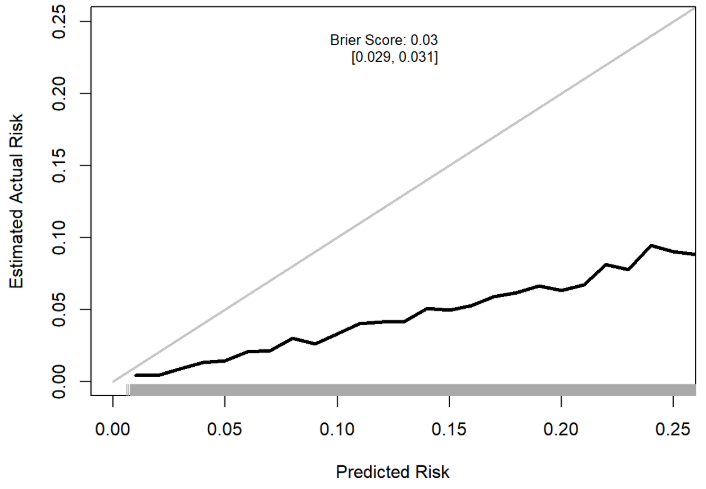


Calibration curve by age: Poor visual calibration as age increases. The risk is overestimated for both groups

Female Male
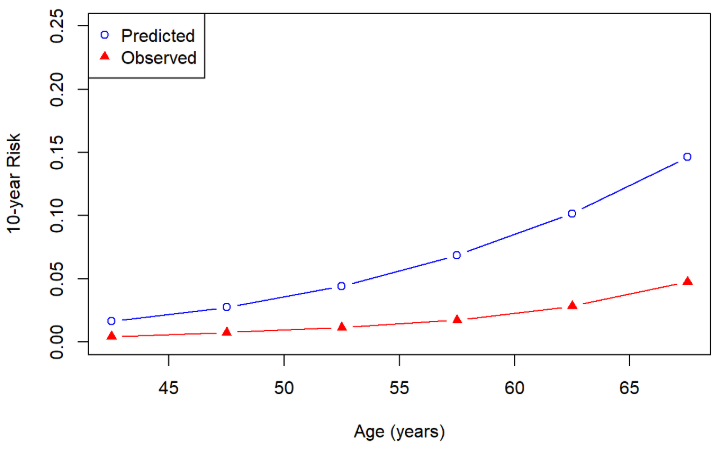

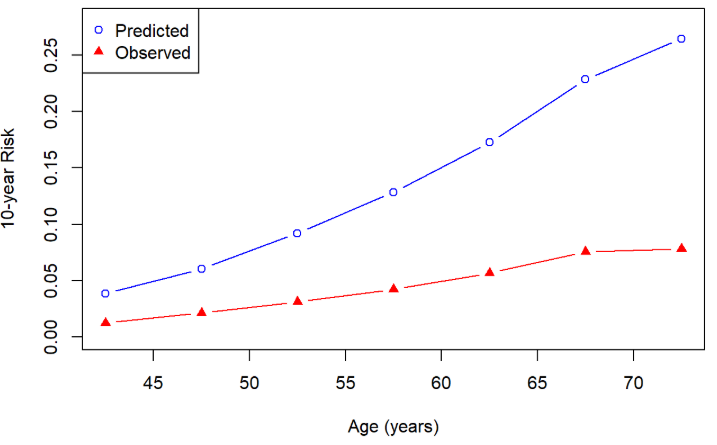


Calibration curve by SBP: Poor calibration for both groups with increasing SBP, there is risk of overestimation overall.

Female Male
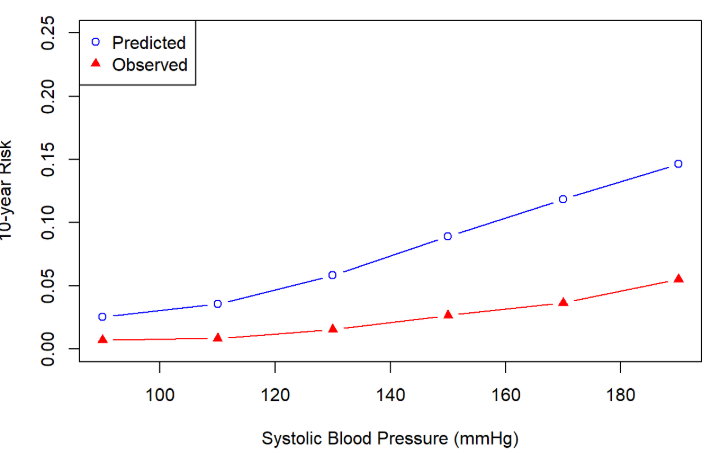

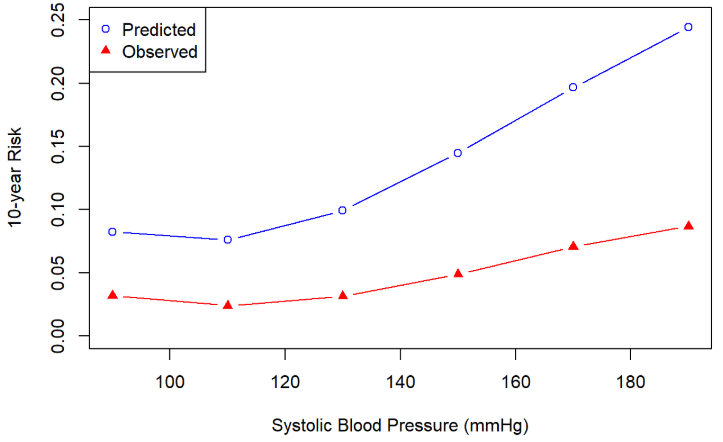


Decile-based calibration plot: Poor calibration. Risk is consistently overestimated, especially at higher probabilities.

Female Male


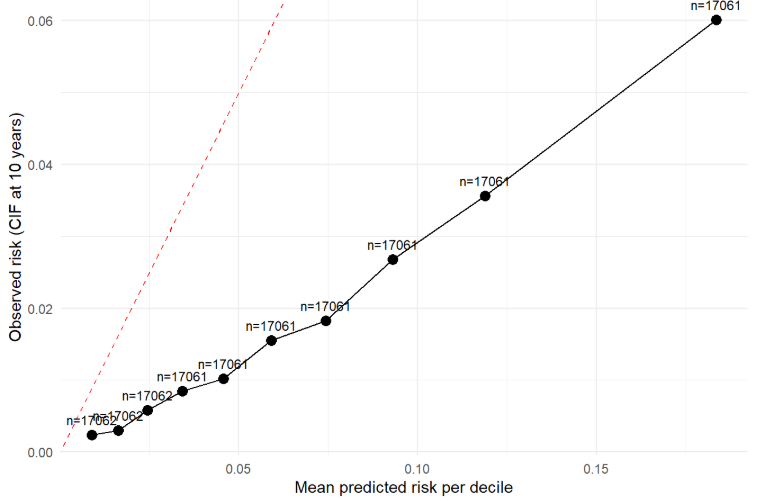

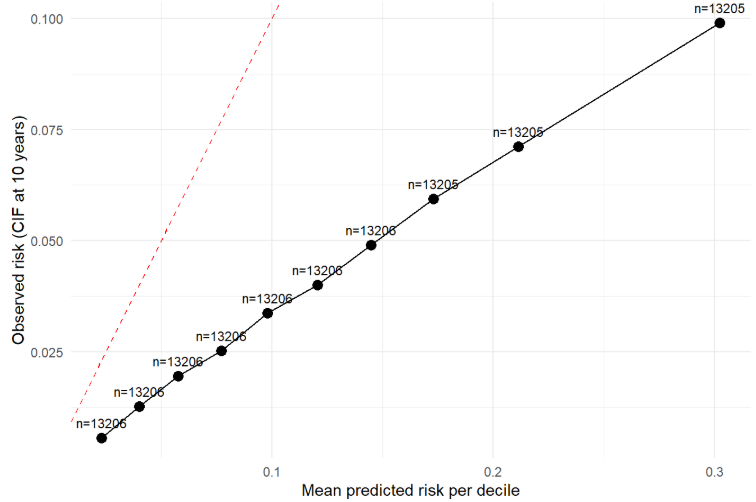


The QRISK3 model is rather poorly calibrated for the PREVENT outcome definition using the UKBB data.

SCORE2 outcome definition: Better accuracy for the Female population, poor visual calibration overall since the risk is overestimated in both groups.

Female Male


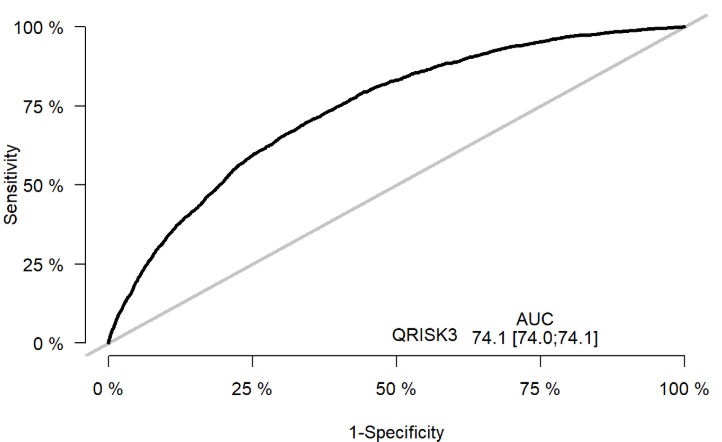

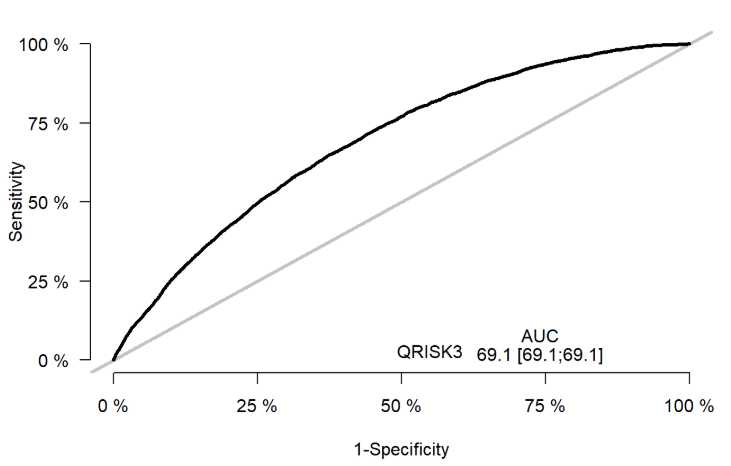


Female Male


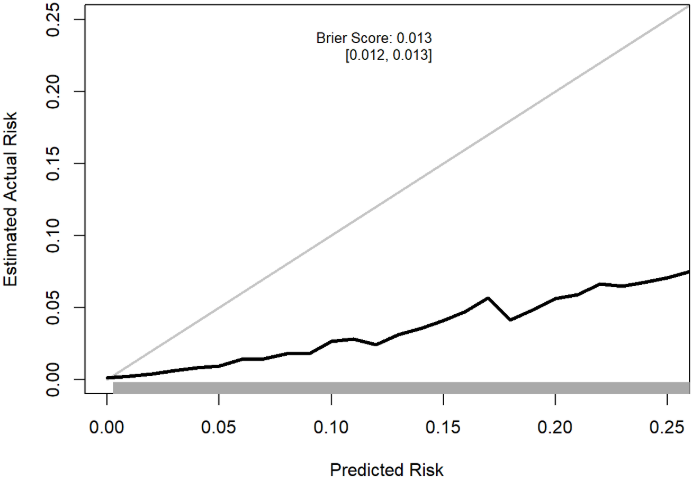

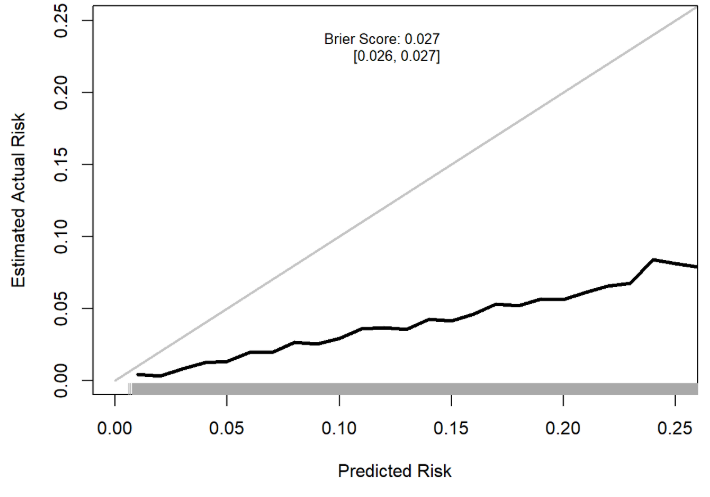


Calibration curve by age: Poor visual calibration as age increases. The risk is overestimated for both groups

Female Male
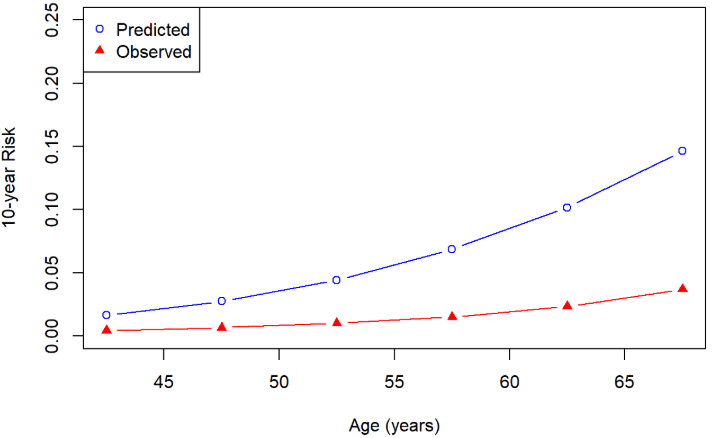

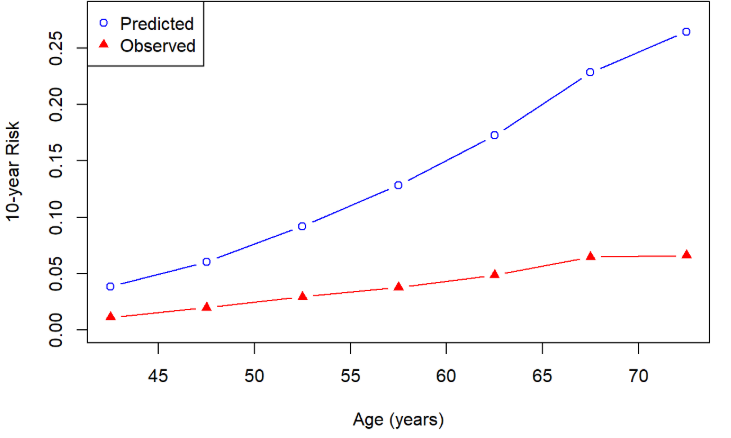


Calibration curve by SBP: Poor calibration for both groups with increasing SBP, there is risk of overestimation overall.

Female Male


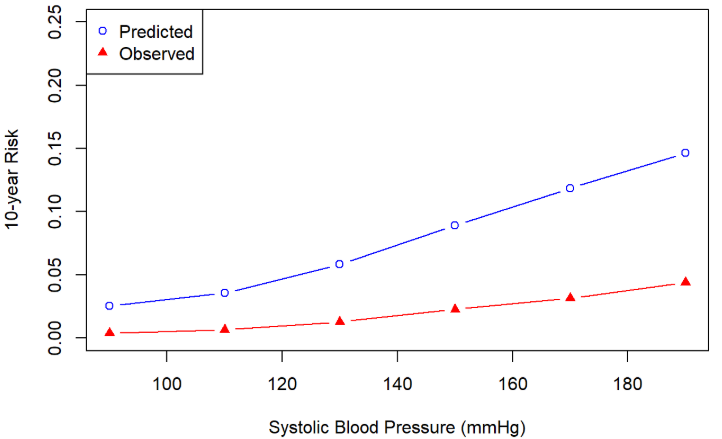

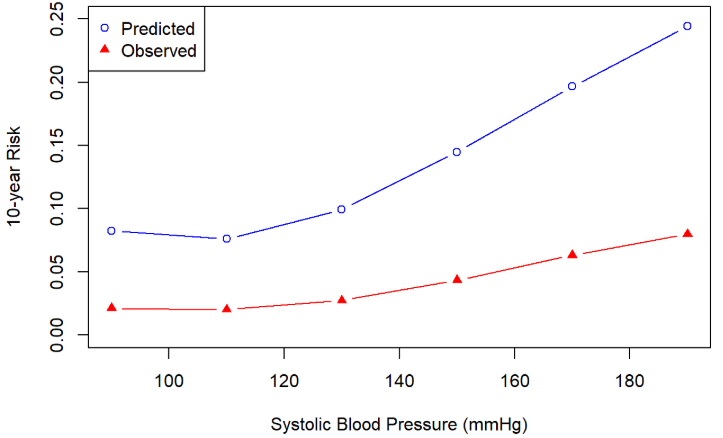


Decile-based calibration plot: Poor calibration. Risk is consistently overestimated, especially at higher probabilities.

Female Male


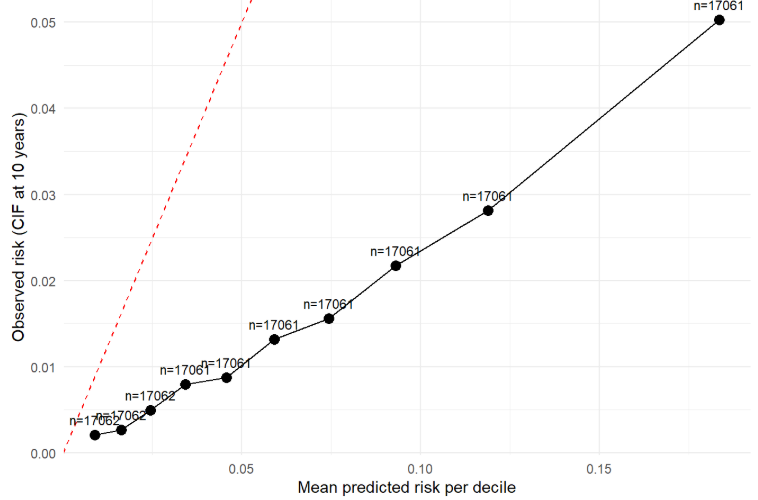

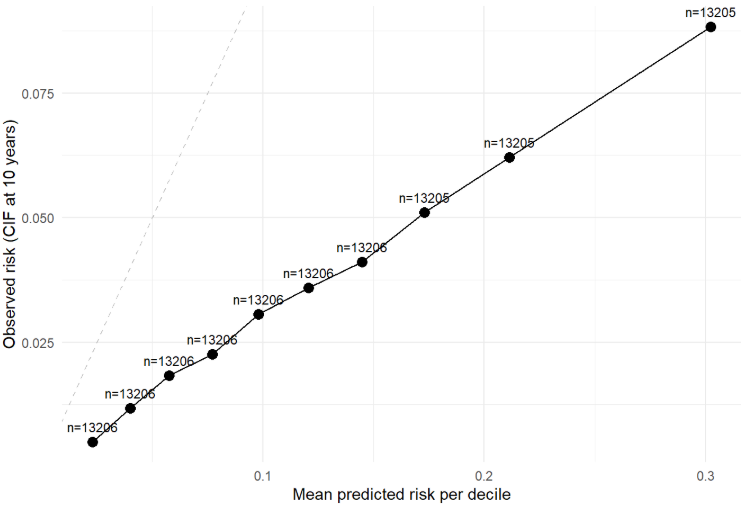


The QRISK3 model is poorly calibrated for the SCORE2 outcome definition using the UKBB data.

QRISK3 outcome definition: better accuracy for the Female population with mediocre visual calibration for both groups, some overestimation of risks exists.

Female Male


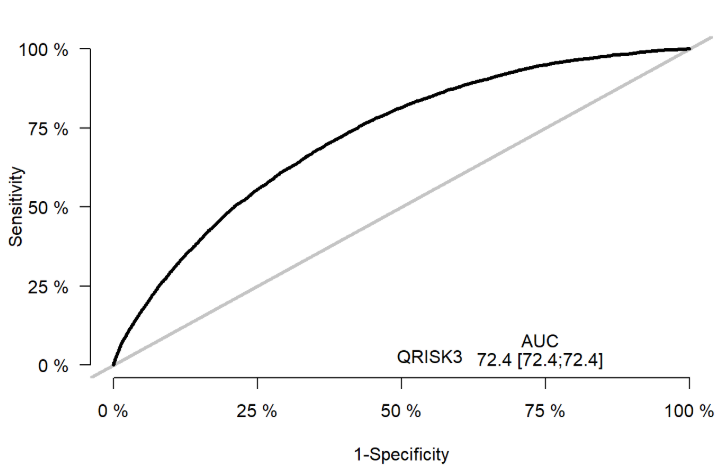

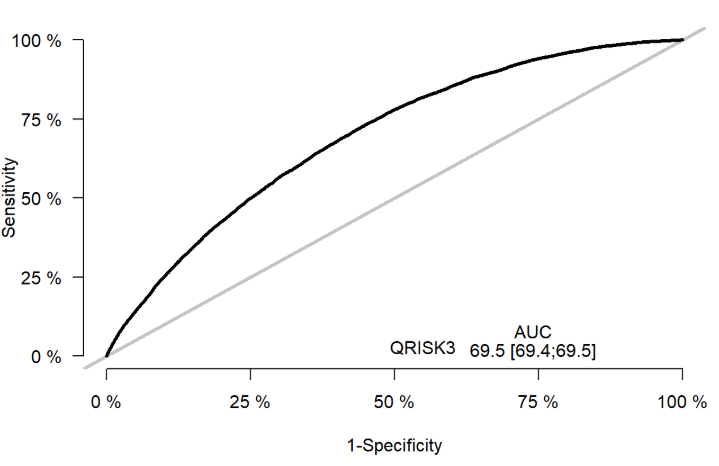


Female Male


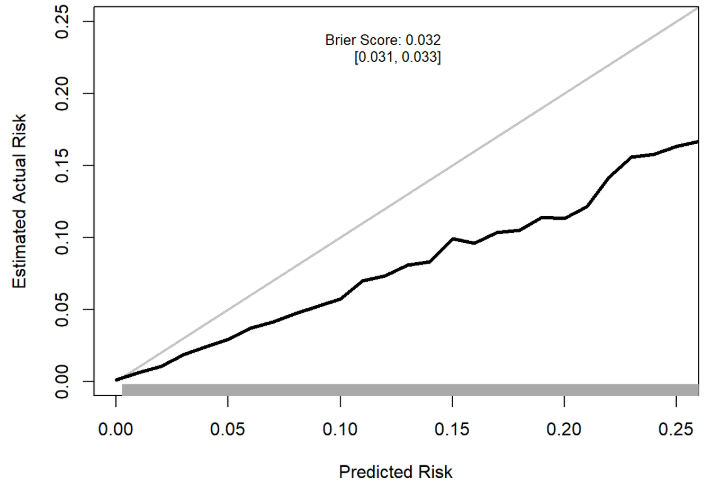

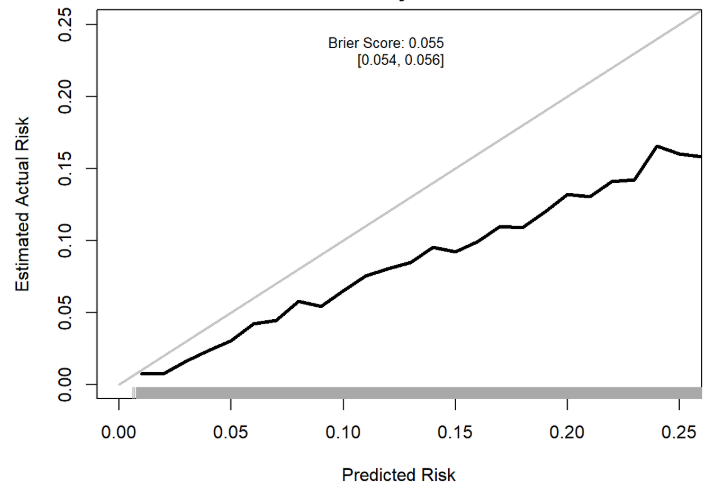


Calibration curve by age: mediocre calibration for both groups, especially with increasing age.

Female Male


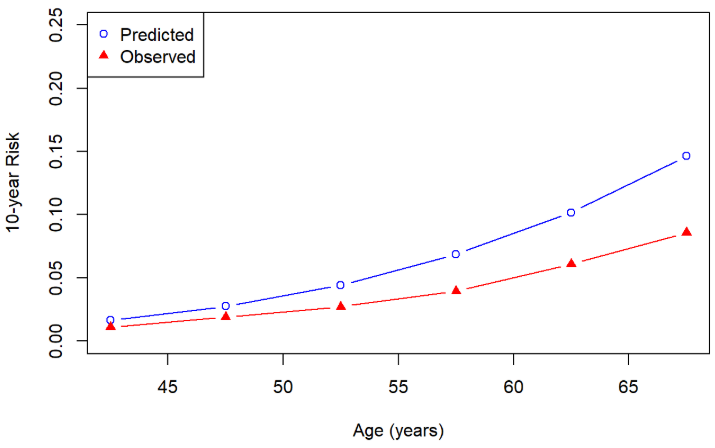

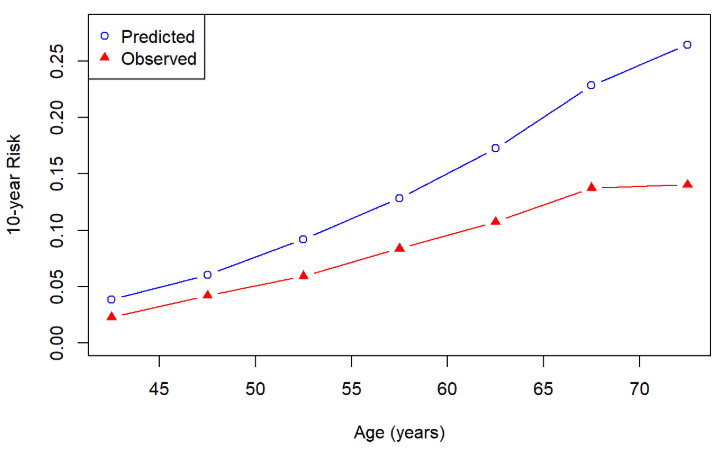


Calibration curve by SBP: mediocre calibration for high values, especially for the male population.

Female Male


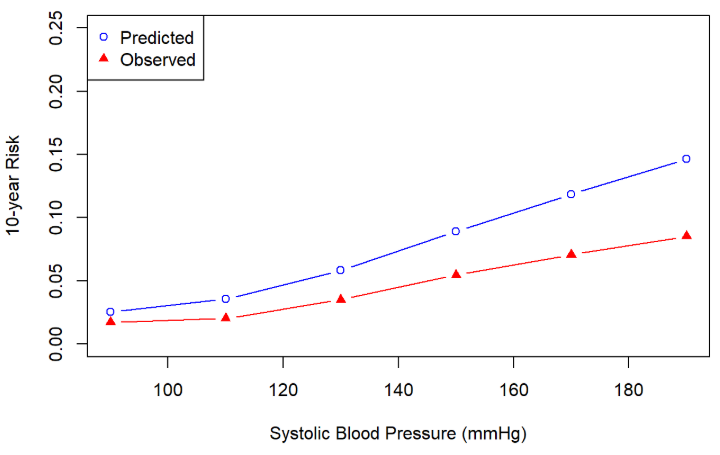

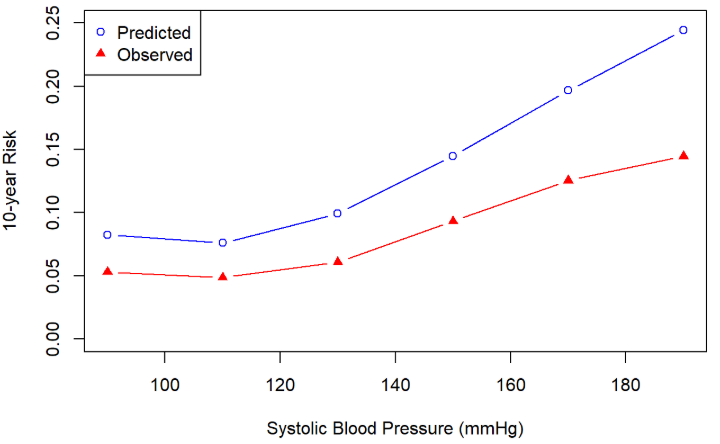


Decile-based calibration plot: Mediocre calibration. Risk is overestimated, especially at higher probabilities.

Female Male


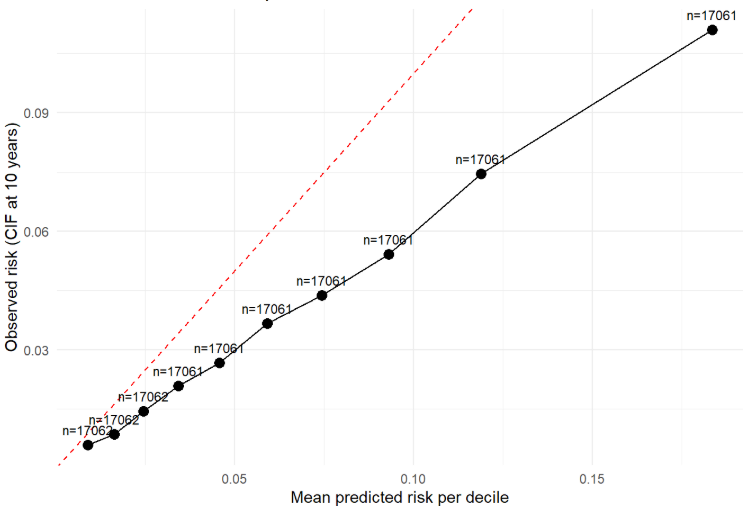

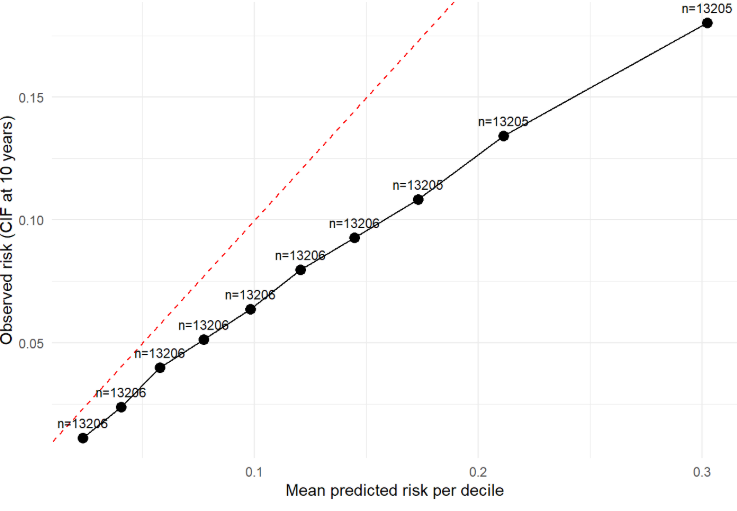


The QRISK3 model shows mediocre calibrations for the QRISK3 outcome definition using the UKBB data

PREVENT outcome definition: better accuracy for the Female population with mediocre visual calibration for both groups, some overestimation of risks exists.

Female Male


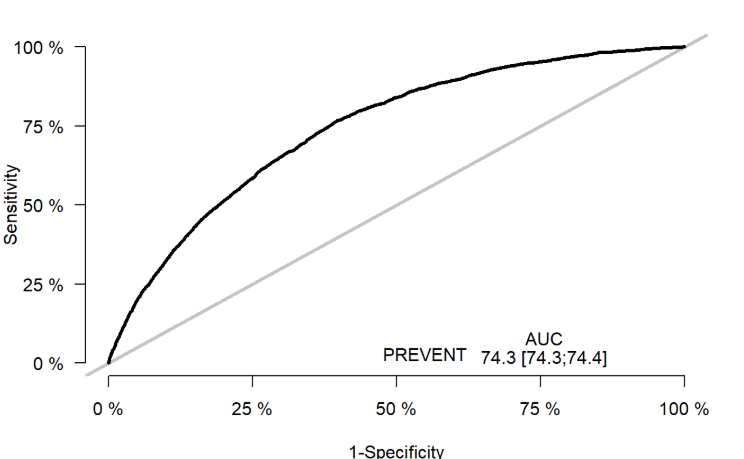

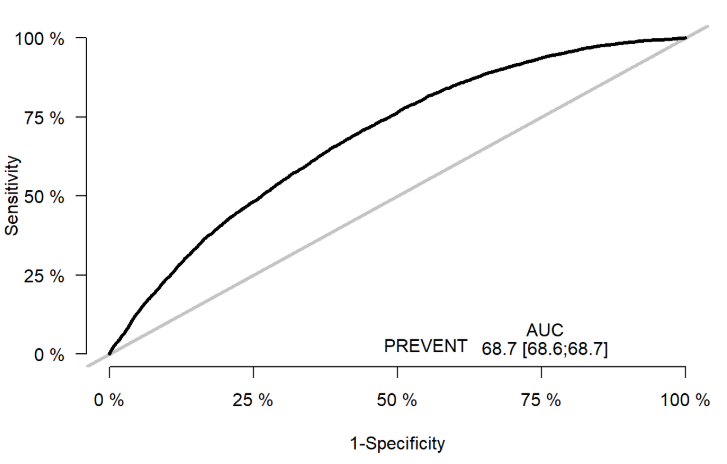


Female Male


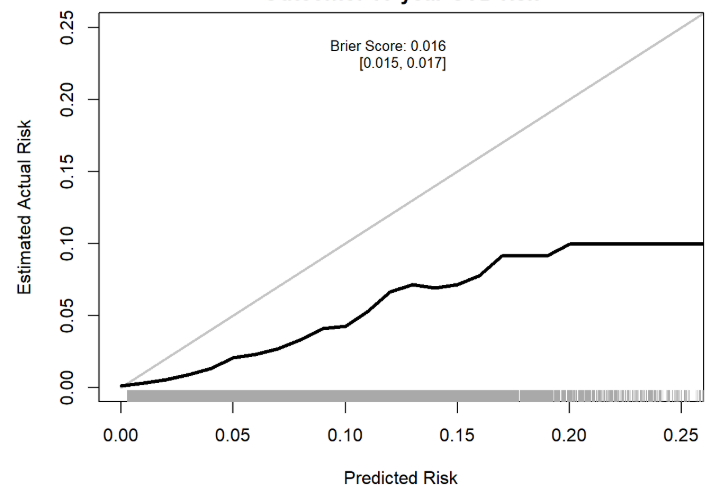

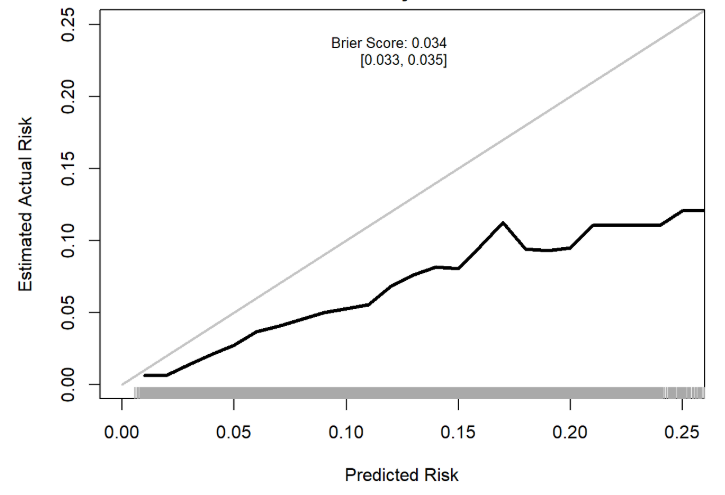


Calibration curve by age: mediocre calibration, for both groups, overestimated risks for higher ages exist.

Female Male


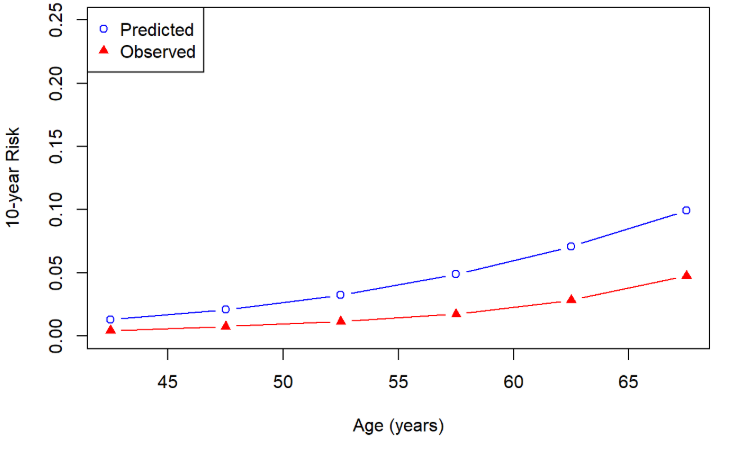

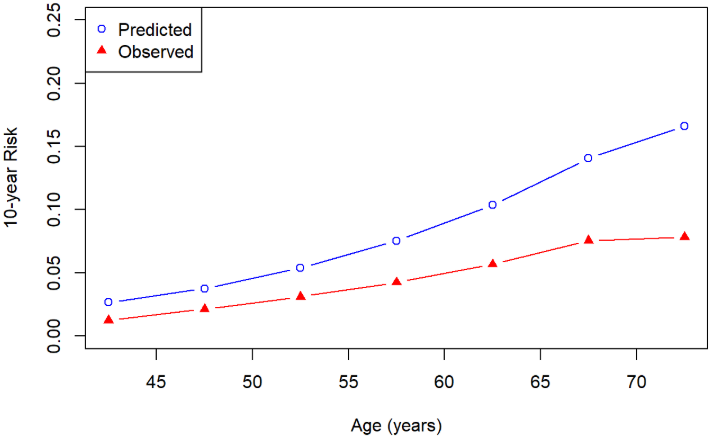


Calibration curve by SBP: mediocre calibration for both groups, with somewhat overestimated risks for higher values.

Female Male


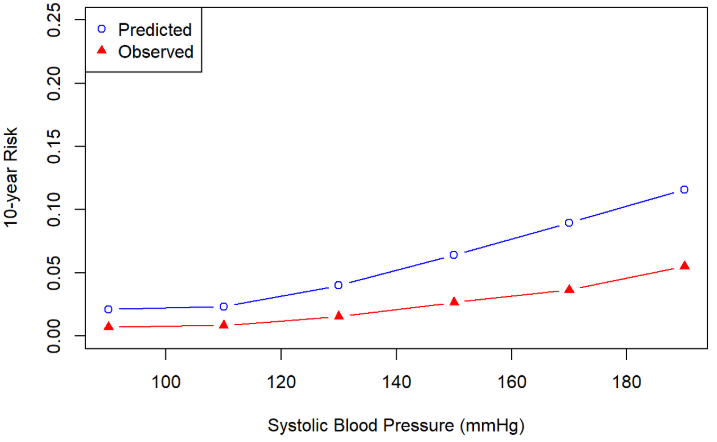

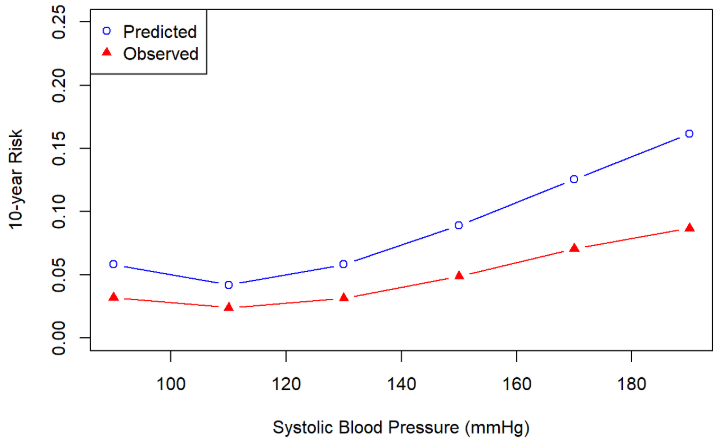


Decile-based calibration plot: Poor calibration. Risk is consistently overestimated, especially at higher probabilities.

Female Male


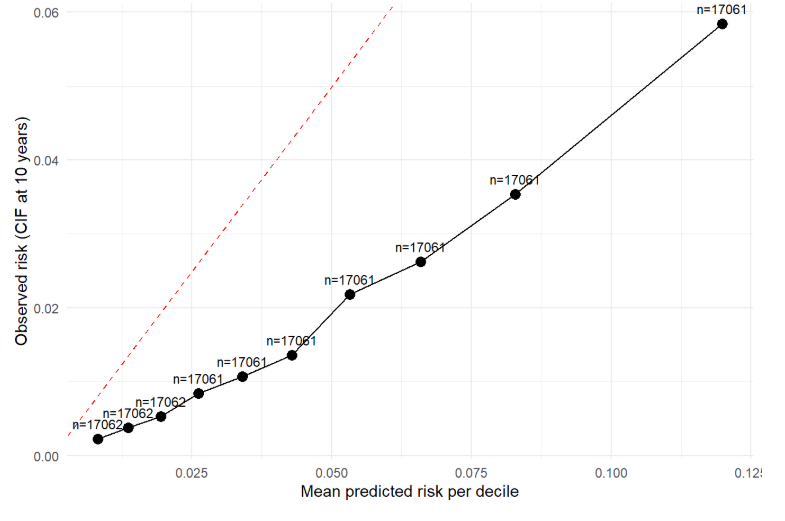

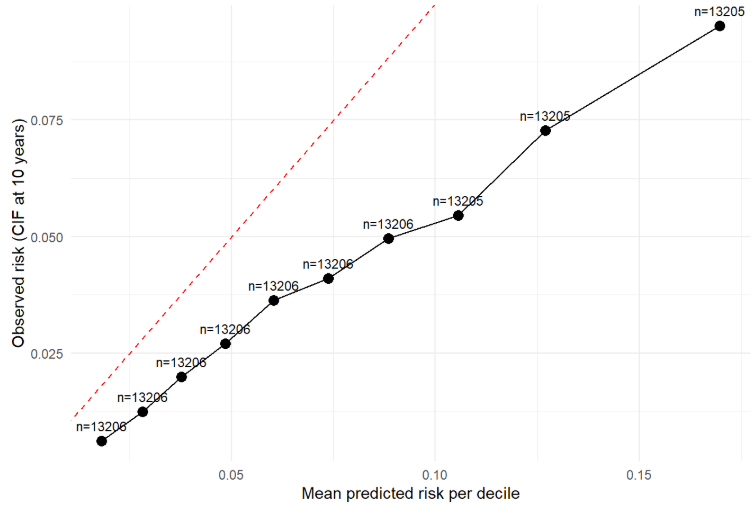


The PREVENT model has limited usefulness using the PREVENT outcome definition for the UKBB data.

SCORE2 Outcome definition: Better accuracy for the Female population, poor visual calibration overall since the risk is overestimated in both groups

Female Male


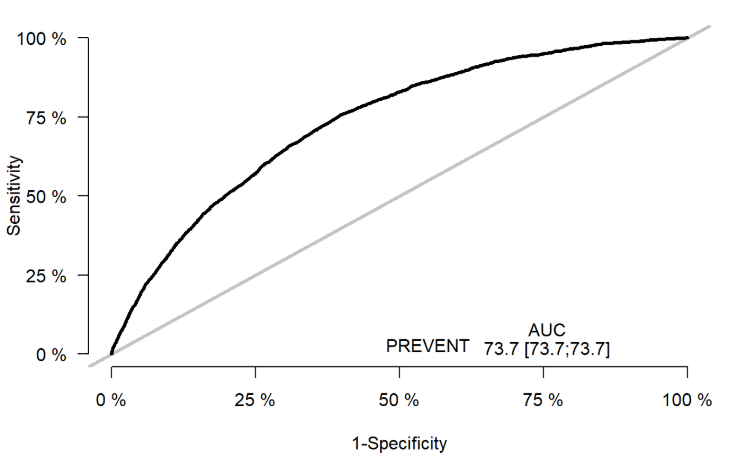

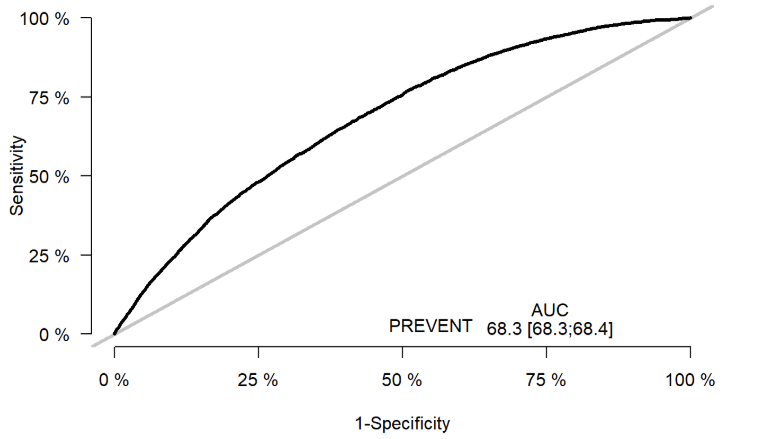


Female Male


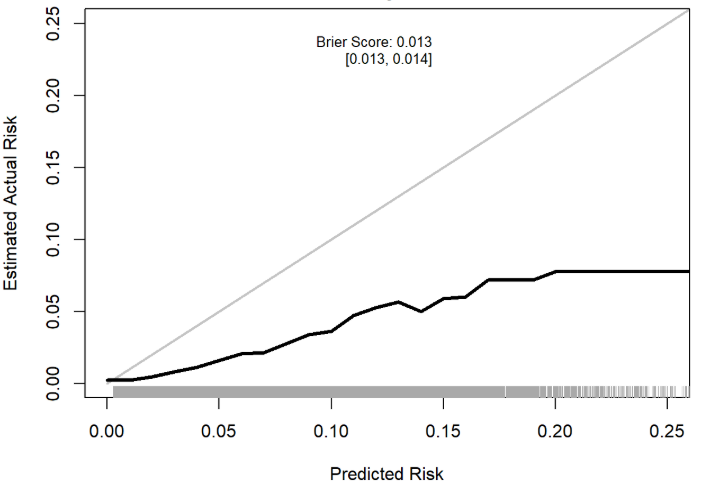

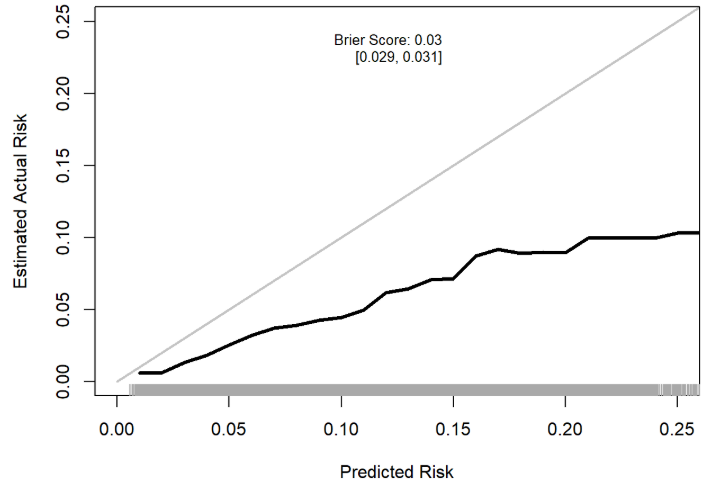


Calibration curve by age: In both groups, risks are somewhat overestimated for higher ages

Female Male


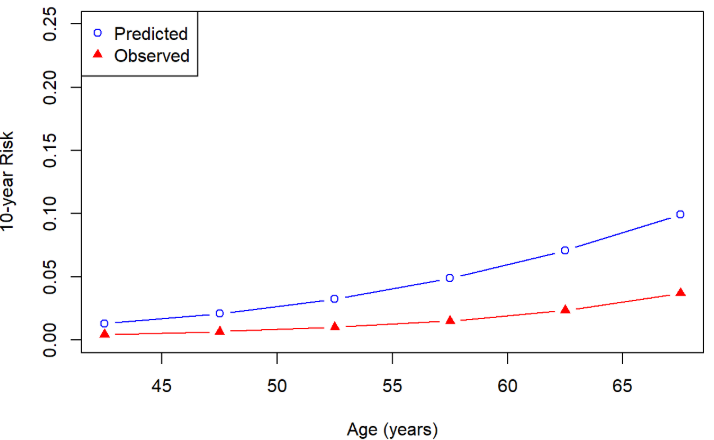

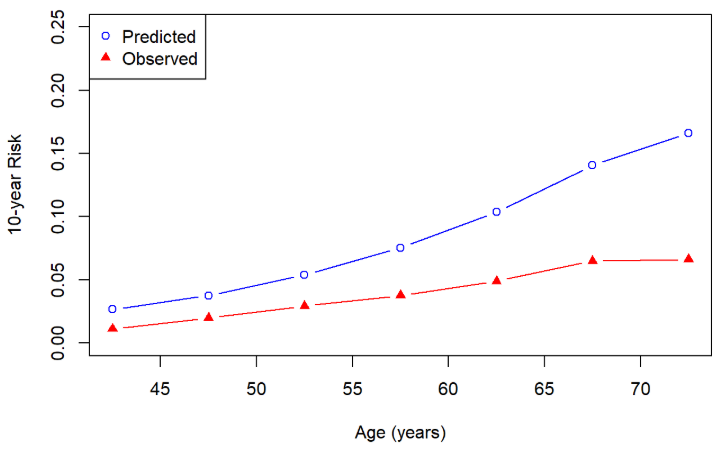


Calibration curve by SBP: In both groups, overestimated risks especially for higher SBP values

Female Male


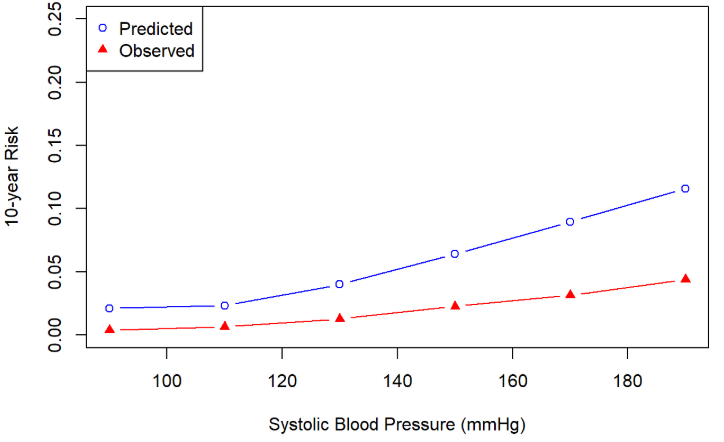

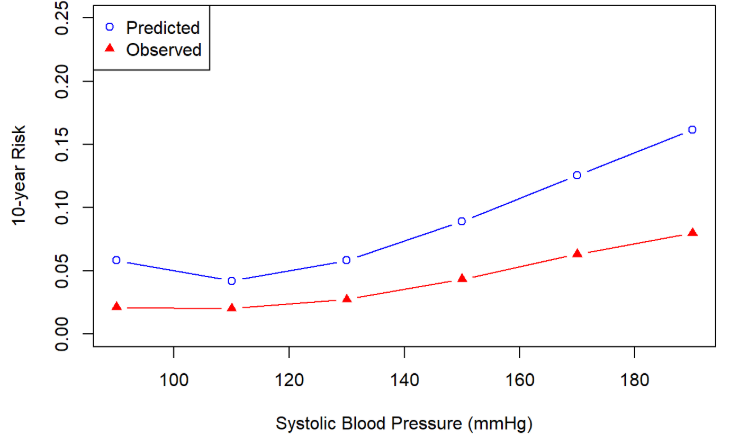


Decile-based calibration plot: Poor calibration. Risk is consistently overestimated, especially at higher probabilities.

Female Male


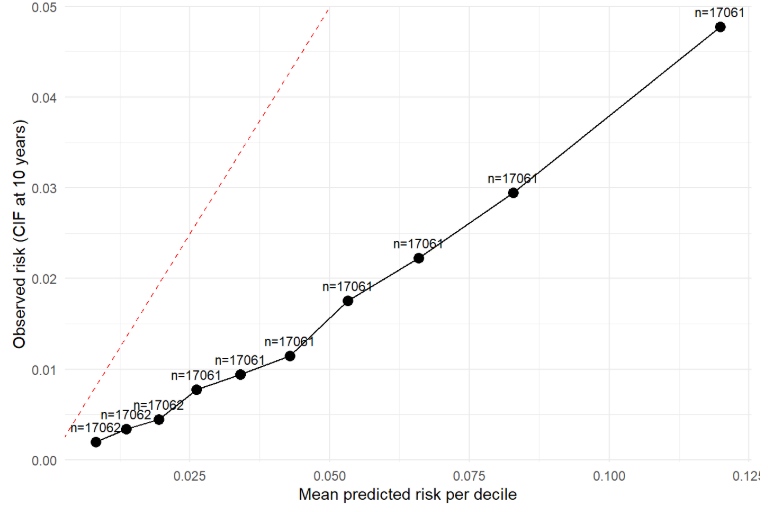

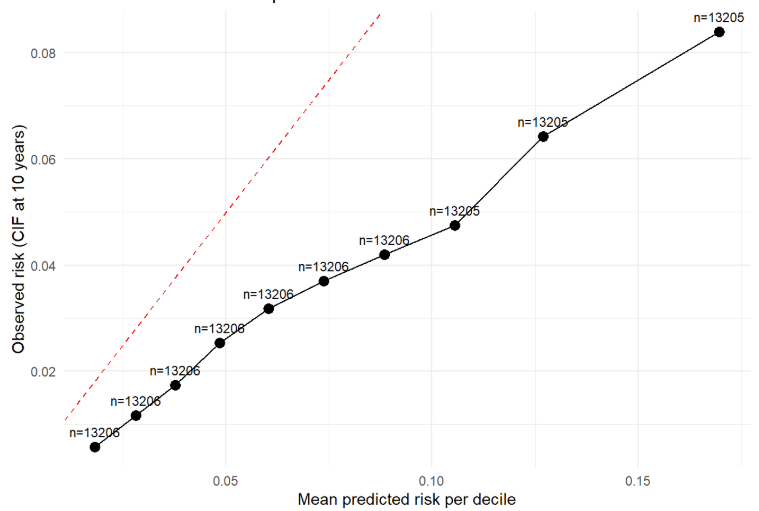


The PREVENT model has limited usefulness using the SCORE2 outcome definition for the UKBB data.

QRISK3 Outcome definition: acceptable accuracy with rather good overall calibration.

Female Male


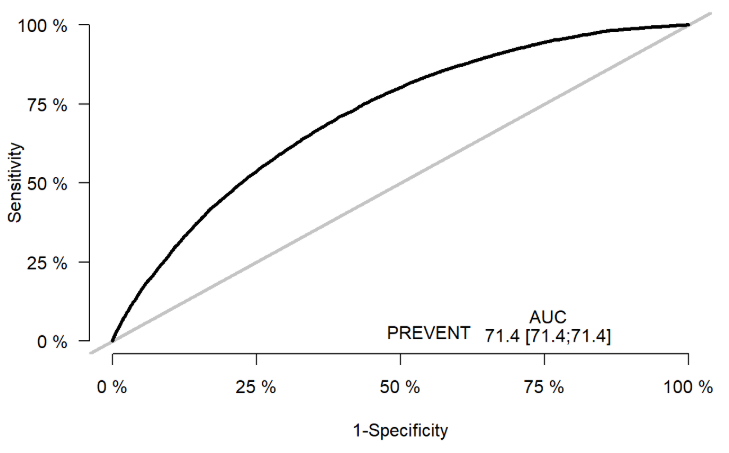

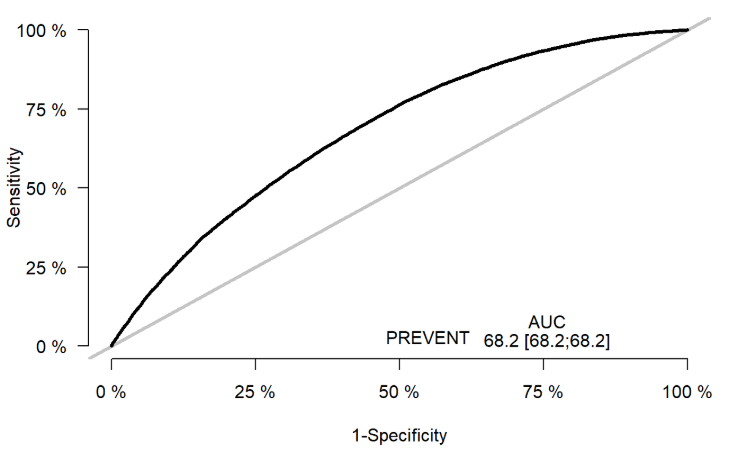


Female Male


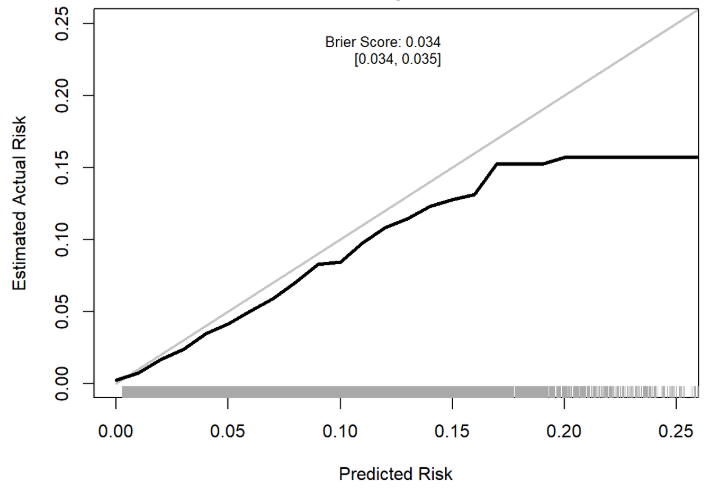

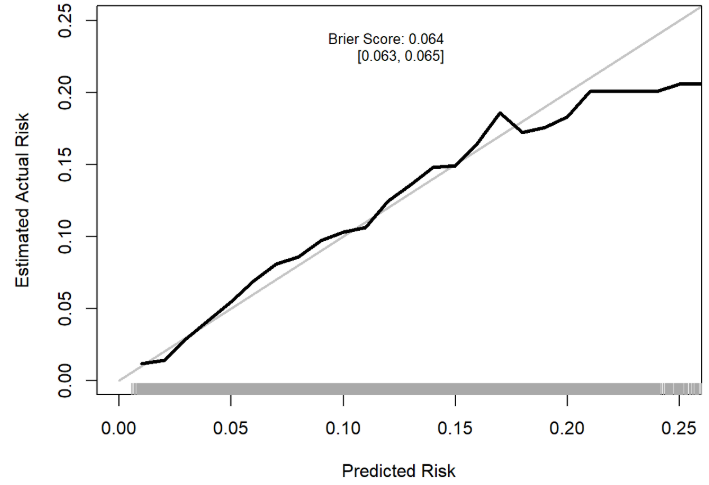


Calibration curve by age: good calibration for younger ages

Female Male

**
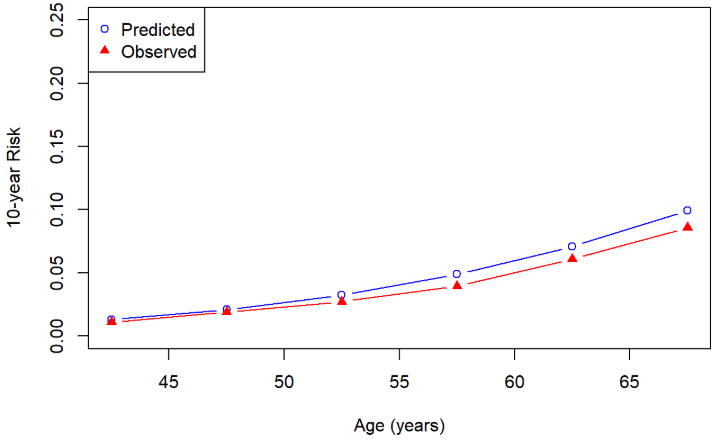
**  **
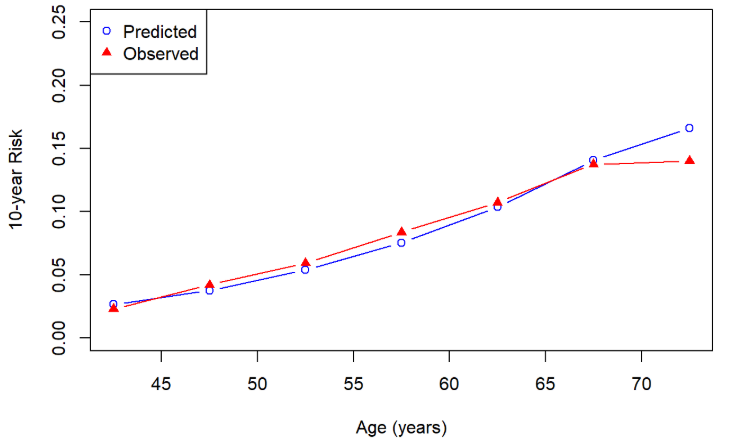
**

Calibration curve by SBP: good calibration for SBP (except >170mmHg for females)

Female Male


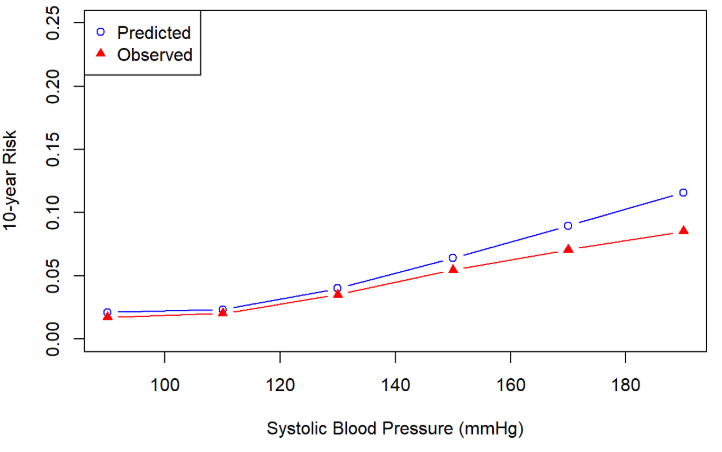

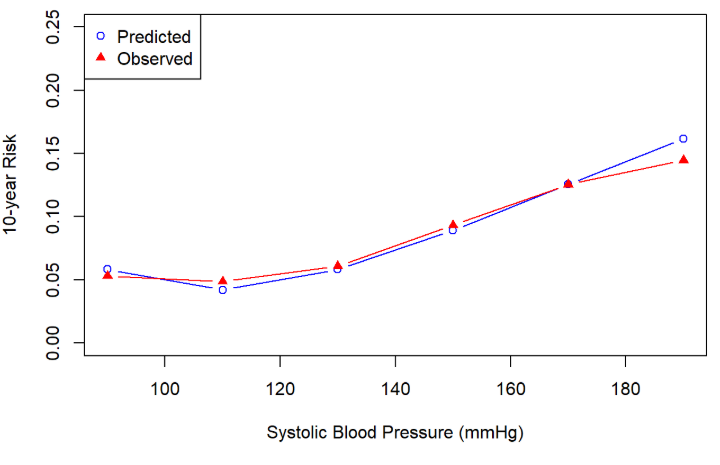


Decile-based calibration plot: Good calibration. Observed and predicted risks closely aligned across all deciles.

Female Male

**
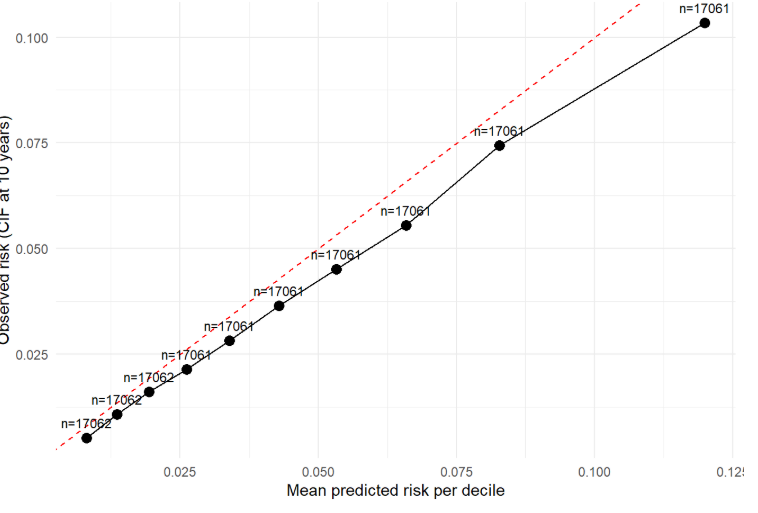

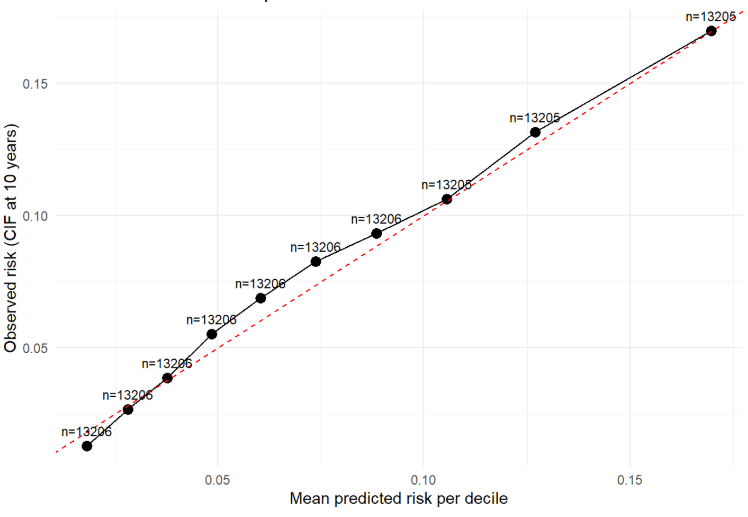
**

The PREVENT model is accurate and rather well-calibrated for the QRISK3 outcome definition

# PREVENT OUTCOME definition: better accuracy for females with good calibration overall for risks < 10%

Female Male


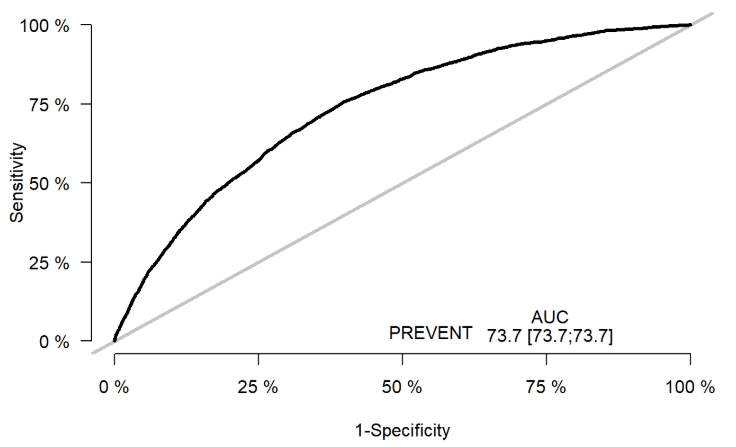

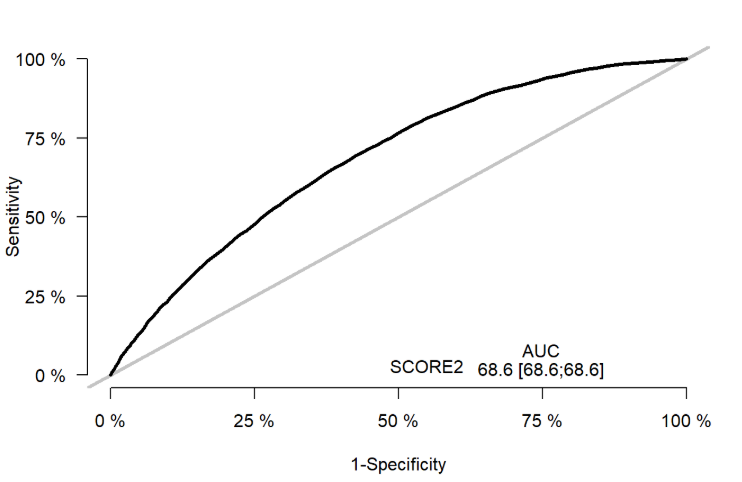


Female Male


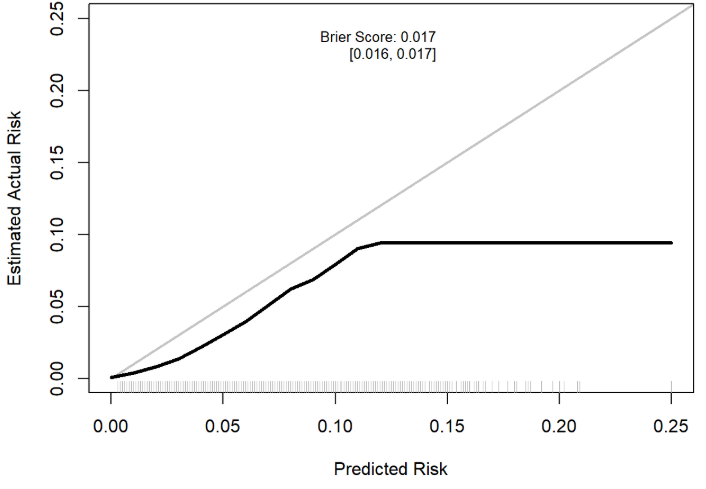

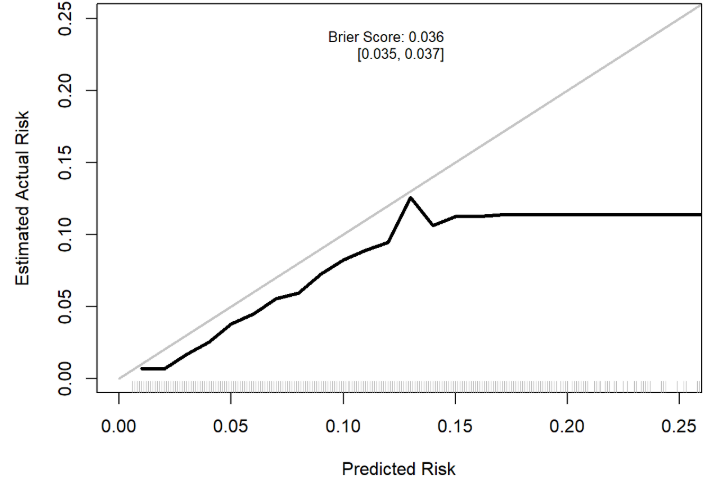


Calibration curve by age: mediocre calibration

Female Male


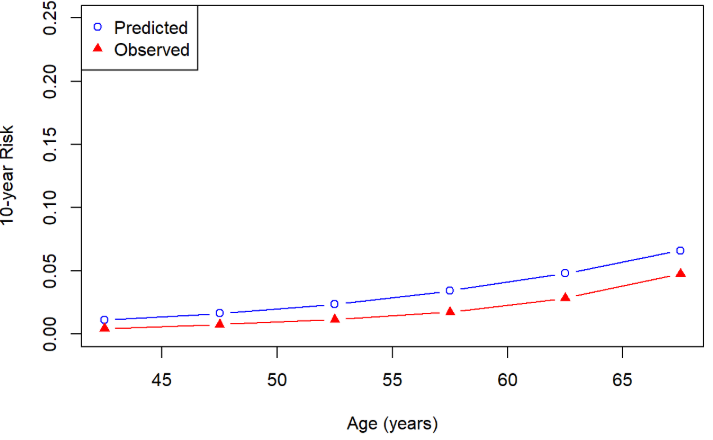

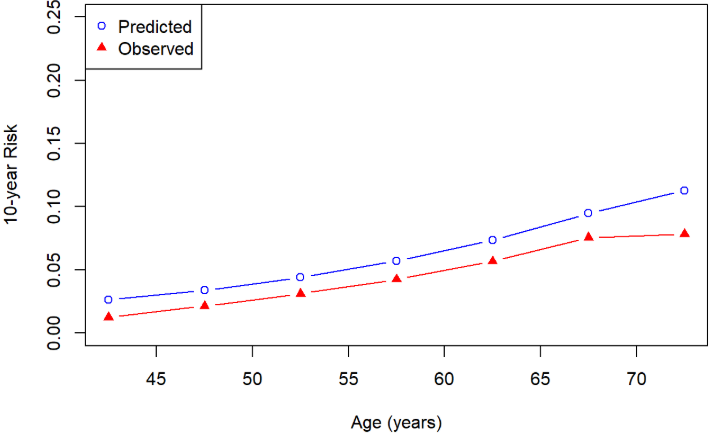


Calibration curve by SBP: mediocre calibration for SBP

Female Male


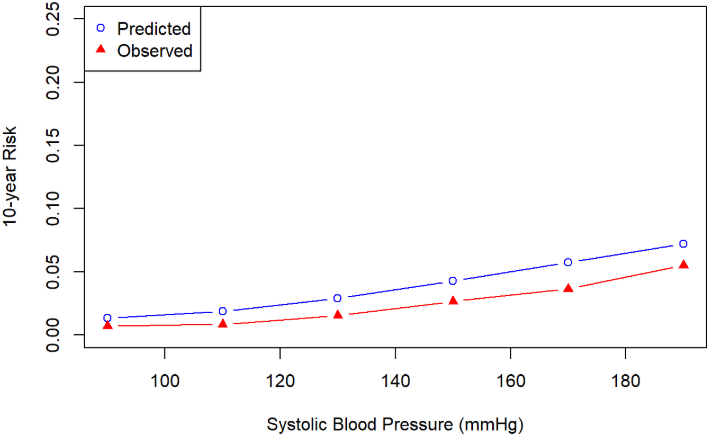

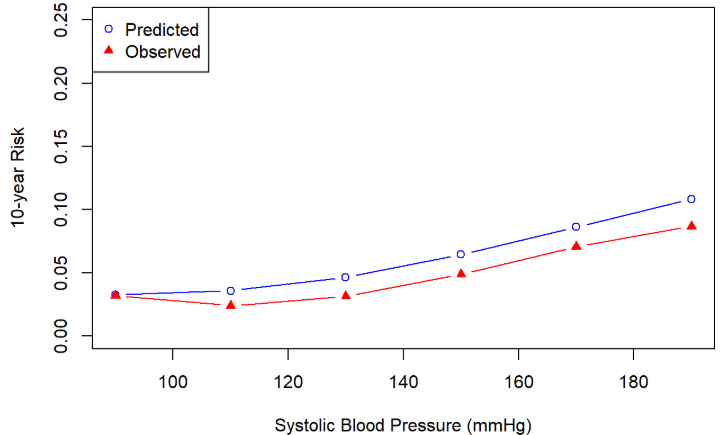


Decile-based calibration plot: Moderate calibration, with observed risks lower than predicted across deciles, indicating systematic overestimation.

Female Male


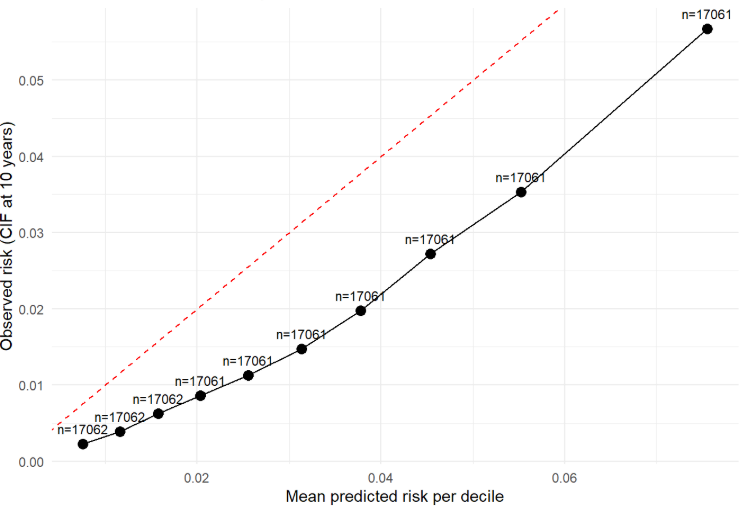

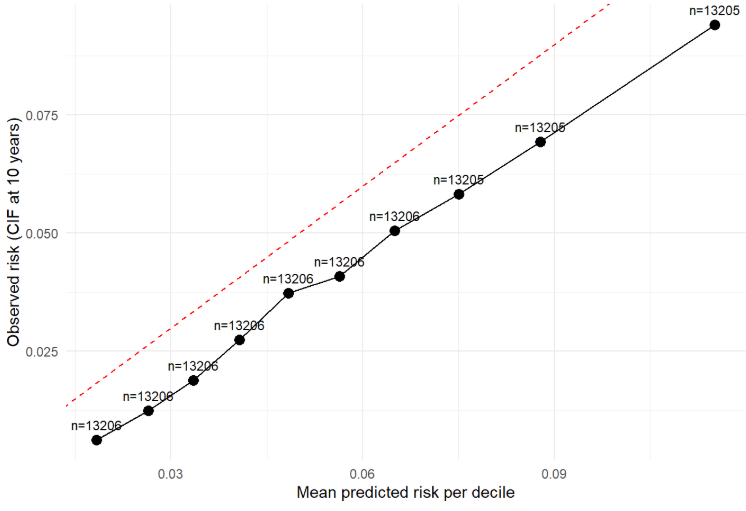


The SCORE2 model shows mediocre calibrations for the PREVENT outcome definition.

SCORE2 OUTCOME definition: better accuracy for females with mediocre visual calibration for both groups, some overestimation of risks exists.

Female Male


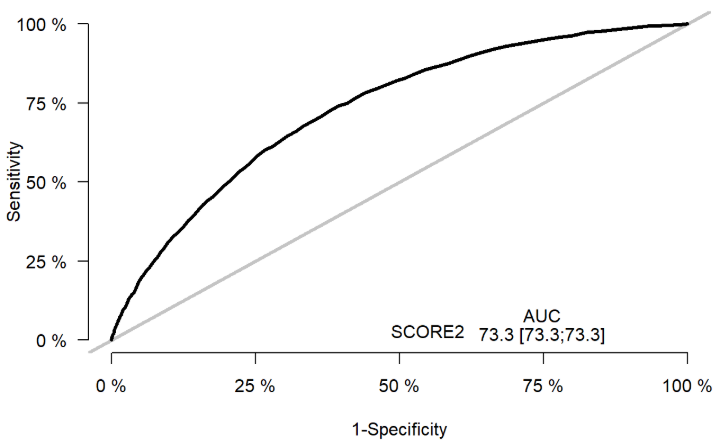

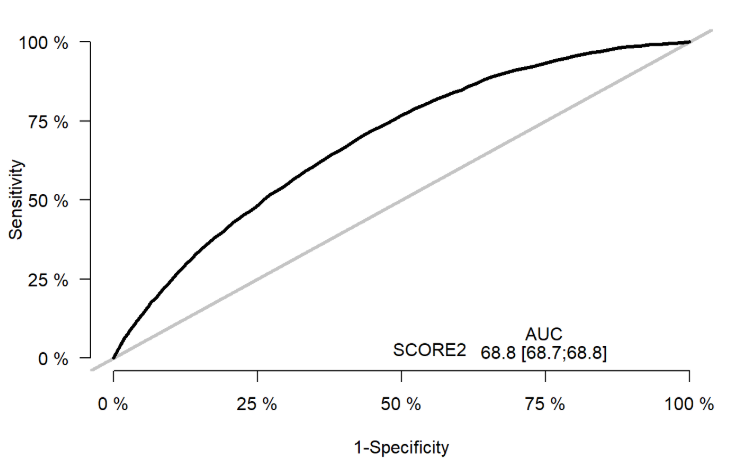


Female Male


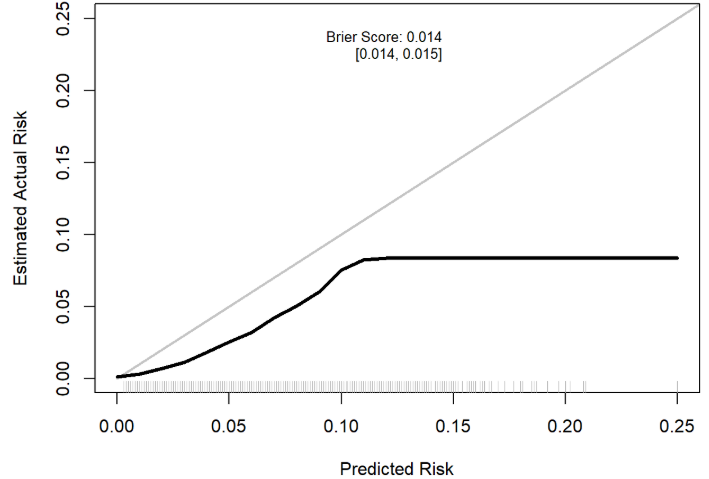

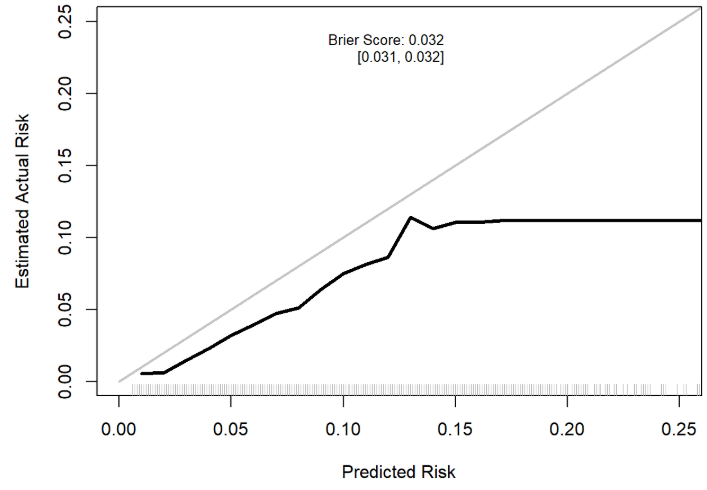


Calibration curve by age: mediocre calibration. The risk is overestimated as age increases

Female Male


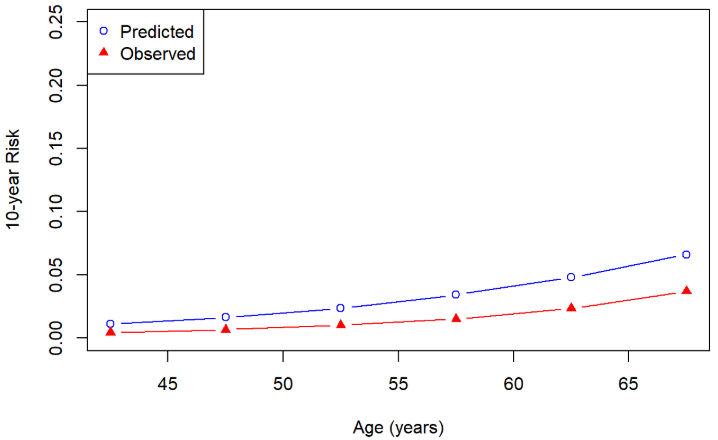

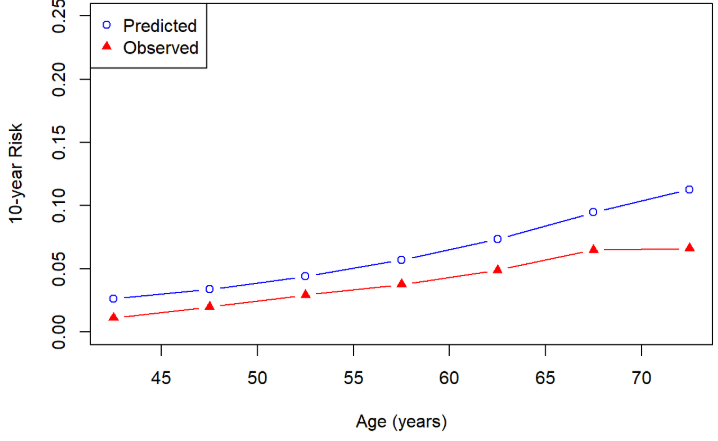


Calibration curve by SBP: mediocre calibration

Female Male


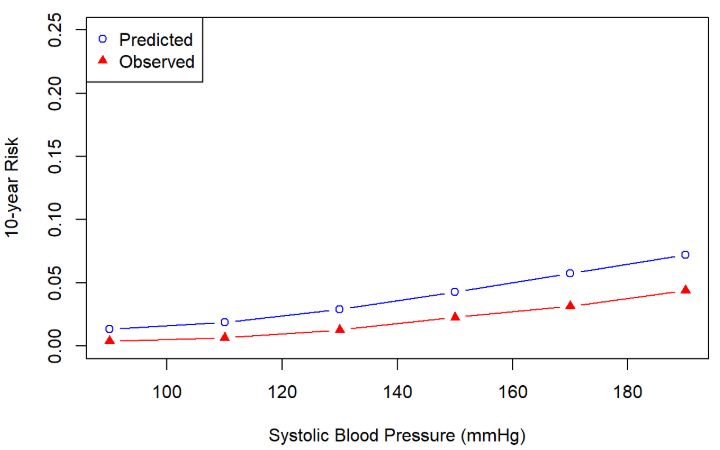

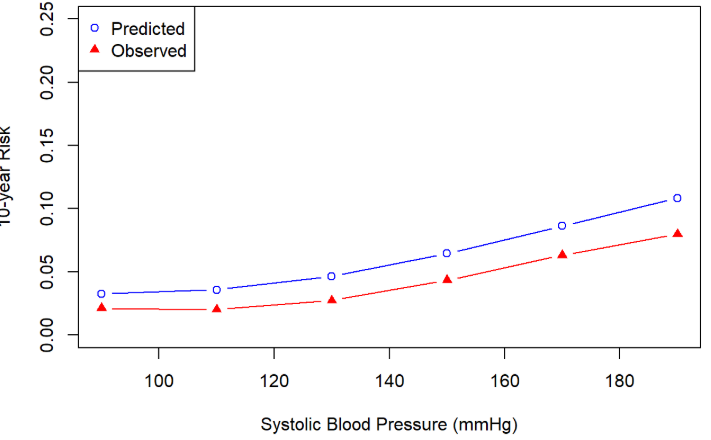


Decile-based calibration plot: Moderate calibration, with observed risks lower than predicted across deciles, indicating systematic overestimation.

.

Female Male


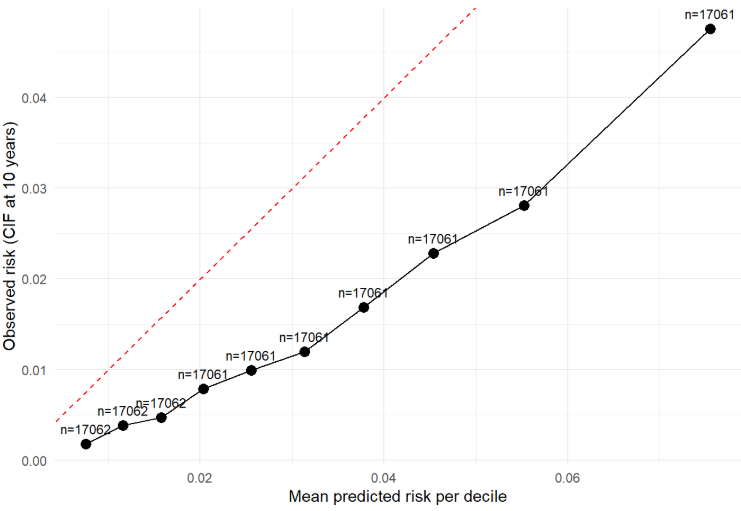

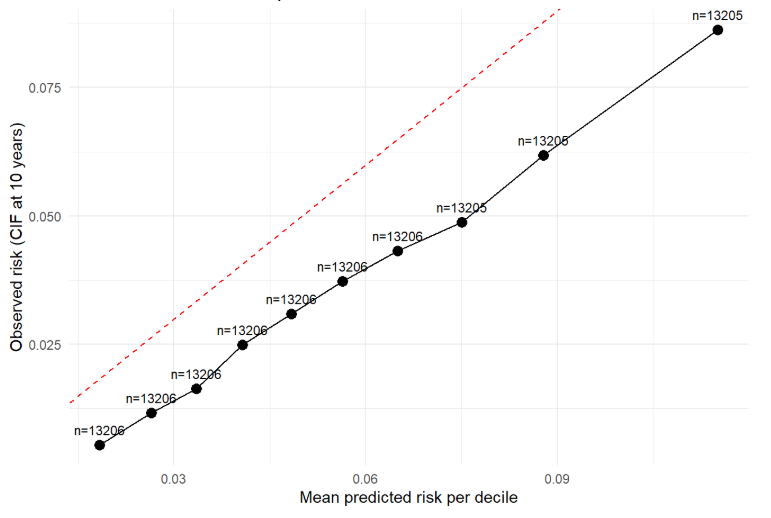


The SCORE2 model shows limited usefulness using the SCORE2 outcome definition for the UKBB data.

QRISK3 Outcome definition: better accuracy for females but with poor calibration mostly underestimating risks for both groups

Female Male


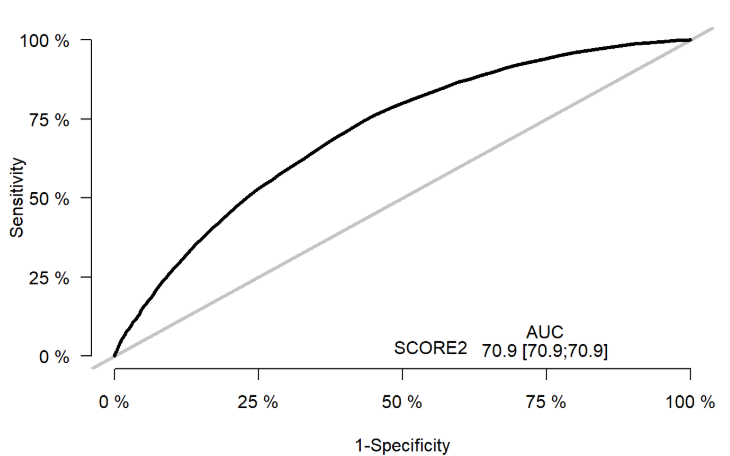

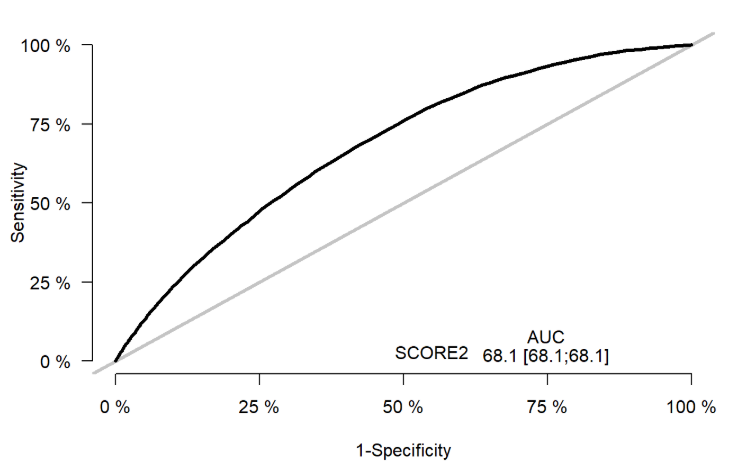


Female Male


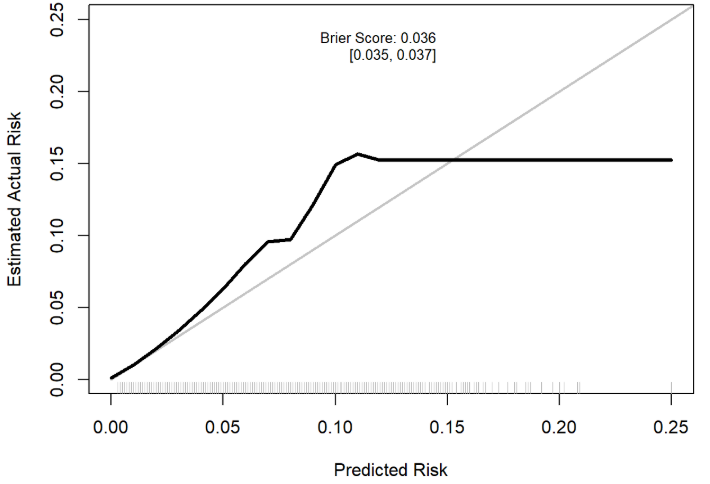

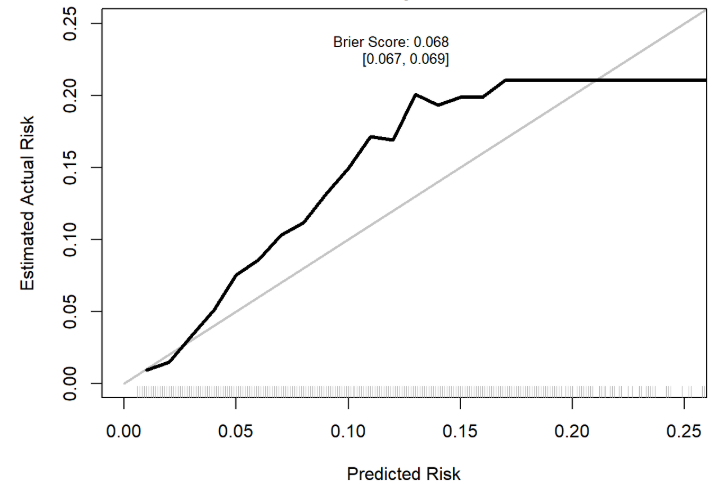


Calibration curve by age: better calibration for females with underestimated risks for higher ages especially in the male population

Female Male


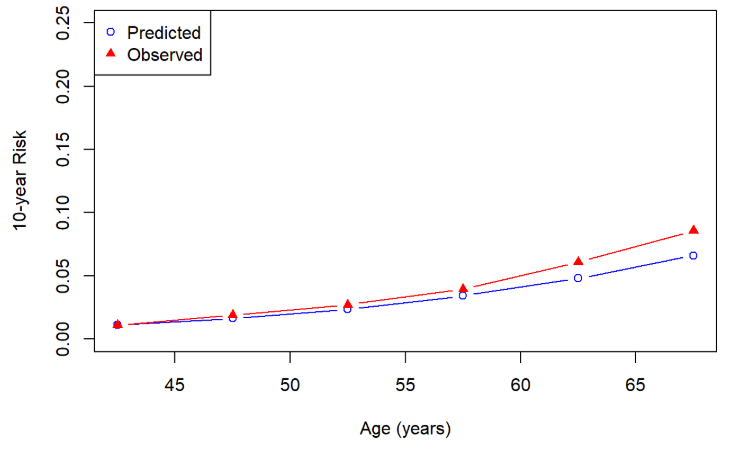

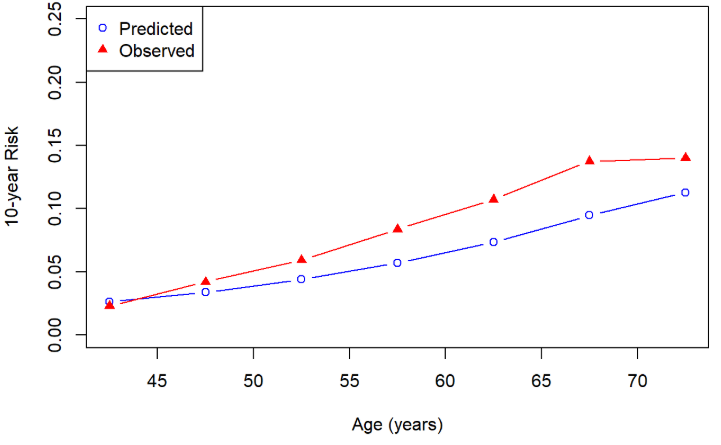


Calibration curve by SBP: good calibration for females for usual ranges & mediocre calibration for males underestimating risks for standard ranges

Female Male


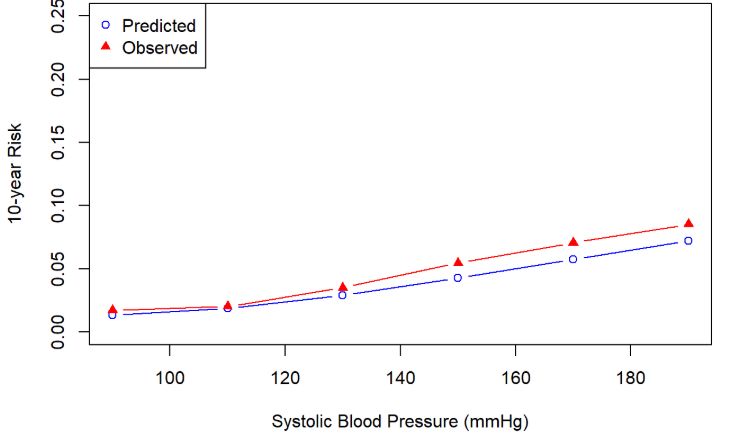

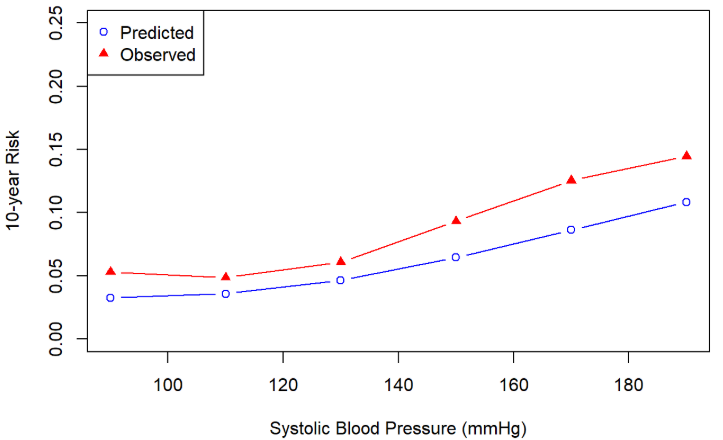


Decile-based calibration plot: Poor calibration, with systematic underestimation of risk across deciles, especially at higher probabilities.

Female Male


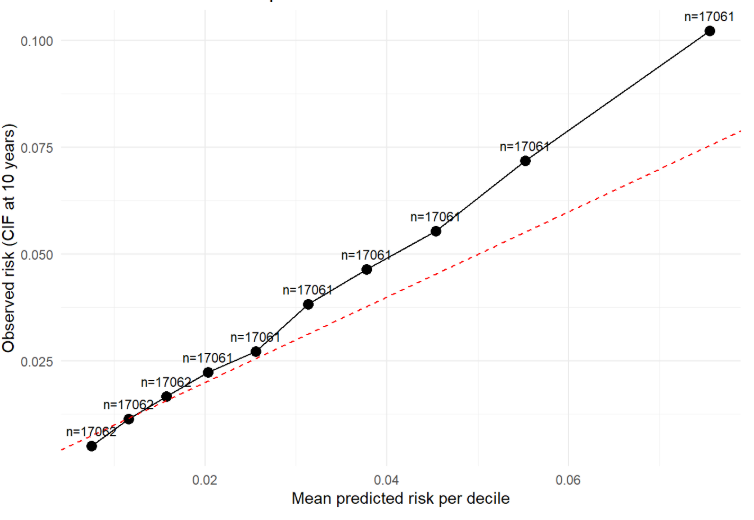

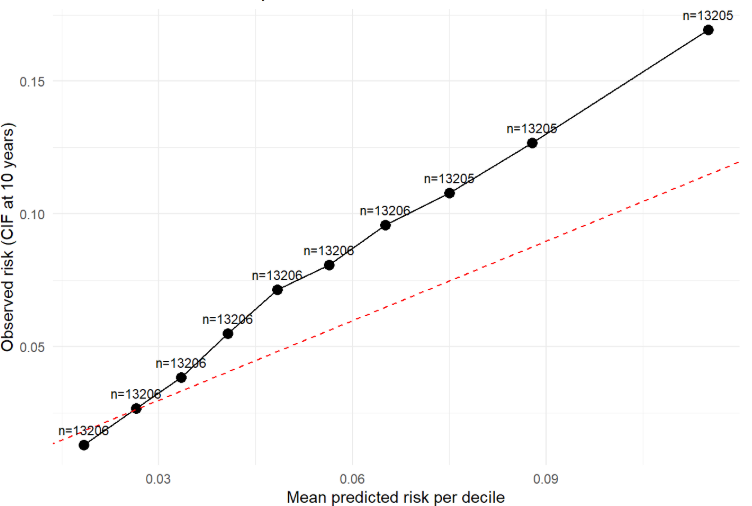


The SCORE2 main/OP model is mediocre for the QR3 outcome definition.
