# Supplementary material for: Independent external validation and head-to-head comparison of guideline-recommended CVD risk prediction models
Source: Am J Prev Cardiol. 2026 Apr 11;28:101625. doi: 10.1016/j.ajpc.2026.101625 (PMC13326136; doi:10.1016/j.ajpc.2026.101625)
Supplement: Supplementary file 4 [file mmc4.docx]

**SUPPLEMENTARY NOTES: Technical details**

**Detailed statistical analysis plan.**

For the implementation of the **QRISK3** algorithm, only individuals who had not taken cholesterol-lowering medication and who had a recorded value for the Townsend Deprivation Index at recruitment were included in the analysis. The variables used in the model included age (years), gender (female/male), Townsend Deprivation Score, body mass index (BMI; kg/m²), total cholesterol to HDL cholesterol ratio, systolic blood pressure (mmHg), standard deviation of systolic blood pressure, ethnicity (categorized as White or not recorded, Indian, Pakistani, Bangladeshi, Other Asian, Black Caribbean, Black African, Chinese, or Other), and smoking status (Non-smoker, Former smoker, Light smoker, Moderate smoker, Heavy smoker). Additional binary variables (coded as yes = 1, no = 0) included: family history of coronary heart disease in a first-degree relative aged <60 years, type 1 diabetes, type 2 diabetes, antihypertensive treatment, rheumatoid arthritis, atrial fibrillation, chronic kidney disease (stage 4 or 5), migraine, corticosteroid use, HIV/AIDS, systemic lupus erythematosus, atypical antipsychotic use, severe mental illness, and erectile dysfunction (men only).

The following variables have been selected for the implementation of the QRISK3 model:

- Age.at.recruitment as age
- has_atrial_fibrillation as atrial_fibrillation
- has_taken_antipsychotics as atypical_antipsy
- has_taken_steroids as regular_steroid_tablets
- has_erectile_dysfunction as erectile_disfunction
- has_migraine as migraine
- has_rheumatoid_arthritis as rheumatoid_arthritis
- has_ckd as chronic_kidney_disease
- has_severe_mental_illness as severe_mental_illness
- has_systemic_lupus_erythematosus as systemic_lupus_erythematosis
- has_taken_blood_pressure_medication as blood_pressure_treatment
- has_diabetes_type_1 as diabetes1
- has_diabetes_type_2 as diabetes2
- weight as weight
- height as height
- parental_history_of_heart_disease as heart_attack_relative
- `Cholesterol...Instance.0` / `HDL.cholesterol...Instance.0` as cholesterol_HDL_ratio
- systolic_blood_pressure as systolic_blood_pressure
- std_systolic_blood_pressure as std_systolic_blood_pressure
- smoking_category as smoke
- Townsend.deprivation.index.at.recruitment as townsend
- Sex as gender
- ethnicity as ethiniciy with the following categorization:

"White","British","Irish", "Any other white background" ~1,

"Indian" ~ 2

"Pakistani" ~ 3

"Bangladeshi" ~ 4

"Any other Asian background"~ 5

"Caribbean" ~ 6

"African" ~ 7

"Chinese" ~ 8

other ~ 9

Subsequently, only individuals with complete data for all the above variables were retained in the dataset used to implement the QRISK3 algorithm. The implementation was conducted using the QRISK3_2017 function from the QRISK3 R package [12]. For the evaluation of the QRISK3 model under each outcome definition, the dataset was further filtered by sex to include only participants without a positive outcome prior to attendance, based on the respective outcome definition and those with a total cholesterol to HDL cholesterol ratio between 1 and 11.

For the implementation of the **PREVENT** model, the variables used in the analysis included total cholesterol (mg/dL), high-density lipoprotein (HDL) cholesterol (mg/dL), age (years), diabetes status (yes = 1, no = 0), smoking status (yes = 1, no = 0), antihypertensive treatment (yes = 1, no = 0), statin treatment (yes = 1, no = 0), systolic blood pressure (mmHg), body mass index (BMI; kg/m²), and estimated glomerular filtration rate (eGFR; mL/min per 1.73 m²).

The PREVENT model can run with missing data for certain variables, such as ACR, which has many missing values. However, it cannot handle missing data in other variables like Total Cholesterol, HDL Cholesterol, Systolic Blood Pressure (SBP), Body Mass Index (BMI) and estimated Glomerular Filtration Rate (eGFR). The variables Total Cholesterol, HDL Cholesterol, Systolic Blood Pressure (SBP), and Body Mass Index (BMI) were filtered to ensure they fall within specific value ranges, resulting in no missing values.

Specifically, Total Cholesterol values were restricted to the range of 130–320 mg/dL, HDL Cholesterol to 20–100 mg/dL, Systolic Blood Pressure to 90–200 mmHg, and BMI to 18.5–40 kg/m². No filtering was applied to the estimated Glomerular Filtration Rate (eGFR), resulting in missing cardiovascular disease (CVD) risk estimates for individuals lacking eGFR values. Consequently, such observations were excluded from the evaluation of the PREVENT model across all outcome definitions. All participants in the UK Biobank (UKBB) dataset were aged between 37 and 73 years. Therefore, no additional age-based filtering was necessary to meet the PREVENT model's requirement for individuals aged 30 to 79. To apply the PREVENT algorithm, the sex-specific base equations of the PREVENT 10-year risk estimation model, developed for composite cardiovascular disease (CVD) outcomes, as described in [13], were used.

The following variables from the dataset were considered:

- "Age.at.recruitment"
- "has_diabetes"
- "is_smoking"
- "has_taken_cholesterol_lowering_medication"
- "has_taken_blood_pressure_medication"
- "systolic_blood_pressure"
- "Cholesterol...Instance.0"
- "HDL.cholesterol...Instance.0"
- "egfr"

The implementation was carried out using the outlined model equation for females:


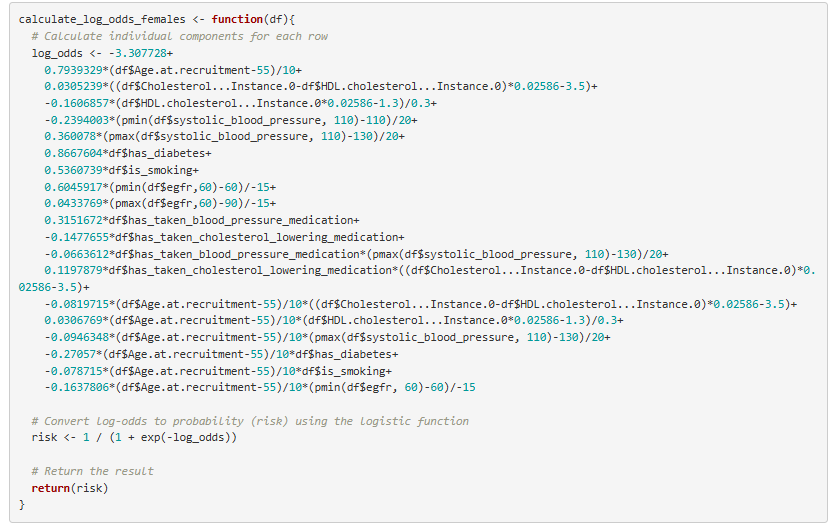


and this one for males:


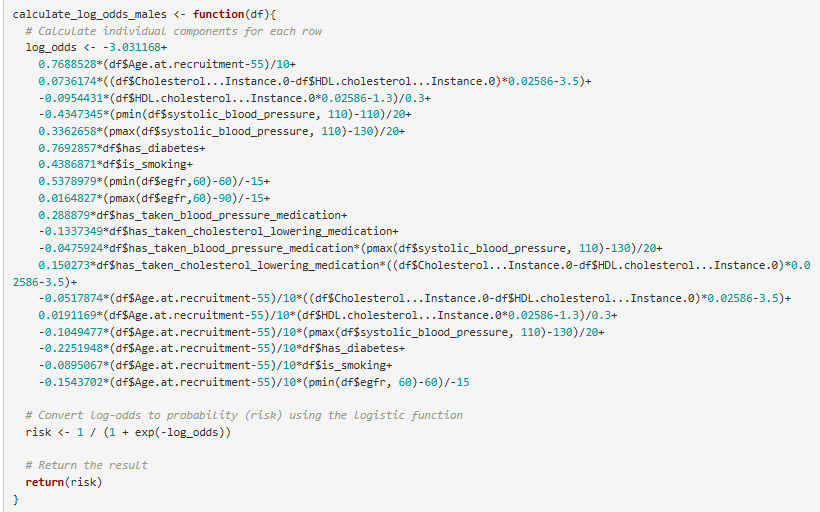


The evaluation of the PREVENT algorithm on each outcome was carried out separately by gender, with the data filtered to retain only subjects without a positive outcome prior to attendance, based on the respective outcome definition.

For the implementation of the **SCORE2** model, the RiskScorescvd R package [14] was used. If a participant had type II diabetes and was between 40 and 69 years of age, their 10-year cardiovascular disease (CVD) risk was estimated using the SCORE2_Diabetes function. For those aged 40 to 69 without diabetes at baseline as well as for participants aged 70 years and over (older population, OP), the SCORE2 function was applied. This function internally incorporates the appropriate age and sex-specific risk equations, including those corresponding to individuals aged 70 and above. Both functions were parameterized for the low-risk region, as the analysis was conducted using data from individuals living in the UK.

Risk estimation using the main SCORE2 and SCORE2-OP models was based on the following input variables: gender (male/female), age (years), diabetes status (yes = 1, no = 0), smoking status (yes = 1, no = 0), systolic blood pressure (mmHg), total cholesterol (mmol/L), and HDL cholesterol (mmol/L). For the SCORE2-Diabetes model, diabetes status was replaced with type II diabetes status (yes = 1, no = 0), and the following additional variables were included: age at diabetes diagnosis, estimated glomerular filtration rate (eGFR; mL/min per 1.73 m²), and glycated haemoglobin (HbA1c).

In the dataset, the following variable names were used for the implementation of the main SCORE2 and SCORE2-OP models:

- Age.at.recruitment as Age
- has_diabetes as diabetes
- is_smoking as smoker
- systolic_blood_pressure as systolic.bp
- Cholesterol...Instance.0 as total.chol
- HDL.cholesterol...Instance.0 as total.hdl
- Sex as Gender

For the SCORE2-Diabetes model, the same variables were used with the following modifications and additions:

- Age.diabetes.diagnosed...Instance.0 as diabetes.age
- egfr as eGFR
- Glycated.haemoglobin..HbA1c....Instance.0 as HbA1c
- has_diabetes_type_2 instead of has_diabetes as diabetes

The CVD risk result was set to NA for subjects with missing values in at least one of the variables: systolic.bp, total.chol, total.hdl and/or HbA1c, eGFR, diabetes.age. Additionally, individuals aged 40–69 years with diabetes other than type II were assigned an NA result. A total of 84,695 subjects had an NA result and were excluded from the evaluation of the SCORE2 main/OP/Diabetes algorithm across all three outcome definitions. Analyses were conducted by sex, retaining only subjects without a positive outcome prior to attendance, based on the respective outcome definition, as described in the previous models’ implementations.
