# Supplementary material for: Independent external validation and head-to-head comparison of guideline-recommended CVD risk prediction models
Source: Am J Prev Cardiol. 2026 Apr 11;28:101625. doi: 10.1016/j.ajpc.2026.101625 (PMC13326136; doi:10.1016/j.ajpc.2026.101625)
Supplement: Supplementary file 5 [file mmc5.docx]

**Table SD1.** Summary of the event rates and performance metrics on for the implementation of the models across the three label definitions, separately for female and male population in the UKBB

| **Algorithm** | **Outcome Definition** | **Females** | | | | **Males** | | | |
| --- | --- | --- | --- | --- | --- | --- | --- | --- | --- |
|  |  | **AUC**  **(95% CI)** | **Brier score** | **N** | **Events%** | **AUC**  **(95% CI)** | **Brier score** | **N** | **Events %** |
|  |  |  |  |  |  |  |  |  |  |
| QRISK3 | PREVENT | 0.7438  (0.7436-0.7440) | 0.0160 | 198,846 | 2.00 | 0.6998  (0.6996-0.6999) | 0.0314 | 148,772 | 4.37 |
|  | SCORE2 | 0.7377  (0.7375-0.7378) | 0.0132 | 198,370 | 1.64 | 0.6958  (0.6956-0.6959) | 0.0276 | 148,533 | 3.82 |
|  | QRISK3 | 0.7228  (0.7228-0.7228) | 0.0331 | 196,378 | 4.44 | 0.6958  (0.6958-0.6958) | 0.0565 | 146,889 | 8.70 |
| SCORE2  MAIN/OP/  DIABETES | PREVENT | 0.7274  (0.7272-0.7276) | 0.0212 | 222,978 | 2.35 | 0.6813  (0.6811-0.6815) | 0.0430 | 182,571 | 5.02 |
|  | SCORE2 | 0.7259  (0.7256-0.7261) | 0.0170 | 221,579 | 1.89 | 0.6836  (0.6833-0.6839) | 0.0366 | 181,187 | 4.26 |
|  | QRISK3 | 0.7048  (0.7048-0.7049) | 0.0424 | 216,188 | 5.06 | 0.6750  (0.6750-0.6750) | 0.0774 | 173,358 | 9.85 |
| PREVENT | PREVENT | 0.7382  (0.7380-0.7384) | 0.0193 | 210,155 | 2.28 | 0.6864  (0.6862-0.6867) | 0.0400 | 177,939 | 4.97 |
|  | SCORE2 | 0.7300  (0.7300-0.7302) | 0.0157 | 208,847 | 1.85 | 0.6819  (0.6817-0.6821) | 0.0340 | 176,604 | 4.23 |
|  | QRISK3 | 0.7132  (0.7130-0.7134) | 0.0393 | 203,748 | 4.96 | 0.6793  (0.6791-0.6795) | 0.0721 | 169,368 | 9.80 |

**Table SD2**. Overall Calibration Slope and Intercept (with 95% Confidence Intervals) for Each Combination of Algorithm and Outcome Definition.

| **Mean (95% CI)** | | | | | | | |
| --- | --- | --- | --- | --- | --- | --- | --- |
|  | **Outcome definition** | **PREVENT** | | **SCORE2** | | **QRISK3** | |
| **Algorithm** | | **Women** | **Men** | **Women** | **Men** | **Women** | **Men** |
| **QRISK3** | | | | | | | |
| *Slope* | | 0.139  (0.118, 0.159) | 0.152  (0.134, 0.171) | 0.099  (0.085, 0.113) | 0.132  (0.116, 0.149) | 0.192  (0.161, 0.222) | 0.235  (0.204, 0.266) |
| *Intercept* | | 0.041  (0.031, 0.051) | 0.041  (0.032, 0.050) | 0.032  (0.025, 0.039) | 0.037  (0.028, 0.045) | 0.075  (0.060, 0.089) | 0.080  (0.064, 0.095) |
| **PREVENT** | | | | | | | |
| *Slope* | | 0.212  (0.175, 0.248) | 0.305  (0.258, 0.351) | 0.183  (0.152, 0.213) | 0.238  (0.201, 0.276) | 0.263  (0.207, 0.319) | 0.391  (0.322, 0.460) |
| *Intercept* | | 0.034  (0.023, 0.046) | 0.045  (0.030, 0.060) | 0.026  (0.017, 0.035) | 0.039  (0.027, 0.052) | 0.068  (0.051, 0.085) | 0.085  (0.064, 0.107) |
| **SCORE2** | | | | | | | |
| *Slope* | | 0.224  (0.153, 0.296) | 0.202  (0.140, 0.264) | 0.183  (0.129, 0.238) | 0.191  (0.137, 0.244) | 0.213  (0.129, 0.298) | 0.276  (0.181, 0.371) |
| *Intercept* | | 0.071  (0.050, 0.091) | 0.088  (0.067, 0.108) | 0.058  (0.041, 0.074) | 0.076  (0.058, 0.094) | 0.109  (0.085, 0.134) | 0.142  (0.112, 0.172) |

**Table SD3a**. Discrimination performance of SCORE2 models using coefficients derived by excluding the UKBB from the developing cohorts.

| ***Outcome definition*** | ***N*** | | **SCORE2 Equations** | | | |
| --- | --- | --- | --- | --- | --- | --- |
|  |  |  | ***AUC***  ***(95% CI)*** | | ***Brier Score*** | |
|  |  |  |  |  | ***Overall*** | |
|  | *Females* | *Males* | *Females* | *Males* | *Females* | *Males* |
| *PREVENT* | 222,978 | 182,571 | 0.7258 (0.7255, 0.7260) | 0.6806 (0.6804, 0.6807) | 0.0212 (0.0207, 0.0218) | 0.0432 (0.0423, 0.0440) |
| *SCORE2* | 221,579 | 181,187 | 0.7240 (0.7238, 0.7243) | 0.6811 (0.6809, 0.6814) | 0.0170 (0.0165, 0.0176) | 0.0367 (0.0359, 0.0375) |
| *QRISK3* | 216,188 | 173,358 | 0.7030 (0.7029, 0.7030) | 0.6742 (0.6742, 0.6742) | 0.0425 (0.0417, 0.0433) | 0.0777 (0.0766, 0.0789) |

**Table SD3b**. Calibration slope and intercept of SCORE2 models using coefficients derived by excluding the UKBB from the developing cohorts.

| **Mean (95% CI)** | | | | | | | |
| --- | --- | --- | --- | --- | --- | --- | --- |
| **Algorithm** | **Outcome definition** | **PREVENT** | | **SCORE2** | | **QRISK3** | |
|  |  | **Women** | **Men** | **Women** | **Men** | **Women** | **Men** |
| **SCORE2 Equations** | | | | | | | |
| *Calibration Slope* | | 0.128  (0.069, 0.188) | 0.136  (0.088, 0.183) | 0.100  (0.054, 0.147) | 0.119  (0.079, 0.160) | 0.151  (0.079, 0.223) | 0.187  (0.114, 0.259) |
| *Calibration Intercept* | | 0.084  (0.064, 0.105) | 0.104  (0.085, 0.124) | 0.070  (0.054, 0.087) | 0.090  (0.073, 0.108) | 0.121  (0.096, 0.145) | 0.162  (0.134, 0.190) |

QRISK3 MODEL

| Characteristics | Derivation cohort | | Validation cohort | | UKBB | | | | | |
| --- | --- | --- | --- | --- | --- | --- | --- | --- | --- | --- |
|  |  |  |  |  | Prevent label | | Score2 label | | Qrisk3 label | |
|  | Women n=4,019,956 | Men n=3,869,847 | Women=  1,360,457 | Men=  1,310,841 | Women=  198,846 | Men=  148,772 | Women=  198,370 | Men=  148,533 | Women=  196,378 | Men=  146,889 |
| Mean (SD) age (years) | 43.3 (15.3) | 42.6 (14.0) | 43.3 (15.3) | 42.6 (13.8) | 55.61 (7.98) | 55.33 (8.21) | 55.61 (7.98) | 55.32 (8.21) | 55.56 (7.98) | 55.27 (8.21) |
| Mean (SD) Townsend score | 0.4 (3.2) | 0.5 (3.3) | 0.4 (3.3) | 0.5 (3.3) | -1.437 (2.98) | -1.356 (3.09) | -1.439 (2.98) | -1.358 (3.09) | -1.446 (2.98) | -1.366 (3.09) |
| Mean (SD) body mass index kg/m2 | 25.4 (5.1) | 25.9 (4.2) | 25.4 (5.1) | 25.9 (4.2) | 26.69 (4.96) | 27.38 (4.02) | 26.69 (4.96) | 27.38 (4.02) | 26.67 (4.95) | 27.37 (4.01) |
| Mean (SD) total cholesterol: HDL cholesterol ratio | 3.7 (1.2) | 4.4 (1.4) | 3.6 (1.2) | 4.4 (1.3) | 3.88 (1.01) | 4.62 (1.15) | 3.88 (1.01) | 4.62 (1.15) | 3.88 (1.01) | 4.62 (1.15) |
| Mean (SD) systolic blood pressure (mm Hg) | 123.2 (18.2) | 129.2 (16.3) | 123.1(18.1) | 128.8 (16.2) | 134.5 (19.13) | 140.4 (17.33) | 134.5 (19.13) | 140.4 (17.34) | 134.4 (19.13) | 140.4 (17.33) |
| Ethnic origin: n (%) |  |  |  |  |  |  |  |  |  |  |
| White or not recorded | 3,564,651 (88.7) | 3,435,408 (88.8) | 1,218,391 (89.6) | 1,171,281 (89.4) | 188,143  (94.62) | 140,442  (94.39) | 187,698  (94.62) | 140,187  (94.38) | 185,862  (94.65) | 138,673  (94.41) |
| Indian | 77,683  (1.9) | 81,805  (2.1) | 23,146  (1.7) | 26,479  (2.0) | 1,879  (0.94) | 1,625  (1.09) | 1874  (0.94) | 1,620  (1.09) | 1,840  (0.94) | 1,593  (1.08) |
| Pakistani | 39,541  (1.0) | 46,948  (1.2) | 10,919  (0.8) | 14,787  (1.1) | 438  (0.22) | 606  (0.41) | 437  (0.22) | 605  (0.41) | 428  (0.22) | 586  (0.40) |
| Bangladeshi | 31,930  (0.8) | 42,111  (1.1) | 8738  (0.6) | 11,914  (0.9) | 36  (0.02) | 77  (0.05) | 36  (0.02) | 77  (0.05) | 35  (0.02) | 77  (0.05) |
| Other Asian | 53,559 (1.3) | 45,753 (1.2) | 17,078 (1.3) | 15,966 (1.2) | 564  (0.28) | 530  (0.36) | 562  (0.28) | 532  (0.36) | 552  (0.28) | 527  (0.36) |
| Black Caribbean | 37,781  (0.9) | 30,610  (0.8) | 13,142  (1.0) | 10,642  (0.8) | 1,963  (0.99) | 1,105  (0.74) | 1,958  (0.99) | 1,103  (0.74) | 1,928  (0.98) | 1,094  (0.74) |
| Black African | 77,813  (1.9) | 71,245  (1.8) | 27,678  (2.0) | 25,251  (1.9) | 1,116  (0.56) | 1,152  (0.77) | 1,114  (0.56) | 1,154  (0.78) | 1,091  (0.56) | 1,133  (0.77) |
| Chinese | 33,767  (0.8) | 23,730  (0.6) | 8992  (0.7) | 6098  (0.5) | 725  (0.36) | 414  (0.28) | 724  (0.36) | 413  (0.28) | 720  (0.37) | 407  (0.28) |
| Other | 103,231  (2.6) | 92,237  (2.4) | 32,373  (2.4) | 28,423  (2.2) | 3,982  (2.00) | 2,841  (1.91) | 3,967  (2.00) | 2,842  (1.91) | 3,922  (2.00) | 2,799  (1.91) |
| Smoking status: n (%) |  |  |  |  |  |  |  |  |  |  |
| Non-smoker | 2,051,803 (51.0) | 1,463,941 (37.8) | 706,671 (51.9) | 512,252 (39.1) | 121,197  (60.95) | 78,414  (52.71) | 120,932  (60.96) | 78,338  (52.74) | 119,810  (61.01) | 77,609  (52.84) |
| Former smoker | 589,521 (14.7) | 594,265 (15.4) | 194,545 (14.3) | 196,459 (15.0) | 61,009  (30.68) | 52,537  (35.31) | 60,878  (30.69) | 52,445  (35.31) | 60,203  (30.66) | 51,776  (35.25) |
| Light smoker | 434,954 (10.8) | 507,523 (13.1) | 154,565 (11.4) | 177,693 (13.6) | 5,771  (2.90) | 6,704  (4.51) | 5,756  (2.90) | 6,690  (4.50) | 5,689  (2.90) | 6,611  (4.50) |
| Moderate smoker | 226,128  (5.6) | 251,170  (6.5) | 74,933  (5.5) | 84,914  (6.5) | 3,489  (1.75) | 2,881  (1.94) | 3,469  (1.75) | 2,882  (1.94) | 3,441  (1.75) | 2,864  (1.95) |
| Heavy smoker | 115,890  (2.9) | 188,857  (4.9) | 38,218  (2.8) | 64,107  (4.9) | 7,380  (3.71) | 8,236  (5.54) | 7,335  (3.70) | 8,178  (5.51) | 7,235  (3.68) | 8,029  (5.47) |
| Medical characteristics: n (%) |  |  |  |  |  |  |  |  |  |  |
| Family history of coronary heart disease in first degree relative <60 years n (%) | 481,628 (12.0) | 357,987 (  9.3) | 164,023 (12.1) | 123,039 (9.4) | 79,994  (40.23) | 50,466  (33.92) | 79,821  (40.24) | 50,391  (33.93) | 78,758  (40.11) | 49,680  (33.82) |
| Type 1 diabetes n (%) | 10,060  (0.3) | 11,617  (0.3) | 3351  (0.2) | 3932  (0.3) | 204  (0.10) | 188  (0.13) | 201  (0.10) | 184  (0.12) | 190  (0.10) | 173  (0.12) |
| Type 2 diabetes n (%) | 48,022  (1.2) | 58,395  (1.5) | 15,872  (1.2) | 19,318  (1.5) | 2,224  (1.12) | 2,932  (1.97) | 2,219  (1.12) | 2,929  (1.97) | 2,116  (1.08) | 2,826  (1.92) |
| Treated hypertension n (%) | 223,494  (5.6) | 164,255  (4.2) | 77,694  (5.7) | 56,920  (4.3) | 26,233  (13.19) | 19,478  (13.09) | 26,134  (13.17) | 19,477  (13.11) | 25,286  (12.88) | 18,827  (12.82) |
| Rheumatoid arthritis n (%) | 45,700  (1.1) | 20,997  (0.5) | 15,139  (1.1) | 7055  (0.5) | 7,274  (3.66) | 4,179  (2.81) | 7,243  (3.65) | 4,174  (2.81) | 7.096  (3.61) | 4,085  (2.78) |
| Atrial fibrillation n (%) | 15,177  (0.4) | 20,098  (0.5) | 5229  (0.4) | 6874  (0.5) | 1,181  (0.59) | 2,017  (1.36) | 1,227  (0.62) | 2,123  (1.43) | 1,104  (0.56) | 1,956  (1.33) |
| Chronic kidney disease (stage 4 or 5) n (%) | 7518  (0.2) | 6345  (0.2) | 2583  (0.2) | 2165  (0.2) | 2,930  (1.47) | 1,837  (1.24) | 2,930  (1.48) | 1,848  (1.24) | 2,822  (1.44) | 1,773  (1.21) |
| Chronic kidney disease (stage 3, 4, or 5) n (%) | 19,396 (0.5) | 12,254 (0.3) | 6949 (0.5) | 4232 (0.3) |  |  |  |  |  |  |
| Migraine n (%) | 257,825  (6.4) | 103,995  (2.7) | 89,504  (6.6) | 36,141  (2.8) | 27,376  (13.77) | 9,525  (6.40) | 27,253  (13.74) | 9,474  (6.38) | 26,948  (13.72) | 9,333  (6.35) |
| Corticosteroid use n (%) | 96,955  (2.4) | 56,533  (1.5) | 31,775  (2.3) | 18,634  (1.4) | 2,078  (1.05) | 1,416  (0.95) | 2,063  (1.04) | 1.410  (0.95) | 2,005  (1.02) | 1,370  (0.93) |
| HIV/AIDS n (%) | 4332  (0.1) | 7732  (0.2) | 1595  (0.1) | 2945  (0.2) | 53  (0.03) | 238  (0.16) | 53  (0.03) | 235  (0.16) | 53  (0.03) | 232  (0.16) |
| Systemic lupus erythematosus n (%) | 4010  (0.1) | 365  (0.0) | 1349  (0.1) | 134  (0.0) | 448  (0.23) | 49  (0.03) | 437  (0.22) | 48  (0.03) | 427  (0.22) | 48  (0.03) |
| Atypical antipsychotic use n (%) | 19,140  (0.5) | 20,123  (0.5) | 6268  (0.5) | 6597  (0.5) | 745  (0.37) | 502  (0.34) | 740  (0.37) | 502  (0.34) | 730  (0.37) | 486  (0.33) |
| Severe mental illness n (%) | 274,069  (6.8) | 167,115  (4.3) | 94,724  (7.0) | 57,830  (4.4) | 882  (0.44) | 774  (0.52) | 877  (0.44) | 770  (0.52) | 862  (0.44) | 759  (0.52) |
| Erectile dysfunction diagnosis or treatment n (%) | NA | 90,753  (2.3) | NA | 31,136  (2.4) | NA | 823  (0.55) | NA | 820  (0.55) | NA | 800  (0.54) |
| Erectile dysfunction diagnosis | NA | 80,753  (2.1) | NA | 27,727  (2.1) | NA | 377  (0.25) | NA | 374  (0.25) | NA | 369  (0.25) |
| Erectile dysfunction treatment | NA | 28,763  (0.7) | NA | 9877  (0.8) | NA | 495  (0.33) | NA | 494  (0.33) | NA | 479  (0.33) |
| Total CVD events n (%) | 160,549  (4) | 203,016  (5.2) | NA | NA | 9,209  (4.63) | 13,284  (8.93) | 9,152  (4.61) | 13,237  (8.91) | 8717  (4.44) | 12,774  (8.70) |

PREVENT MODEL

| Characteristics | Derivation cohort | | Validation cohort | | UKBiobank | | | | | |
| --- | --- | --- | --- | --- | --- | --- | --- | --- | --- | --- |
|  |  |  |  |  | Prevent label | | Score2 label | | Qrisk3 label | |
|  | Women =  1,839,828 | Men =  1,44,091 | Women=  1,894,882 | Men=  1,435,203 | Women= 210,155 | Men=  177,939 | Women=  208,847 | Men=  176,604 | Women=  203,748 | Men=  169,368 |
| Mean (SD) age (years) | 53±13 | 52±12 | 52±13 | 52±12 | 56±8 | 56±8 | 56±8 | 56±8 | 56±8 | 56±8 |
| Mean (SD) body mass index (kg/m2) | 29±5 | 29±4 | 28±5 | 29±4 | 27 ±4 | 28±4 | 27±4 | 28±4 | 27±4 | 28±4 |
| Mean (SD) total cholesterol (mmol/L) | 5.0±0.8 | 4.9±0.8 | 5.0±0.8 | 4.9±0.8 | 5.8 ±1 | 5.6±1 | 5.8±1 | 5.6±1 | 5.8±1 | 5.6±1 |
| Mean (SD) systolic blood pressure (mm Hg) | 123±16 | 127±15 | 123±16 | 128±15 | 135 ±19 | 141±17 | 135±19 | 141±17 | 147±19 | 141±17 |
| Mean (SD) Non-high-density lipoprotein cholesterol (mmol/L) | 3.4±0.8 | 3.6±0.8 | 3.5±0.8 | 3.6±0.8 | 3.6±0.8 | 3.5±0.8 | 3.6±0.8 | 3.5±0.8 | 3.6±0.8 | 3.6±0.78 |
| Mean (SD ) High-density lipoprotein cholesterol (mmol/L) | 29±5 | 29±4 | 28±5 | 29±4 | 1.6 ±0.3 | 1.3±0.3 | 1.9±0.3 | 1.3±0.3 | 1.6±0.3 | 1.3±0.3 |
| Ethnic origin: % |  |  |  |  |  |  |  |  |  |  |
| White | 78 | 80 | 78 | 80 | 94.44 | 94.27 | 94.44 | 94.26 | 94.46 | 94.25 |
| Black | 10 | 8 | 10 | 8.2 | 1.58 | 1.46 | 1.58 | 1.46 | 1.57 | 1.49 |
| Hispanic | 6 | 5.3 | 4.2 | 3.7 | 0.91 | 0.84 | 0.91 | 0.84 | 0.91 | 0.85 |
| Asian | 2.6 | 2.5 | 2.7 | 2.2 | 2.02 | 2.41 | 2.03 | 2.42 | 2.00 | 2.39 |
| Other or missing | 4.1 | 4.6 | 4.9 | 5.5 | 1.04 | 1.02 | 1.05 | 1.02 | 1.05 | 1.03 |
| Smoking status: n (%) |  |  |  |  |  |  |  |  |  |  |
| Current smoker | 5.8 | 6.2 | 4.7 | 4.9 | 8.7 | 12.2 | 8.7 | 12.2 | 8.7 | 12.2 |
| Never smoker | NA | NA | NA | NA | NA | NA | NA | NA | NA | NA |
| Former smoker | NA | NA | NA | NA | NA | NA | NA | NA | NA | NA |
| Antihypertensive treatment n (%) | 23 | 27 | 24 | 29 | 19 | 23 | 18 | 23 | 17 | 20 |
| Statin treatment n (%) | 14 | 17 | 14 | 17 | 12 | 19 | 12 | 19 | 10 | 16 |
| Mean (SD) Estimated glomerular filtration rate, (mL/min per 1.73 m2) | 91±19 | 91±17 | 91±18 | 91±17 | 96±14 | 95±14 | 96±14 | 95±14 | 96±14 | 95±14 |
| Atherosclerotic cardiovascular disease events n (%) | 31,812 | 34,691 | 33,969 | 33,933 | 3,365  (1.6) | 6,822  (3.8) | 3,291  (1.6) | 6,693  (3.8) | 3,052  (1.5) | 6,074  (3.6) |
| Heart failure events n (%) | 30,957 | 28,393 | 30,287 | 25,679 | 1,795  (0.9) | 2,816  (1.6) | 1,762  (0.8) | 2,762  (1.6) | 1,557  (0.8) | 2,389  (1.4) |
| Total CVD events n (%) | 53,258 (2.8) | 53,403 (3.7) | 54,365 (2.8) | 50,489 (2.6) | 4,794  (2.3) | 8,836  (5.0) | 4,694  (2.3) | 8,674  (4.9) | 4,300  (2.1) | 7,797  (4.6) |
| Deaths n (%) | 84,289 | 80,897 | 82,555 | 76,783 | 280  (0.1) | 653  (0.4) | 275  (0.1) | 645  (0.4) | 259  0.1 | 565  (0.3) |

SCORE 2 MODEL

| Characteristics | Study | UKBiobank | | | | | | |
| --- | --- | --- | --- | --- | --- | --- | --- | --- |
|  |  | Prevent label | | | Score2 label | | Qrisk3 label | |
|  | 677,684 | Female=  222,978 | | Male=  182,571 | Female=  221,579 | Male=  181,187 | Female=  216,188 | Male=  173,358 |
| Mean (SD) age (years) | 57 (9) | 56(8) | | 56 (8) | 56 (8) | 56 (8) | 56 (8) | 56 (8) |
| Male sex | 300,735 (44%) |  | |  |  |  |  |  |
| Mean (SD) total cholesterol, (mmol/L) | 5.8 (1.1) | 5.9 (1.1) | | 5.6 (1.1) | 5.9 (1.1) | 5.6(1.1) | 5.9(1.1) | 5.6(1.1) |
| Mean (SD) HDL cholesterol (mmol/L) | 1.4 (0.4) | 1.6 (0.4) | | 1.3 (0.3) | 1.6 (0.4) | 1.3 (0.3) | 1.6 (0.4) | 1.3 (0.3) |
| Mean (SD) systolic blood pressure (mm Hg) | 136 (19) | 135 (19) | | 141 (17) | 135 (19) | 141.0(17) | 135(19) | 141.0(17) |
| Smoking status: |  |  | |  |  |  |  |  |
| Current smoker n (%) | 101,211 (15%) | 19,667 (8.8%) | | 22,499  (12.3%) | 19,437  (8.8%) | 22,211  (12.3%) | 18,911  (8.8%) | 21,403  (12.4%) |
| Diabetes mellitus n (%) | 31,413 (4.6%) | 5,140 (2.3%) | | 8,378  (4.6%) | 5,060  (2.3%) | 8,258  (4.6%) | 4,537  (2.1%) | 7,130  (4.1%) |
| Total CVD events n (%) | 30,121 (4.4%) | 4,366 (2.0%) | 7,958  (4.4%) | | 4,178  (1.9%) | 7,722  (4.3%) | 3,848  (1.8%) | 6,959  (4.0%) |
| Follow-up (years, median (5^th^/95^th^ percentile) | 10.7 (5.0-18.6) | 10.0  (9.5-10.0) | 10.0 (6.0-10.0) | | 10.0  (9.6-10.0) | 10.0  (6.0-10.0) | 10.0  (9.9-10.0) | 10.0  (6.3-10.0) |

In the descriptive statistics tables, the total number of CVD events, Atherosclerotic cardiovascular disease events (ASCVD) events, heart failure events, deaths, and follow-up times (where they exist) were reported according to the outcome definition specific to each model (e.g., QRISK3 model – QRISK3 outcome definition). Stratification by label indicates that individuals with a positive outcome prior to attendance, based on the respective outcome definition, were excluded from each dataset. For instance, in the context of the QRISK3 algorithm, individuals labeled under SCORE2 are those who did not experience an outcome before attendance according to the SCORE2 definition. In contrast, Table SD1 focuses on the rate of events when each algorithm is applied to different outcome definitions in turn.

**Figure SD1a**. Flowchart of the included participants using the QRISK3 model eligibility criteria


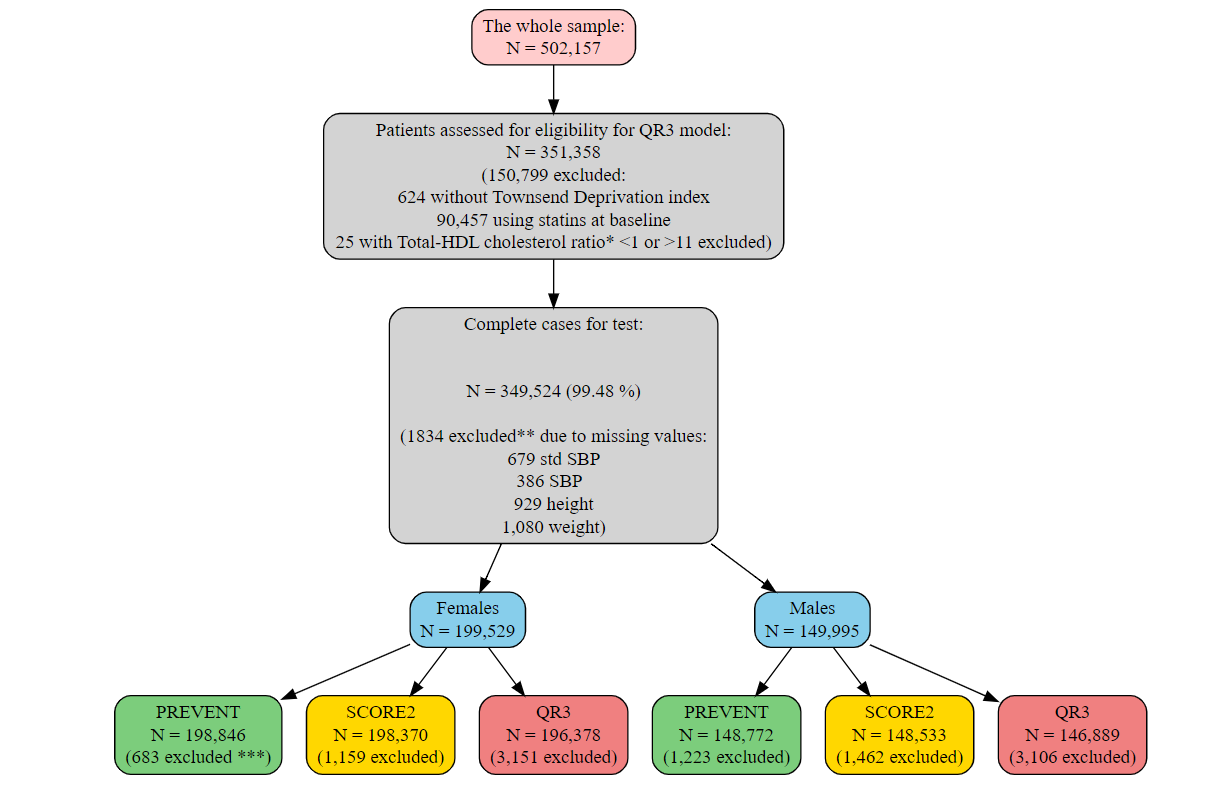


^[[1]](#footnote-1)^

**Figure SD1b**. Flowchart of the included participants using the PREVENT model eligibility criteria


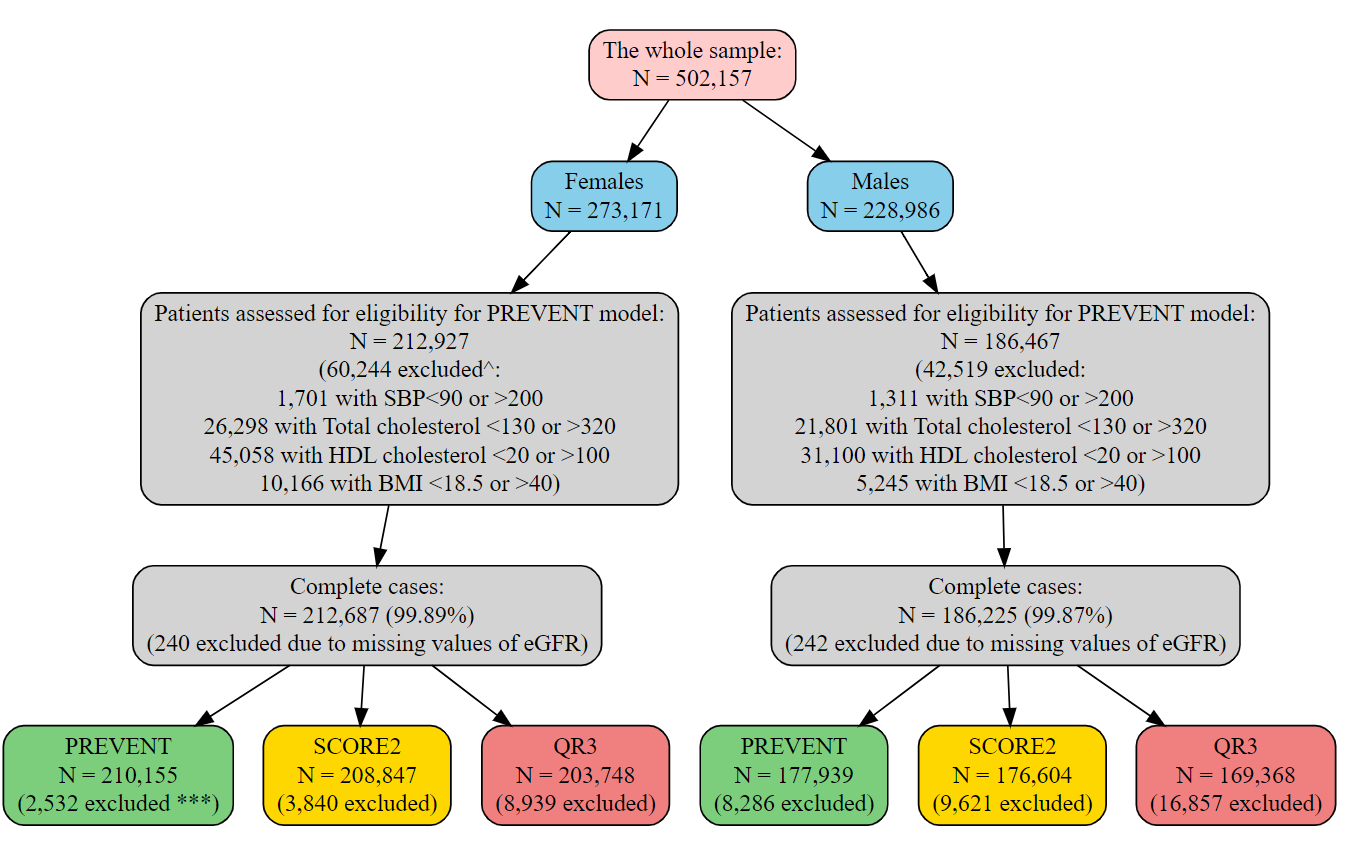
^[[2]](#footnote-2)^

**Figure SD1c**. Flowchart of the included participants using the SCORE2 model eligibility criteria


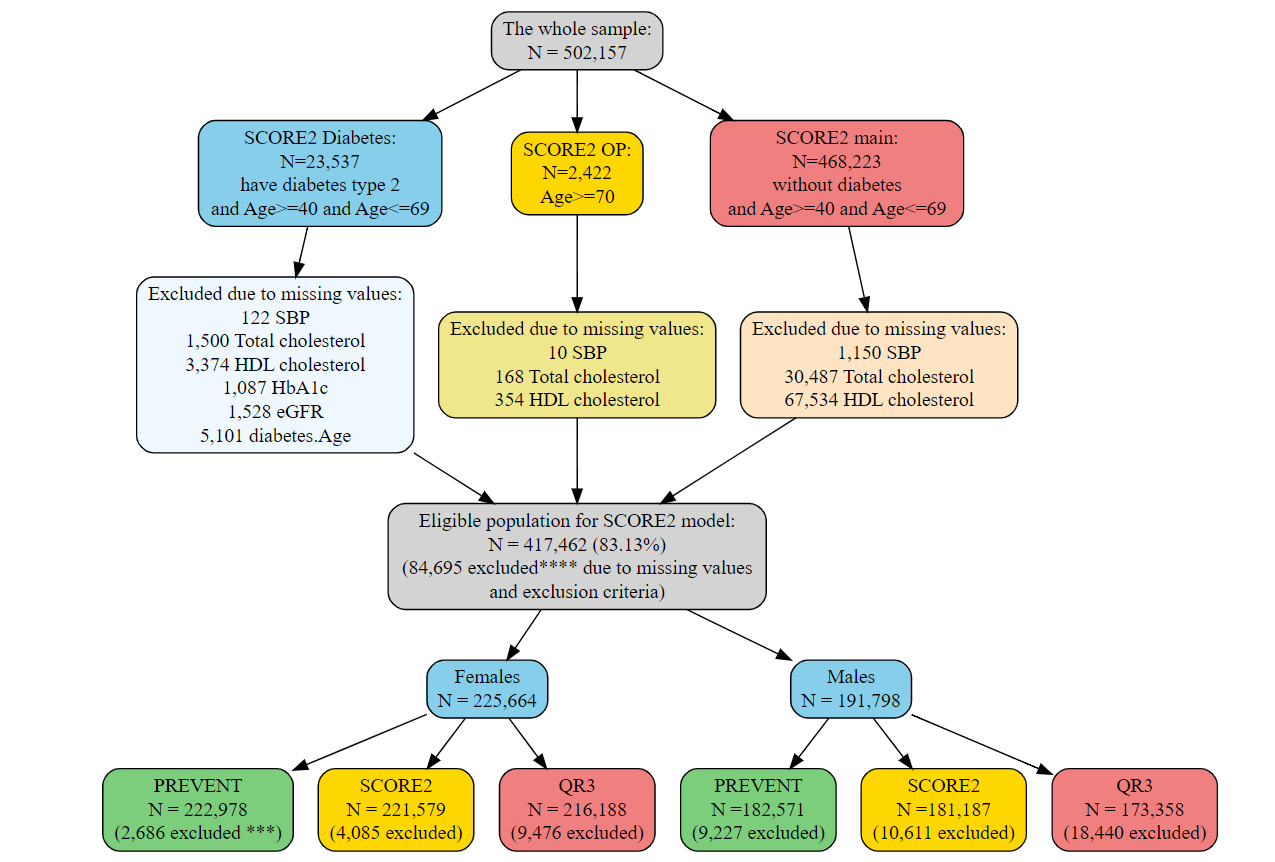


^[[3]](#footnote-3)^

1. * 72,657 had missing Total-HDL cholesterol ratio.

   ** 1,035 subjects had missing values in more than one variable.

   *** Exclusion of those with CVD at baseline according to the respective outcome definition. [↑](#footnote-ref-1)
2. ^ Excluded due to out-of-range values or missing data. [↑](#footnote-ref-2)
3. **** 7 individuals are under 40 years old, 7968 have diabetes at baseline but not diabetes type II and are 40 to 70 years old. 35,695 subjects have missing values in more than one variable. [↑](#footnote-ref-3)
